# Optimizing diabetic kidney disease animal models: Insights from a meta‐analytic approach

**DOI:** 10.1002/ame2.12350

**Published:** 2023-09-18

**Authors:** Fanghong Li, Zhi Ma, Yajie Cai, Jingwei Zhou, Runping Liu

**Affiliations:** ^1^ School of Chinese Materia Medica Beijing University of Chinese Medicine Beijing China; ^2^ School of Life Sciences Beijing University of Chinese Medicine Beijing China; ^3^ Department of Nephrology, Dongzhimen Hospital The First Affiliated Hospital of Beijing University of Chinese Medicine Beijing China

**Keywords:** animal model, diabetic kidney diseases, meta‐analysis

## Abstract

Diabetic kidney disease (DKD) is a prevalent complication of diabetes, often leading to end‐stage renal disease. Animal models have been widely used to study the pathogenesis of DKD and evaluate potential therapies. However, current animal models often fail to fully capture the pathological characteristics of renal injury observed in clinical patients with DKD. Additionally, modeling DKD is often a time‐consuming, costly, and labor‐intensive process. The current review aims to summarize modeling strategies in the establishment of DKD animal models by utilizing meta‐analysis related methods and to aid in the optimization of these models for future research. A total of 1215 articles were retrieved with the keywords of “diabetic kidney disease” and “animal experiment” in the past 10 years. Following screening, 84 articles were selected for inclusion in the meta‐analysis. Review manager 5.4.1 was employed to analyze the changes in blood glucose, glycosylated hemoglobin, total cholesterol, triglyceride, serum creatinine, blood urea nitrogen, and urinary albumin excretion rate in each model. Renal lesions shown in different models that were not suitable to be included in the meta‐analysis were also extensively discussed. The above analysis suggested that combining various stimuli or introducing additional renal injuries to current models would be a promising avenue to overcome existing challenges and limitations. In conclusion, our review article provides an in‐depth analysis of the limitations in current DKD animal models and proposes strategies for improving the accuracy and reliability of these models that will inspire future research efforts in the DKD research field.

## Graphical abstract

In this paper, 84 studies were found using the keywords “diabetic kidney disease” and “animal model” in the Pubmed database. Then the diabetic metabolic indicators and renal function indicators in the articles were collected for meta‐analysis. Combined with the renal lesions and modeling information, the conclusion of optimizing animal models by integrating various stimuli was reached.

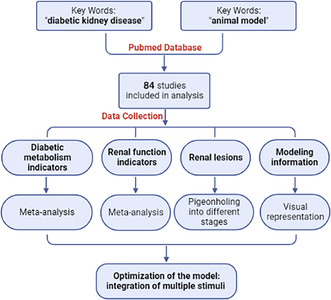

## 1 INTRODUCTION

Diabetic kidney disease (DKD) is a type of glomerulosclerosis induced by diabetic metabolic abnormalities, and is also a manifestation of systemic microangiopathy. DKD affects approximately 20%–40% of patients with type 1 or type 2 diabetes, and is a leading cause of end‐stage renal disease.^1^ Clinical symptoms of diabetic kidney disease include persistent albuminuria, elevated blood pressure, and decreased glomerular filtration rate (GFR). The Global Burden of Disease Survey revealed that the age‐standardized prevalence of DKD for males and females is 15.48/1000 and 16.50/1000, respectively.^2^ The pathogenesis of diabetic kidney disease is multi‐factorial. Advanced glycation end products (AGEs), inflammatory factors, fibrosis, renin, blood lipids and reactive oxygen species are all critical contributors to the pathogenesis of diabetic nephropathy. Researches on the pathophysiology of DKD have been very scarce, which has resulted in a dearth of effective therapy options. The current approach to managing DKD involves strict blood glucose control and medication that targets the renin‐angiotensin system. To facilitate the development of novel medicines, further study on therapeutic targets and underlying pathologies is required. However, currently used DKD animal models can only simulate a portion of human pathophysiological alterations, but not the entire etiology and process, and there is still a substantial gap between DKD animal models and human DKD patients. Hence, creating an efficient and trustworthy animal model that faithfully reflects the characteristics of clinical patients represents the first challenge in studying DKD.

An ideal diabetic kidney disease model should display all of the following: (1) 50% degeneration of kidney function, (2) more than 10‐fold increase in proteinuria, (3) pathological injuries, including mesangial stroma dilatation, glomerular basement membrane thickening, and tubulointerstitial fibrosis.^3^ With the development of animal model research, more and more animal models have been used in DKD research, including chemical models and genetic models. Despite the significant contributions of current animal models in DKD research, they still have limitations in fully capturing the complexity of the disease across different stages. More comprehensive animal models are necessary to fully define the evolving landscape of chronic disease associated with DKD. Furthermore, no studies have systematically analyzed the advantages and disadvantages of the currently used animal models and their pathological characteristics, which makes the selection and optimization of basic DKD animal models difficult.

To address the above issue, we perform a meta‐analysis on previously reported DKD animal models incorporating the glucose and lipid metabolism, and renal function indexes. Because histopathological indicators could not be incorporated into the meta‐analysis, data extraction tools were employed to obtain comprehensive qualitative data. On this basis, by analyzing the genetic models, dietary factors, drug administration, and the duration of modeling, we established correlations between modeling parameters and DKD characteristics. Hence, the current study not only identifies appropriate DKD animal models for both pathological and pharmacological studies but also provides valuable approaches for the further optimization of these models.

## 2 METHODS

### 2.1 Search strategies

To ensure the standardization and reproducibility of the results, we used the MeSH terms and/or keywords including “diabetic nephropathies” OR “diabetic kidney disease”, AND “animals not humans” OR “mice” OR “rats” OR “mouse” OR “rat”, AND “animal experimentation”. Our search was limited to animal experiments published in PubMed databases between August 8, 2012 and August 8, 2022 in English. After several rounds of preliminary searching, we arrived at a final search strategy, outlined in Supplementary Table 1, that aimed to encompass as many relevant animal models as possible.

### 2.2 Study selection and exclusion criteria

#### 2.2.1 Eligibility criteria

The eligibility criteria for our analysis were as follows: (1) ideally the research on diabetic kidney disease should involve an experimental animal model unrestricted by strain, species, gender, or weight; (2) studies should include a control group and a model group for comparison; (3) the time frame for model building and the duration should be clearly stated in the studies; (4) the predetermined outcomes should include: diabetes metabolism indicators (blood glucose, HbA1c), lipid metabolism indicators (triglyceride, total cholesterol), renal function indicators (serum creatine, blood urea nitrogen, urinary albumin excretion); (5) the language is limited to English.

#### 2.2.2 Exclusion criteria

Studies excluded from our analysis included: (1) cell research; (2) studies not using experimental DKD models; (3) reviews or meta‐analysis; (4) studies not in the PubMed directory; (5) conference abstracts, comments or interviews; (6) randomized controlled trials, clinical trials or in vitro studies; (7) studies where the full text was not available; (8) studies with no determined primary outcome or lack of a control group.

### 2.3 Data collection and conversion

The baseline characteristics of each study comprised the following: (1) author name, and year of publication; (2) animal strain, species, age, gender, weight, genotype, and sample size in model and control groups; (3) method of model induction (administration route, dosage, frequency and duration); (4) outcome measures (blood glucose, HbA1c, total cholesterol, triglyceride, UAE, BUN, and SCR); (5) the measurement time of the above indexes; (6) basic index (fibrosis area, collagen area, GBM thickness, GFR, tubular injury score, mesangial matrix index, glomerulosclerosis score, and glomerular size).

Data extraction was conducted on all outcomes, including the sample size of animals (*N*), means, standard deviation (SD) or standard error of the mean (SEM). Data presented graphically was extracted using digital image analysis software (WebPlotDigitizer). SEM was converted to SD using the mathematical formula, SD = SEM*$\sqrt{N}$. Sample size reported as a range was represented by its median. When the quantity was not mentioned, we took the most conservative estimate of *N* (*N* = 6).

### 2.4 Quality assessment

In accordance with practical requirements, to be eligible for inclusion in the meta‐analysis an article must include the following: (1) the control group of DKD model; (2) the criteria for modeling successfully; (3) basic information on both control and model groups (species, genotype, gender, age, etc.); (4) DM indicators for the control and model groups (blood glucose, HbA1c); (5) lipid metabolism indicators for the control and model groups (TG, TC); (6) renal function indicators for the control and model groups (serum creatine, serum BUN, urinary albumin); (7) the treatment and diet type of the control and model groups; (8) histopathological changes during disease progression; (9) the measurement time of the selected outcome; (10) random allocation to treatment or control. The results of the assessment were scored as Yes (1), No (0), or Not perfect (0.5) based on the presence or absence of the above‐mentioned criteria (Table 1).

**TABLE 1:** Quality assessment of included studies.

Study	(1)	(2)	(3)	(4)	(5)	(6)	(7)	(8)	(9)	(10)	Total
Ali et al.^47^	1	1	0.5	0.5	1	0.5	1	0.5	1	1	8
Alomari et al.^54^	1	1	0.5	0.5	0	0.5	1	0.5	1	1	7
Alzahrani et al.^55^	1	1	0.5	0.5	0	0.5	1	0.5	1	1	7
Amin et al.^46^	1	1	0.5	0.5	0	0.5	1	0.5	1	1	7
Arimura et al.^56^	1	0	1	1	0	0.5	1	0	1	1	6.5
Barati et al.^37^	1	0.5	0.5	0.5	0.5	0.5	1	0.5	1	1	7
Björnson et al.^33^	1	0	0.5	1	0.5	0.5	1	0.5	1	1	7
Cassis et al.^34^	1	0.5	0.5	0.5	1	0.5	1	0.5	1	1	7.5
Chen et al.^57^	1	1	0.5	0	0	0.5	1	0.5	1	1	6.5
Chen et al.^45^	1	1	1	0.5	1	0.5	1	0.5	1	1	8.5
Chodavarapu et al.^58^	1	0	0.5	0.5	0.5	0	1	0.5	1	1	6
Christensen et al.^59^	1	1	0.5	0.5	0	0.5	1	0	1	1	6.5
Couto et al.^60^	1	1	0.5	0	0	0.5	1	0	1	1	6
Ding et al.^61^	1	1	0.5	0.5	1	0	1	0.5	1	1	7.5
Domon et al.^62^	1	1	0.5	0	0.5	0.5	1	0.5	1	1	7
Dong et al.^63^	1	1	0.5	0	1	0.5	1	0.5	1	1	7.5
Dschietzig et al.^64^	1	1	0.5	0.5	0	0	1	0.5	1	1	6.5
Dusabimana et al.^29^	1	0	0.5	0.5	0	0.5	1	0.5	1	1	6
Elseweidy et al.^65^	1	1	1	0.5	0	0.5	1	0.5	1	1	7.5
Faisal Lutfi et al.^66^	1	1	0.5	0.5	1	0.5	1	0.5	1	1	8
Fernandes et al.^44^	1	1	1	0	0	0.5	1	0.5	1	1	7
Formigari et al.^67^	1	1	0.5	0	0	0.5	1	0.5	1	1	6.5
Franzén et al.^68^	1	1	0.5	0.5	0	0	1	0.5	1	1	6.5
Gad^69^	1	1	1	0.5	1	0	1	0	1	1	7.5
Gargouri et al.^70^	1	1	1	0.5	1	0.5	1	0.5	1	1	8.5
Garud and Kulkarni^71^	1	1	1	0.5	0	0.5	1	0.5	1	1	7.5
Glastras et al.^31^	1	1	1	0.5	1	0	1	0.5	1	1	8
Glastras et al.^31^	1	1	0.5	1	0.5	0.5	1	0.5	1	1	8
Goru et al.^72^	1	1	1	0.5	0	0.5	1	0.5	1	1	7.5
Habibi et al.^73^	1	1	0.5	0.5	1	0.5	1	0.5	1	1	8
Han et al.^50^	1	1	0.5	0.5	0	0.5	1	0.5	1	1	7
He et al.^25^	1	0	0.5	1	0	0.5	1	0.5	1	1	6.5
Hong et al.^74^	1	1	0.5	0	0	0.5	1	0.5	1	1	6.5
Hu et al.^49^	1	1	1	0.5	1	0.5	1	0.5	1	1	8.5
Huang et al.^41^	1	1	1	0.5	0	0.5	1	0.5	1	1	7.5
Huang et al.^75^	1	1	0.5	0	1	0.5	1	0.5	1	1	7.5
Ibrahim and Abd^76^	1	1	0.5	0.5	0	0.5	1	0	1	1	6.5
Ibrahim et al.^77^	1	1	0.5	0	0.5	0.5	1	0.5	1	1	7
Itano et al.^36^	1	0	1	1	0	0.5	1	0.5	1	1	7
Jeong et al.^78^	1	0	0.5	0.5	0	0.0.5	1	0.5	1	1	6
Kim et al.^79^	1	0	0.5	0	0	0.5	1	0.5	1	1	5.5
Klein et al.^80^	1	0	0.5	1	0	0	1	0.5	1	1	6
Ladeira et al.^81^	1	1	1	0.5	0	0	1	0.5	1	1	7
Lezcano et al.^38^	1	0	1	0.5	1	0.5	1	0	1	1	7
Li et al.^32^	1	0	0.5	1	1	0.5	1	0	1	1	7
Li et al.^82^	1	0	0.5	0.5	0	0	1	0	1	1	5
Li et al.^83^	1	1	1	0	0	0.5	1	0.5	1	1	7
Li et al.^39^	1	0	0.5	0.5	0	0	1	0.5	1	1	5.5
Lian et al.^84^	1	1	0.5	0.5	0	0.5	1	0	1	1	6.5
Liu et al.^85^	1	0	0.5	0.5	0	0	1	0.5	1	1	5.5
Liu et al.^28^	1	0	0.5	0.5	0	0.5	1	0.5	1	1	6
Lopez‐Parra et al.^86^	1	1	0.5	0.5	0.5	0.5	1	0.5	1	1	7.5
Ma et al.^53^	1	0	0.5	0.5	0.5	0.5	1	0.5	1	1	6.5
Maheshwari et al.^42^	1	1	0.5	0	0	0.5	1	0.5	1	1	6.5
Mohamed et al.^87^	1	0	0.5	0.5	0	0.5	1	0.5	1	1	6
Moon et al.^88^	1	0	0.5	0	0.5	0.5	1	0.5	1	1	6
Morigi et al.^40^	1	0.5	0.5	0.5	1	0	1	0.5	1	1	7
Nasri et al.^89^	1	1	1	0	1	0.5	1	0.5	1	1	8
Ndisang and Jadhav^90^	1	1	0.5	0	0	0.5	1	0.5	1	1	6.5
Nordquist et al.^91^	1	1	1	0.5	0	0	1	0.5	1	1	7
Nunes et al.^92^	1	0	0.5	1	0	0.5	1	0.5	1	1	6.5
Pichaiwong et al.^35^	1	0	0.5	0.5	0	1	1	0.5	1	1	6.5
Sadar et al.^26^	1	1	0.5	0.5	0	1	1	0.5	1	1	7.5
Sathibabu et al.^93^	1	1	0.5	0	0	0.5	1	0.5	1	1	6.5
Shiju et al.^27^	1	1	1	1	1	1	1	0.5	1	1	9.5
Somineni et al.^94^	1	0	0.5	0.5	1	0	1	0.5	1	1	6.5
Souza et al.^95^	1	1	0.5	0	0	0.5	1	0.5	1	1	6.5
te Riet et al.^24^	1	1	0.5	0.5	0	0.5	1	0.5	1	1	7
Thibodeau et al.^23^	1	0	0.5	0.5	0	0	1	0.5	1	1	5.5
Toyoda et al.^21^	1	0	0.5	0.5	1	0	1	0.5	1	1	6.5
Tung et al.^96^	1	1	0.5	1	0	0	1	0.5	1	1	7
Uil et al.^48^	1	1	1	0	0	0.5	1	0.5	1	1	7
Wang et al.^52^	1	0	0.5	0	0	0.5	1	0.5	1	1	5.5
Wang et al.^97^	1	1	0.5	0.5	0	0.5	1	0.5	1	1	7
Wang et al.^98^	1	1	0.5	0.5	0	0.5	0	0.5	1	1	6
Wang et al.^99^	1	0	0.5	1	1	0.5	1	0.5	1	1	7.5
Xin et al.^100^	1	1	0.5	0.5	0	0.5	1	0.5	1	1	7
Xu et al.^101^	1	0	0.5	0.5	0	0.5	1	0.5	1	1	6
Yang et al.^102^	1	1	1	0.5	0	0.5	1	0.5	1	1	7.5
Ying et al.^103^	1	1	0.5	0	1	0.5	1	0.5	1	1	7.5
Zakaria et al.^104^	1	1	1	0	0	0.5	1	0.5	1	1	7
Zhang et al.^51^	1	0	0.5	0.5	0.5	0.5	1	0.5	1	1	6.5
Zhang et al.^16^	1	1	0.5	0	0	0.5	1	0.5	1	1	6.5
Zhang et al.^105^	1	0	0.5	1	0	0.5	1	0.5	1	1	6.5

Abbreviations: (1) Vehicle control of DKD model; (2) the criteria for modeling successfully; (3) basic information about the control and model groups(species, genotype, sex, age, etc); (4) DM indicator about the control and model groups (blood glucose, HbA_1_C); (5) lipid metabolism indicator about the control and model groups (TG, TC); (6) renal function indicator about the control and model groups(serum creatine, serum Bun, Urinary albumin); (7) the treatment and diet type about the control and model groups; (8) histopathological changes during disease progression; (9) the measurement time of selected outcome; (10) random allocation to treatment or control; 1, Yes; 0, No; 0.5, Not perfect.

### 2.5 Statistical analysis

We used Revman 5.4 for data analysis. Continuous variables (blood glucose, HbA1c, TG, TC, UAE, SCR, BUN) were expressed as the standard mean difference with a 95% confidence interval (CI). Heterogeneity was evaluated using *I* ^2^ with a random‐effects model applied if *I* ^2^ > 50%, and a fixed‐effects model is applied if *I* ^2^ < 50%. Subgroup analysis was performed based on various model types (transgenic mice, transgenic rats, treatment mice, treatment rats, transgenic mice combined with treatment, and transgenic rats combined with treatment). Subgroup analysis was integrated with basic model information to assess the weaknesses and strengths of each model. Funnel plots were used to assess potential publication bias for outcome measures.

### 2.6 Publication bias test

The publication bias funnel indicated that there was substantial publication bias in blood glucose, HbA1c, TC, TG, SCR, BUN and UAE (Supporting Information Figure S1).

## 3 RESULTS

### 3.1 Included studies

**FIGURE 1:**
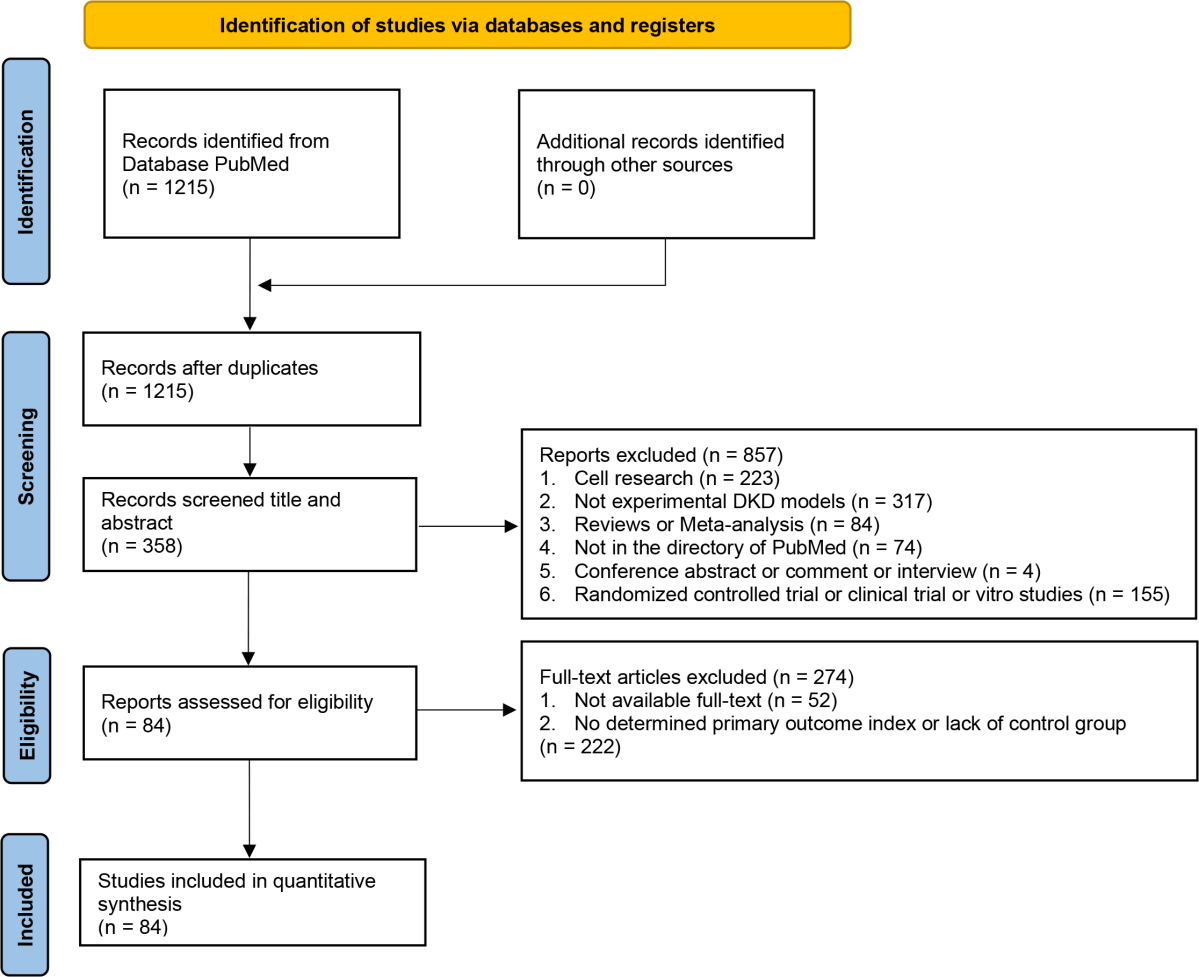
Flow diagram of the screening process.

A comprehensive search of the PubMed database yielded a total of 1215 studies. After duplicate removal, titles and abstracts were screened and evaluated for eligibility based on the full‐text articles, and we excluded 223 in vitro studies, 317 non‐experimental DKD models, 84 reviews or meta‐analysis, 74 unavailable in PubMed, 4 conference abstracts, comments or interviews, and 155 clinical studies. Ultimately, 84 studies were included in the meta‐analysis for further independent evaluation of methodological quality (Supplementary File 1). The selection process is summarized in a flow diagram illustrated in Figure 1.

### 3.2 A brief introduction to the DKD animal models in the included studies

The animal models of DKD selected for analysis can be divided into two major categories: (1) chemical‐induced models, and (2) genetic models. At present, the most common inducible animal models are established using streptozotocin (STZ) and alloxan as primary treatments. STZ and alloxan both show great selective toxicity for islet cells, impairing glucokinase function and generating DNA double‐strand breaks, which kills islet cells with high specificity, affects intracellular insulin manufacture, and leads to insulin deficiency.^4^ In the case of single‐drug induced DKD models, high‐fat diet or high‐fat and high‐sugar diet, unilateral or partial nephrectomy and other methods are commonly used as adjuvant challenges to accelerate the progression of diabetic nephropathy.^5^ High‐fat diet lowers skeletal muscle glucose absorption^6^ while inhibiting liver GLc6Pase activity to enhance glycogen retention in the liver.^7^ It also lowers PPARγ coactivator‐1α (PGC‐1α) expression, a transcription factor involved in mitochondria, which degrades mitochondrial function^8^ and generates metabolic syndrome in type 2 diabetic patients, including obesity, hyperglycemia, hyperlipidemia, and insulin resistance. A high‐fat and high‐sugar diet can further enhance the reactivity of angiotensin II, angiotensin converting enzyme, endothelin and other vasoactive factors with high blood glucose, and promote vascular permeability, resulting in high renal corpuscular perfusion, high internal pressure and high filtration, leading to the occurrence of proteinuria.^9^ At the same time, elevated blood glucose can activate protein kinase C and produce glycosylation end products, which increases the filtration rate of renal corpuscles and aggravates albuminuria. The production of transforming growth factor‐β in the kidney increases, which increases the deposition of extracellular collagen, collagen IV, collagen V, collagen VI and cellulose, leading to mesangial expansion, renal corpuscle basement membrane thickening and other renal damage.^10^ In conjunction with this foundation, a low dose of STZ can be used to create an animal model of type 2 diabetes. Sometimes unilateral or nephrectomy is combined with single‐drug induction in order to aggravate the renal lesions alone.

Compared to the chemical‐induced models, genetic models of DKD offer a wider range of options with different modeling mechanisms. Common genetic type 1 DM models include Ins‐2 Akita mice and transgenic OVE26 mice. Ins2‐Akita mice have a mutation (C96Y) in Ins‐2 gene, which causes the loss of disulfide bond formation between the A and B chains of insulin‐2. This mutated Insulin‐2 further produces proteotoxicity in pancreatic β cells, leading to the reduction of β cell mass and the weakening of insulin secretion ability.^11^ The transgenic OVE26 mouse is a type 1 DM animal model established by transferring the insulin2 enhancer‐driven calmodulin gene, so that the calmodulin protein is highly expressed in islet β cells, leading to insulin secretion disorder.^12^

Type 2 DM is characterized by insulin resistance, which is more easily achieved than the absolute insulin insufficiency seen in type 1 DM, so there are more genetic type 2 DM models. KK‐A^y^ mice, ob/ob mice, db/db mice, eNOS‐KO mice, ZnT8‐KO‐db/db mice, Zucker diabetic fatty (ZDF) rats, spontaneously diabetic torri (SDT) rats and LEA.PET‐pet congenic strain rats are common examples. The most widely used animal models for type 2 DM are db/db mice and ob/ob mice, which have also been used extensively in research on diabetic nephropathy. The ob/ob mice are created by mutation of the ob gene on chromosome 6, resulting in a deficiency of leptin protein. This deficiency leads to significantly increased hepatic gluconeogenesis and hyperglycemia, which then stimulates abnormal insulin secretion, and finally develops insulin resistance.^13^ To exacerbate the disease, ob/ob mice can be crossed with BTBR mice, which are spontaneously insulin resistant, to create BTBR ^ob/ob^ animals. In BTBR^ob/ob^ mice, sustained hyperglycemia can be obtained at an early stage, and morphological and functional changes similar to the late stages of human DKD can appear over a relatively short period of time.^14^ Similar to ob/ob mice, db/db mice are a model of congenital obesity and type 2 diabetes due to a mutation in the db gene on chromosome 4, which causes abnormal transcription of leptin receptor. The course of diabetes in db/db mice is very similar to that in humans, and db/db mice are prone to microvascular complications, especially diabetic kidney disease.^15^ Zinc transporter 8 (ZnT8) transports zinc ions for crystallization and storage of insulin in pancreatic beta‐cells. ZnT8‐KO male mice were subsequently intercrossed with female db/db mice to generate ZnT8‐KO‐db/db mice, causing a more severe type2 DM along with insulin resistance.^16^

KK mice were developed from Kasukabe mice by K. Kondo in 1957 and exhibit insulin resistance and obesity, with albuminuria at 10 to 15 weeks of age. The A^y^ gene not only affects the hair color of mice, but also causes metabolic disorders, which manifest as obesity, hyperglycemia, lipid metabolism disorders and hyperinsulinemia.^17^ KK‐A^y^ mice were established by introducing the A^y^ gene into KK mice, to produce another commonly used type 2 diabetes model. Compared with KK mice, KK‐A^y^ mice have severe obesity and hyperglycemia in the early stage, and the kidney injury of KK‐A^y^ mice is very similar to that of human type 2 diabetes.^18^ eNOS is the rate‐limiting enzyme of nitric oxide (NO) synthesis. Abnormal or absent functions of eNOS will weaken the function of NO in regulating vascular system permeability and cause vascular endothelial cell injury. Endothelial injury is an important mechanism and pathological feature of vascular lesions in DM and DKD.^19^ Therefore, eNOS‐KO mice are currently a mouse model that mimics the functional and morphological changes of human DKD at an advanced stage.

In addition, rats can also be used to establish type 2 DM animal models. The Zucker Diabetic Fatty (ZDF) rat is a common model created through mutation in the leptin receptor gene, leading to hyperphagia and obesity, accompanied by hyperinsulinemia, hyperglycemia, peripheral insulin resistance, abnormal lipid metabolism, moderate hypertension, and proteinuria. However, this model does not exhibit ketosis.^20^ There are also genetic rat models that can spontaneously develop a diabetes‐like phenotype, although the precise underlying genotype is not well elucidated. The Spontaneously Diabetic Torii (SDT) rat model is based on the principle that deformation of pancreatic beta‐cells leads to impaired insulin secretion, resulting in the development of a type 2 diabetes‐like phenotype.^21^ Congenital polydipsia, polyuria, and hyperglycemia were also discovered in rats belonging to the LEA.PET‐pet congenic strain. Consequently, a unique rat strain was developed, which displays non‐obese type 2 diabetes and enlarged kidneys.

The pathogenesis and development of diabetic nephropathy have been extensively linked to the renin‐angiotensin‐aldosterone system (RAS). RAS hyperactivation can lead to the production of excess oxygen free radicals, which damage endothelial function and impair microcirculation, and thus is thought to contribute to the kidney injuries associated with diabetes.^22^ Based on this theory, the transthyretin promoter (TTRhRen)^23^ and mRen2^24^ were transferred into mice with spontaneous diabetes to induce renin overexpression, utilizing the twin stacked effects of renin‐dependent hypertension and diabetes to manifest many essential characteristics of human DKD. In certain studies, male OVE26 mice were crossed with female TTRhRen mice to obtain OVE26‐TTRhREN double transgenic mice, showing dual additive effects of renin‐dependent hypertension and diabetes.^23^ Notably, the mechanism of model establishment in transgenic (mRen‐2)27 rats was the same as that in OVE26‐TTRhRen double gene rats. The model of Akita^+/−^ Ren^+/−^ also relies on the dual additive effects of renin‐dependent hypertension and diabetes.^25^

### 3.3 Effectiveness

#### 3.3.1 Relationships between modeling method and metabolic phenotypes in diabetes mellitus

Diabetic nephropathy, as a major complication of diabetes mellitus, is associated with, and probably the result of, metabolic disorders such as obesity, hyperglycemia, and hyperlipidemia in diabetic patients. Therefore, the relationship between modeling methods and diabetic metabolic phenotype was analyzed from the aspects of glucose metabolism and lipid metabolism. Triglycerides and total cholesterol were used as reference indices for lipid metabolism, blood glucose and glycosylated hemoglobin were used as reference indices for glucose metabolism, and subgroup analysis was carried out, respectively (Supporting Information Figures S2–S5).

For chemical‐induced models, SD and Wistar are the most frequently used rat strains, and the mouse strains used for modeling include C57BL/6, BALB/C, 129SV, NMRI, etc. As can be seen from the Table 2, an intraperitoneal dose of STZ in rats, generally between 45 and 60 mg/kg, resulted in a considerable increase in blood glucose, TG, and TC. However, the change in HbA1c was minimal. Considering the changes in the above indictors in the two strains, it was not difficult to determine that Wistar rats are more sensitive to STZ and alloxan than SD rats. Additionally, STZ outperformed alloxan in terms of modeling effect at the same dose. Unlike in rats, the administration regimens for STZ in mice are more varied, consisting of multiple high‐dose injections, a single high‐dose injection, multiple low‐dose injections and a single low‐dose injection. The data in the figures further demonstrate that the relationship between blood glucose level and STZ dosage and frequency is linear. Changes in HbA1c levels were generally modest and were not significantly affected by the administration regimen of STZ. TG and TC only showed a slight change after a single high‐dose administration. The sensitivity of various mouse strains to STZ is still unclear, as there is no research in the included literature on using STZ to induce diabetes in other strains of mice. However, combined with the analysis results on glucose metabolism and lipid metabolism, the sensitivity of different mouse strains to alloxan was in the order of C57BL/6 > BALB/C > 129SV > NMRI. In general, rats are more sensitive to chemical agents and have a greater modeling rate than mice, as evidenced by the fact that after receiving a single low‐dose injection of STZ, their levels of lipid and glucose metabolism increased quickly compared to mice.

**TABLE 2:** The chart of the relationship between modeling conditions and diabetic metabolism indices.

	Strain	Genotype	Age	Treatment	Surgery	Diet	Molding time(d)	BG	HbA1c	TC	TG
Genetic mouse models	BTBR, NR	ob/ob	NR	NR	–	Chow diet	63	32.85		1.22	1.63
	C57BLKS/J, male	db/db	8 weeks	NR	–	Chow diet	70	26.91	4.59	2.03	
	FVB/n‐C57BL/6, male	Akita^+/−^ Ren^+/−^	NR	NR	–	Chow diet	42	23.28	36.64		
	C57BL/6J, male	eNOS^−/−^	8 weeks	i.p. STZ (55 mg/kg) for 5 d	–	Chow diet	3	16.6			
	C57BL/6, male	Ins2	9 weeks	NR	–	Chow diet	63	15.11	4.31		
	C57BL/KsJ, male	db/db	NR	i.h. injection of casein (0.5 mL 10%)	–	Chow diet	112	11.55		6.66	1.38
	C57BL/KsJ, male	db/db	NR	NR	–	Chow diet	56	10.66		5.93	1.73
	C57BLKS/J, male	db/db	8 weeks	NR	–	Chow diet	56	9.16	4.64	1.85	2.76
	BTBR, male	ob/ob	NR	NR	–	Chow diet	70	6.3		1.18	2.27
	C57BLKS/J, male	db/db	4 weeks	NR	–	Chow diet	35	5.92			
	FVB/N, male	TTRhRen	8–12 weeks	A single i.p. injection of STZ (50 mg/kg)	–	Chow diet	56	5.73			
	C57BL/Ks, male	db/db	6 weeks	NR	–	Chow diet	49	5.66		0	2.33
	C57BLKS/J, male	db/db	NR	NR	–	Chow diet	70	5.06			2.06
	C57BL/KsJ, male	db/db	6 weeks	NR	–	Chow diet	42	3.67			3.79
	C57BLKS, male	db/db	4 weeks	NR	–	High protein diet	77	3.08	9.5	5.54	
	BTBR, female	ob/ob	4–6 weeks	NR	–	High protein diet	98	2.93			
	C57BL/6J, male	eNOS^−/−^	8 weeks	i.p. injection of STZ (100 mg/kg) for 2 d	–	Chow diet	28	2.84			
	C57BL/6, male	TauT^+/+^	7–8 weeks	i.p. injection of STZ (55 mg/kg) for 5 d	–	Chow diet	7	2.78			
	C57BLKS, male	db/db	NR	NR	–	Chow diet	56	2.43	7.4		
	C57BL/Ks, male	db/db	NR	NR	–	Chow diet	84	2.31			
	BTBR, male	ob/ob	NR	NR	UNx	Chow diet	126	2.27	8.81		
	BTBR, NR	ob/ob	NR	NR	–	Chow diet	126	2.13			
	C57BL/KS, male	db/db	6 weeks	NR	–	Chow diet	56	1.91	3.53	5.05	
	BTBR, female	ob/ob	4–6 weeks	NR	–	Chow diet	98	1.79	5.57		
	C57BL/6, male	TauT^+/+^	7–8 weeks	i.p. injection of STZ (55 mg/kg) for 5 d	–	Chow diet	7	1.72			
	C57BL/6, male	TauT^−/−^	7–8 weeks	i.p. injection of STZ (55 mg/kg) for 5 d	–	Chow diet	7	1.64			
	C57BL/6, male	TauT^−/−^	7–8 weeks	i.p. injection of STZ (55 mg/kg) for 5 d	–	Chow diet	7	1.55			
	C57BL/6, male	TauT^+/−^	7–8 weeks	i.p. injection of STZ (55 mg/kg) for 5 d	–	Chow diet	7	1.01			
	C57BL/Ks, male	db/db	NR	NR	–	Chow diet	84	0.92			
	C57BL/Ks, male	db/db	6 weeks	NR	–	Chow diet	56		13.26		
	Akita, male	Ins2 ^Akita/+^ Jak2^loxP/loxP^	NR	A single injection of Ang II (700 ng/min/kg)	–	Chow diet	70		12.53	1.71	
	KK‐Ta, male	Ay	8 weeks	NR	–	Chow diet	49		3.7		
Genetic rat models	Jcl:SDT, male	NR	NR	NR	–	Chow diet	189	31.23		1.22	2.66
	Jcl:SDT, male	NR	NR	NR	–	High fructose diet	217	12.85		1.93	3.77
	Ren2, NR	(mRen2)27	NR	A single i.p. injection of STZ (55 mg/kg)	–	Chow diet	21	9.27			
	Zücker, male	NR	8 weeks	NR	–	Chow diet	98	5.47		−0.09	1.13
	Zucker, male	NR	NR	NR	–	Chow diet	112		0.96	2.07	1.81
	LEA.PET, male	NR	NR	NR	–	Chow diet	70			−1.95	
Chemical‐induced mouse models	C57BL/6, male	NR	10 weeks	i.p. STZ (125 mg/kg) for 2 d	–	Chow diet	3	34.81		0.8	
	FVB, male	NR	NR	i.p. STZ (50 mg/kg) for 5 d	–	Chow diet	19	33.99	4.47		
	C57BL/6, male	NR	6–7 weeks	A single i.p. injection of STZ (180 mg/kg)	–	Chow diet	3	11.34			
	C57BL/6, male	NR	NR	i.p. STZ (55 mg/kg) for 5 d	–	Chow diet	8	8.64			
	C57BL/6J, NR	NR	NR	A single i.p. injection of STZ (100 mg/kg)	–	Chow diet	7	5.99			
	C57BL/6, male	NR	8 weeks	A single i.v. injection of alloxan (75 mg/kg)	–	Chow diet	14	5.82			
	BALB/c, male	NR	8 weeks	A single i.v. injection of alloxan (67 mg/kg)	–	Chow diet	14	4.18			
	C57BL/6, male	NR	7 weeks	A single i.p. injection of STZ (100 mg/kg)	UNx	High fat diet	24	4.08			
	C57BL/6, male	NR	3 weeks	i.p. injection of STZ (55 mg/kg) for 5 d	–	Chow diet prior to mating	5	3.4	7.3		0.1
	BALB/c, male	NR	8 weeks	A single i.v. injection of alloxan (67 mg/kg)	–	Chow diet	14	3.25			
	129Sv, male	NR	8 weeks	A single i.v. injection of alloxan (75 mg/kg)	–	Chow diet	14	3.01			
	C57Bl/6, male	NR	8 weeks	A single i.v. injection of alloxan (75 mg/kg)	–	Chow diet	14	2.8			
	NMRI, male	NR	8 weeks	A single i.v. injection of alloxan (75 mg/kg)	–	Chow diet	14	2.77			
	NMRI, male	NR	8 weeks	A single i.v. injection of alloxan (75 mg/kg)	–	Chow diet	14	2.77			
	C57BL/6, male	NR	3 weeks	i.p. injection of STZ (55 mg/kg) for 5 d	–	High fat diet prior to mating	5	2.32	5.97		0.04
	C57BL/6, male	NR	6 weeks	A single i.p. injection of STZ (150 mg/kg)	–	Chow diet	3	1.64			
	129Sv, male	NR	8 weeks	A single i.v. injection of alloxan (75 mg/kg)	–	Chow diet	14	1.51			
	C57Bl/6J, male	NR	3 weeks	i.p. injection of STZ (55 mg/kg) for 5 d	–	Chow diet	19		2.16	0.15	−0.47
	C57Bl/6J, male	NR	3 weeks	A single i.p. injection of STZ (100 mg/kg)	–	High fat diet	49		0.63	7.79	0.4
	Kunming, male	NR	8 weeks	A single i.v. injection of STZ (150 mg/kg)	–	Chow diet	3			2.88	7.67
Chemical‐induced rat models	Wiatsr, male	NR	NR	A single i.p. injection of STZ (45 mg/kg)	–	Chow diet	14	49.89			
	Wistar, male	NR	8 weeks	A single i.p. injection of STZ (45 mg/kg)	–	Chow diet	7	48.72	6.42	14.15	10.78
	Wistar, male	NR	Adult	A single i.p. injection of alloxan (90 mg/kg)	–	Chow diet	14	29.9			
	SD, male	NR	7–8 weeks	A single i.p. injection of STZ (55 mg/kg)	–	Chow diet	2	18.99			
	Wistar, male	NR	NR	A single i.p. injection of STZ (65 mg/kg)	–	Chow diet	3	15.81			
	Wistar, male	NR	30 d	A single i.p. injection of STZ (60 mg/kg)	–	Chow diet	2	14.97			
	Wistar, male	NR	3 months	A single i.h. injection of alloxan (120 mg/kg)	–	Chow diet	2	14.12		7.15	7.54
	Wistar, male	NR	Adult	A single i.p. injection of STZ (55 mg/kg)	–	Chow diet	2	10.28			
	Wistar, male	NR	65–70 d	A single i.p. injection of STZ (60 mg/kg)	–	Chow diet	3	10.12		11.68	0.99
	Wistar, male	NR	Adult	A single i.p. injection of STZ (55 mg/kg)	–	Chow diet	3	8.46			
	SD, male	NR	NR	A single i.p. injection of STZ (50 mg/kg)	–	Chow diet	3	7.67			
	SD, NR	NR	NR	A single i.p. injection of STZ (50 mg/kg)	–	Chow diet	2	5.81			
	Wistar, male	NR	NR	A single i.p. injection of STZ (45 mg/kg)	–	High fat diet	21	5.16			
	SD, male	NR	8–10 weeks	Atropine, enrofloxacin, carprofen, saline	PX‐UNx	Chow diet	14	5			
	SD, male	NR	8 weeks	A single i.v. injection of STZ (55 mg/kg)	–	Chow diet	1	4.44			
	SD, male	NR	2 months	A single i.p. injection of STZ (30 mg/kg)	PX‐UNx	High glucose and High fat diet	42	3.93			
	Wistar, male	NR	NR	A single i.p. injection of STZ (55 mg/kg)	–	Chow diet	3	2.68			
	SD, male	NR	NR	A single i.v. injection of STZ (60 mg/kg)	–	Chow diet	3	2.53			
	SD, male	NR	NR	A single i.p. injection of STZ (60 mg/kg)	–	Chow diet	7	2.34		0.62	1.76
	Wistar, male	NR	NR	A single i.p. injection of STZ (50 mg/kg)	–	Chow diet	3	2.16		1.69	2.67
	Wistar, male	NR	8 weeks	NR	–	High glucose and High fat diet	105	1.7	0.82		
	SD, male	NR	6 weeks	A single i.p.injection of STZ (30 mg/kg)	–	High fat diet	59	1.48		1.86	1.94
	SD, male	NR	8 weeks	A single i.p injection of STZ (35 mg/kg)	–	High fat diet	31	0.4		0.19	0.44
	SD, male	NR	NR	A single i.p. injection of STZ (65 mg/kg)	–	Chow diet	3		7.3		
	SD, male	NR	NR	A single i.p. injection of STZ (65 mg/kg)	–	Chow diet	2		6.83	0.51	−1.16
	Wistar, male	NR	18 weeks	A single i.p. injection of STZ (65 mg/kg)	–	Chow diet	3		6.07	6.56	18.18
	SD, male	NR	NR	A single i.p. injection of STZ (60 mg/kg)	–	Chow diet	3		4.18		
	SD, male	NR	2 months	A single i.p. injection of STZ (30 mg/kg)	UNx	High glucose and High fat diet	42			4.69	3.11
	Wistar, male	NR	10 weeks	A single i.p. injection of STZ (65 mg/kg)	–	Chow diet	3			2.26	2.95
	SD, NR	NR	8 weeks	A single i.p. injection of STZ (60 mg/kg)	–	Chow diet	3			0.36	0.56
	SD, NR	NR	8 weeks	A single i.p. injection of STZ (60 mg/kg)	–	Chow diet	3			0.36	0.78
	SD, NR	NR	8 weeks	A single i.p. injection of STZ (60 mg/kg)	–	Chow diet	3			0.12	0.86

Abbreviations: BG, blood glucose, mean difference based on forest plot; HbA1c, glycosylated hemoglobin, mean difference based on forest plot; NR, not report; PX, pancreatectomy; STZ, streptozotocin; Modeling time(d), the length of the bar indicates the days of modeling; TC, total cholesterol, mean difference based on forest plot; TG, triglyceride, mean difference based on forest plot; UNx, unilateral nephrectomy.

Each cell's color in the table denotes a rise or fall in the data, with blue to red denoting a sequential rise.

Among genetic rat models included in the meta‐analysis, SDT rats showed the most significant increase in blood glucose levels, followed by Zucker obese rats. Because no standard deviations were reported, the LEA.PET‐pet congenic rats were not included in the forest plot, despite high blood glucose levels were observed in these rats as well. Nevertheless, no significant increase in HbA1c was found in these rat strains. In terms of lipid metabolism, there were marginal increases in TG and TC levels among these rat models, with SDT rats showing the most pronounced modifications, followed by Zucker obese rats, and finally LEA.PET‐pet congenic rats. Thus, only SDT rats showed an obvious increase in both glucose metabolism and lipid metabolism.

Genetic mouse models included in the meta‐analysis are BTBR^ob/ob^ mice, db/db mice, Akita^+/−^Ren^+/−^ mice, Ins2‐Akita mice, and KK‐A^y^ mice. All the above strains showed an increase in blood glucose, among which Akita mice showed the most significant change, followed by db/db mice, and finally BTBR^ob/ob^ mice. The most significant changes in HbA1c were founded in Akita^+/−^Ren^+/−^ mice, but no significant increase was found in other strains. Unlike blood glucose and HbA1c, TG and TC were only slightly increased in the above strains. These findings indicated that Akita^+/−^Ren^+/−^ mice developed the most severe metabolic syndrome, although other species also exhibited major symptoms of diabetes. Compared with rats, there are more transgenetic mouse diabetic models, showing more significant metabolic changes. Although the modeling period is long, the impact of human factors on modeling outcomes has been significantly reduced, resulting in a closer resemblance of the pathogenesis in these models to that of human DKD patients.

#### 3.3.2 Relationship between modeling methods and renal function in diabetic kidney disease

The primary characteristic of the transition from diabetes to diabetic kidney disease is the presence of albuminuria, accompanied by a decline in glomerular filtration rate (GFR). In the course of pathogenesis, there are also histopathological changes such as glomerular sclerosis, tubule interstitial fibrosis, and glomerular basement membrane (GBM) thickening. Serum creatine (SCR), blood urea nitrogen (BUN) and urinary albumin excretion rate (UAE) were usually selected as indicators of renal function and thus were included in the meta‐analysis to explore the relationship between different modeling methods and renal function in diabetic nephropathy. SCR, BUN, and UAE were used as reference indices for renal function, and subgroup analysis was carried out, respectively (Supporting Information Figures S6–S8).

As mentioned above, a single intraperitoneal injection of 45–60 mg/kg STZ in rats resulted in a significant phenotype of metabolic syndrome. As shown in Table 3, SCR, BUN and UAE were all significantly increased post STZ administration, indicating the degeneration of renal function and the presence of albuminuria. In contrast to SD rats, Wistar rats notably displayed more severe renal impairment. However, histopathological examination indicated that renal lesions caused by chemical induction, including dilatation and hyperplasia of the glomerular membrane and prominent nodular glomerulosclerosis with thickening of the glomerular basement membrane, were mild.^26^, ^27^, ^28^ Compared with rats, the changes in renal function in STZ‐treated mice were even less obvious. The administration regimen for intraperitoneal injection of STZ dominated the modeling effect. The graph illustrates that changes in lipid metabolism are also proportional to the dose and number of doses given. In addition, only minor renal lesions such as mesangial matrix expansion and basement membrane thickening were observed.^16^, ^29^, ^30^ As can be seen from Table 3, maternal obesity aggravated both diabetic milieus and renal dysfunction in the offspring, accompanied by renal fibrosis and renal oxidative stress, which may indicate a potential approach to establishing novel DKD models.^31^ These findings suggested that chemical‐induced models have a short modeling period and a simple methodology. Despite the significant alterations in renal function indexes are observed (as shown in Table 3), the renal histopathological changes in these models are relatively minor.

**TABLE 3:** The chart of the relationship between modeling conditions and renal function indices.

	Strain	Genotype	Age	Treatment	Surgery	Diet	Molding time(d)	SCR	BUN	UAE
Genetic mouse models	FVB/n‐C57BL/6, male	Akita^+/−^ Ren^+/−^	NR	NR	–	Chow diet	42	8.24		
	C57BLKS/J, male	ZnT8‐KOdb/db	NR	NR	–	Chow diet	56	4.05	0.37	
	C57BLKS/J, male	db/db	8 weeks	NR	–	Chow diet	56	3.43		
	C57BLKS/J, male	db/db	8 weeks	NR	–	Chow diet	56	3.2	10.9	
	C57BL/ 6 J, male	ZnT8‐KO‐STZ	NR	ZnT8‐KO intercross STZ‐induced(150 mg/kg)	–	Chow diet	56	2.68	1.23	
	Akita, male	Ins2 ^Akita/+^ Jak2^loxP/loxP^	NR	A single injection of Ang II (700 ng/min/kg)	–	Chow diet	70	2.13		
	FVB/NJ‐Akita, male	RTTA‐CD	4 weeks	500 mg/kg 5‐FC for 5 d	–	Chow diet	28	0.94		
	BTBR, NR	ob/ob	NR	NR	–	Chow diet	126	0.72	2.24	1.96
	C57BLKS/J, male	db/db	6 weeks	NR	–	Chow diet	42	0.65		
	C57BL/Ks, male	db/db	6 weeks	NR	–	Chow diet	56	0.57	1.74	
	Akita, male	Ins2	9 weeks	NR	–	Chow diet	63	−1.85		3.15
	BTBR, female	ob/ob	4–6 weeks	NR	–	High protein diet	98	−3.01		
	BTBR, female	ob/ob	4–6 weeks	NR	–	Chow diet	98	−3.91		
	C57BL/KS, male	db/db	6 weeks	NR	–	Chow diet	56			3.21
	OVE26, female	NR	2–3 months	NR	–	Chow diet	56			2.03
	OVE26, female	NR	2–3 months	NR	–	Chow diet	56			1.76
	C57BL/6J, male	eNOS^−/−^	8 weeks	i.p. injection of STZ (55 mg/kg) for 5 d	–	Chow diet	61			1.45
	C57BL/6, male	TauT^−/−^	7–8 weeks	i.p. injection of STZ (55 mg/kg) for 5 d	–	Chow diet	7		2.14	
	C57BL/6, NR	Nqo1 KO	NR	i.p. injection of STZ (50 mg/kg) for 5 d	–	Chow diet	3		1.94	
	C57BL/Ks, male	db/db	NR	NR	UNx	Chow diet	49		1.68	
	C57BL/6, male	TauT^−/−^	7–8 weeks	i.p. injection of STZ (55 mg/kg) for 5 d	–	Chow diet	7		1.54	
	C57BL/6, male	TauT^+/+^	7–8 weeks	i.p. injection of STZ (55 mg/kg) for 5 d	–	Chow diet	7		1.04	
	C57BL/6, male	TauT^+/−^	7–8 weeks	i.p. injection of STZ (55 mg/kg) for 5 d	–	Chow diet	7		1.03	
	C57BL/6, male	TauT^+/−^	7–8 weeks	i.p. injection of STZ (55 mg/kg) for 5 d	–	Chow diet	7		0.93	
	BTBR, male	ob/ob	NR	NR	–	Chow diet	70		0.85	
	C57BL/6, male	TauT^+/+^	7–8 weeks	i.p. injection of STZ (55 mg/kg) for 5 d	–	Chow diet	7		0.36	
Genetic rat models	Zucker Obese, male	NR	NR	NR	–	Chow diet	112	0.54		
	Ren2, NR	(mRen2)27	NR	A single i.p. injection of STZ (55 mg/kg)	–	Chow diet	3	−2.39		
	LEA.PET, male	NR	NR	NR	–	Chow diet	70	0	0.53	
	Zücker, male	NR	8 weeks	NR	–	Chow diet	98	−0.18		2.48
	Jcl:SDT, male	NR	NR	NR	–	High fructose diet	217			0.36
	Jcl:SDT, male	NR	NR	NR	–	Chow diet	189			−0.22
Chemical‐induced mouse models	C57BL/6, male	NR	10 weeks	i.p. injection of STZ (125 mg/kg) for 2 d	–	Chow diet	3	5.68	4.3	
	Kunming, male	NR	8 weeks	A single i.v. injection of STZ (150 mg/kg)	–	Chow diet	3	4.41	3.07	
	C57BL/6, male	NR	7 weeks	A single i.p. injection of STZ (100 mg/kg)	UNx	High fat diet	24	3.56		
	C57BL/6J, male	NR	6 weeks	A single i.p. injection of STZ (150 mg/kg)	–	Chow diet	3	2.77		
	C57BL/6, male	NR	3 weeks	i.p. injection of STZ (55 mg/kg) for 5 d	–	Chow diet prior to mating	5	1.75		1.13
	C57BL/6, male	NR	6–7 weeks	A single i.p. injection of STZ (180 mg/kg)	–	Chow diet	3	1.14	1.44	
	C57BL/6, male	NR	3 weeks	i.p. injection of STZ (55 mg/kg) for 5 d	–	High fat diet prior to mating	5	1.11		1.58
	C57BL/6, male	NR	NR	i.p. injection of STZ (55 mg/kg) for 5 d	–	Chow diet	8	0.64		17.51
	C57BL/6J, NR	NR	NR	A single i.p.injection of STZ (100 mg/kg)	–	Chow diet	7	0.39		4.09
	C57BL6/J, male	NR	5 weeks	i.p. injection of STZ (50 mg/kg) for 5 d	UNx	Chow diet	11	0.19		
	C57BL6/J, male	NR	5 weeks	i.p. injection of STZ (50 mg/kg) for 5 d	UNx	Western diet	11	0.1		
	DBA/2J, male	NR	10 weeks	i.p. injection of STZ (40 mg/kg) for 5 d	–	Chow diet	26			0.74
	DBA/2J, male	NR	10 weeks	i.p. injection of STZ (40 mg/kg) for 5 d	–	Chow diet	26			0.59
	C57BL/6, NR	NR	NR	i.p. injection of STZ (50 mg/kg) for 5 d	–	Chow diet	3		2.19	
	C57BL/6J, male	NR	6 weeks	A single i.p. injection of STZ (150 mg/kg)	–	Chow diet	3		1.59	
Chemical‐induced rat models	Wistar, male	NR	8 weeks	A single i.p. injection of STZ (45 mg/kg)	–	Chow diet	7	17.72	15.3	9.41
	Wistar, male	NR	65–70 d	A single i.p. injection of STZ (60 mg/kg)	–	Chow diet	3	17.42		
	Wistar, male	NR	NR	i.p. STZ (65 mg/kg) + i.p. Nicotinamide (110 mg/kg)	–	Chow diet	3	15.51		
	Wistar, male	NR	Adult	A single i.p. injection of STZ (55 mg/kg)	–	Chow diet	2	12.42	11.58	
	Wistar, male	NR	adult	A single i.p. injection of alloxan (90 mg/kg)	–	Chow diet	14	10.03		
	Wistar, male	NR	Adult	A single i.v. injection of STZ (60 mg/kg)	–	Chow diet	2	9.67		5.28
	SD, male	NR	NR	A single i.p. injection of STZ (50 mg/kg)	–	Chow diet	3	8.78		
	Wistar, male	NR	NR	A single i.p. injection of STZ (50 mg/kg)	–	Chow diet	3	8.64		
	Wistar, male	NR	Adult	A single i.v. injection of STZ (65 mg/kg)	UNx	Chow diet	22	8.56		
	SD, male	NR	NR	A single i.p. injection of STZ (35 mg/kg)	–	High fat diet	49	8.18	2.49	
	Wistar, male	NR	NR	A single i.p. injection of STZ (45 mg/kg)	–	Chow diet	2	7.3	15.86	
	SD, male	NR	6 weeks	A single i.p.injection of STZ (30 mg/kg)	–	High fat diet	59	7.08	1.55	
	SD, male	NR	2 months	A single i.p. injection of STZ (30 mg/kg)	–	High glucose and High fat diet	42	6.73	3	
	SD, male	NR	2 months	A single i.p. injection of STZ (30 mg/kg)	–	High glucose and High fat diet	42	3.91	2.83	
	SD, male	NR	7–8 weeks	A single i.p. injection of STZ (55 mg/kg)	–	Chow diet	2	3.6	5.76	
	Wistar, male	NR	NR	A single i.p. injection of STZ (45 mg/kg)	–	High fat diet	21	3.38	3.22	
	Wistar, male	NR	3 months	A i.h. injection of alloxan (120 mg/kg)	–	Chow diet	2	2.95		
	Wistar, male	NR	NR	A single i.p. injection of STZ (45 mg/kg)	–	Chow diet	3	2.86	15.22	

	Wistar, male	NR	12 weeks	A single i.v. injection of STZ (40 mg/kg)	USO	Chow diet	49	2.79		
	Wistar, male	NR	Adult	A single i.p. injection of STZ (55 mg/kg)	–	Chow diet	3	2.77	3.59	
	SD, male	NR	NR	A single i.p. injection of STZ (50 mg/kg)	–	Chow diet	3	2.44	2.59	
	Wistar, male	NR	Adult	A single i.v. injection of STZ (65 mg/kg)	UNx	Chow diet	2	2.34		
	SD, male	NR	NR	A single i.p. injection of STZ (65 mg/kg)	–	Chow diet	2	1.75		
	SD, male	NR	NR	A single i.p. injection of STZ (65 mg/kg)	–	Chow diet	3	1.68		1.19
	SD, NR	NR	8 weeks	A single i.p. injection of STZ (60 mg/kg)	–	Chow diet	3	1.21	1.4	1.47
	SD, NR	NR	8 weeks	A single i.p. injection of STZ (60 mg/kg)	–	Chow diet	3	0.81	0.99	0.55
	SD, NR	NR	8 weeks	A single i.p. injection of STZ (60 mg/kg)	–	Chow diet	3	0.59	0.8	4.71
	Wistar, male	NR	10 weeks	i.p. STZ (65 mg/kg) + i.p. Nicotinamide (110 mg/kg)	–	Chow diet	3	0.5		
	Wistar, male	NR	8 weeks	NR	–	High glucose and High fat diet	105	0.4	−3.18	
	SD, male	NR	8–10 weeks	Atropine, enrofloxacin, carprofen, saline	PX‐UNx	Chow diet	14	−1.02		
	SD, male	NR	6 weeks	A single i.p. injection of STZ (35 mg/kg)	–	High glucose and High fat diet	63	−1.26		
	SD, male	NR	7 weeks	A single i.p. injection of STZ (150 mg/kg)	–	Chow diet	7			10.72
	Wistar, male	NR	6–7 weeks	A single i.v. injection of STZ (55 mg/kg)	–	Chow diet	5			6.67
	Wistar, male	NR	NR	A single i.p. injection of STZ (65 mg/kg)	–	Chow diet	3		6.79	6.18
	Wistar, male	NR	NR	A single i.p. injection of STZ (55 mg/kg)	–	Chow diet	3			3.68
	SD, male	NR	NR	A single i.v. injection of STZ (60 mg/kg)	–	Chow diet	3			1.01
SD, male	NR	8 weeks	A single i.p. injection of STZ (35 mg/kg)	–	High fat diet	31		0.59	0.76

Abbreviations: BUN, blood urea nitrogen, mean difference based on forest plot; SCR, serum creatine, mean difference based on forest plot; UAE, urinary albumin excretion rate, mean difference based on forest plot; USO, unilateral salpingo‐oophorectomy.

Each cell's color in the table denotes a rise or fall in the data, with blue to red denoting a sequential rise.

As shown in Table 3, among genetic mouse models, an increase in renal function index from high to low was seen in the order of Akita^+/−^Ren^+/−^ mice, ZnT8‐KO‐db/db mice, db/db mice, BTBR^ob/ob^ mice, Ins2‐Akita mice, OVE26 mice. Specifically, Akita^+/−^Ren^+/−^ mice showed a significant increase in both SCR and UAE, indicating the onset of renal degeneration, accompanied by glomerular injury, tubule interstitial fibrosis, collagen deposition and other advanced diabetic nephropathy.^25^ Significant changes in both glucose metabolism and renal function were also found in db/db mice, which showed mild glomerular hypertrophy with endothelial hyperplasia at an early stage, and moderate albuminuria and severe glomerular hypertrophy when they aged, but with only minor tubulointerstitial changes.^16^, ^32^ When ZnT8 was further knocked out in db/db mice, SCR was then significantly elevated and renal tubulointerstitial fibrosis was observed.^16^ Although BTBR^ob/ob^ mice presented general changes in diabetic metabolism and renal function, they showed extensive glomerular matrix dilatation, focal nodular glomerular sclerosis, mild basement membrane thickening, interstitial fibrosis and other advanced DKD features in the late stage.^33^, ^34^, ^35^ Given the significant changes in diabetes metabolism and renal function observed in BTBR^ob/ob^ mice, as well as the presence of many major lesions of advanced DKD, this model is highly suitable for pathogenesis research. However, not all diabetic mice showed changes in renal function index. For instance, no significant alterations in renal function indicators were seen in Ins2‐Akita mice, although mesangial matrix increased and lesions on the basement membrane thickened.^36^ The renal function indicators of OVE26 mice were also not significantly altered, yet a number of identifiable diabetic nephropathy lesions, including glomerular hypertrophy and glomerular interstitial fibrosis were observed.^37^

Among the genetic rats, only the ZDF rat showed a significant elevation in UAE and developed albuminuria. Zucker obese rats showed not only elevated levels of diabetic metabolism, but also renal degeneration. With the progression of the disease, rats accumulate a large amount of cholesterol and fatty acids, and lipid peroxidation occurs in the body, resulting in cytotoxicity, kidney cell apoptosis and organ damage. Notably, there were no indications of renal degeneration observed in SDT rats and LEA.PET‐pet congenic rats.^38^

Although the DKD animal model established by the chemical induction method has a short modeling time and a high modeling rate, the renal lesions are mild. Compared with chemical induction, genetic animals, including those mice not included in the meta‐analysis, are more likely to develop diabetic nephropathy. Specifically, Ins2‐Akita mice have more severe renal lesions. db/db mice exhibit glomerular lesions early on, but tubulointerstitial lesions are less severe.^25^ KKA^y^ mice have glomerular basement membrane thickening and diffuse mesangial dilatation, but are prone to renal hydronephrosis, diabetic ketoacidosis, and high mortality.^39^ These mice have mild renal lesions and can be used to simulate early DKD. The following models show several essential traits of end‐stage kidney damage and can imitate advanced human DKD. eNOS^−/−^ and BTBR^ob/ob^ mice can are capable of developing late stage functional and morphological changes associated with DKD, but are expensive and difficult to breed.^34^, ^40^, ^41^ (mRen2)27 transgenic rats^24^ and OVE26‐TTRhRen transgenic mice^23^ show more striking manifestations of advanced DKD in a shorter timeframe, due to the combined effect of renin‐dependent hypertension and diabetes. As such, these models hold significant promise for developing more accurate and efficient DKD models.

#### 3.3.3 Limitations of the above models and potential remedial methods

The above analysis shows that the occurrence of metabolic syndrome, the changes in renal function indexes, and the development of late DKD lesions cannot be observed in a single model at the same time. Thus, in order to overcome these defects, various attempts have been made to modify basic models or to combine multiple stimuli.

The first choice is to optimize the administration regimen of STZ. The selection of STZ administration regimen mainly depends on the type and severity of DM to be simulated. This study breaks down the STZ injectable dose and delivery times into four categories: multiple high‐dose injection, single high‐dose injection, multiple low‐dose injection, and single low‐dose injection. Multiple high‐dose STZ administrations can cause the death of pancreas β cells and the release of lots of insulin, leading to short‐term hypoglycemic mortality. Because of the low modeling rate, it has not been frequently utilized. Both single high‐dose administration and multiple low‐dose administrations of STZ are able to induce type 1 DM‐like phenotypes. A single high‐dose injection of STZ can harm pancreatic β cells, resulting in a type 1 DM model with absolute insulin deficiency. Multiple low‐dose administrations of STZ also establish stable and lasting type 1 DM through persistent stimulation of the autoimmune response and destruction of the immune mechanism. Therefore, multiple low‐dose administrations of STZ can better imitate the pathogenesis of type 1 DM and reduce mortality than single high‐dose administration of STZ, but the modeling cycle is longer.

Administering a single high‐dose of STZ along with niacinamide (NA) has been shown to replicate key features of type 2 DM, but not type 1 DM. NA is an amide form of vitamin B3 that promotes intracellular DNA repair, maintains the level of niacinamide adenine dinucleotide, and thus protects pancreatic β cells from complete destruction after STZ treatment.^42^ Type 2 DM can also be induced by a single low‐dose administration of STZ in combination with a high‐fat diet. The ideal type 2 DM model should exhibit traits such as insulin resistance, abnormal glucose and lipid metabolism, and relative insulin deficiency, but it cannot be established solely by a single low‐dose administration of STZ, since only a minor fraction of islet β cells are destroyed by a single low‐dose administration of STZ, which does not affect insulin resistance.^43^ Providing an exclusive high‐fat diet can cause insulin resistance to develop over time. At this stage, administering a low dose of STZ can help destroy some of the islet β cells, which can eventually lead to the loss of compensatory abilities in the body. This approach can effectively establish a type 2 DM model over a shorter timeframe.^44^, ^45^, ^46^ It is worth noting that the STZ dosage was affected by the duration of the high‐sugar and high‐fat diet feeding schedule. Insulin resistance became more severe with prolonged feeding, increasing the load of insulin secretion and reducing the required dose of STZ. In addition, to ensure optimal results, the solution used for STZ administration should meet specific requirements for sodium citrate concentration and pH value. The recommended pH value ranges between 4.2 and 4.5, while the concentration of sodium citrate should fall between 0.1 and 0.2 mol/L. Using a water‐based solution under these conditions results in the most stable solution and yields the best efficacy and modeling impact for STZ.

All of the aforementioned STZ administration regimens can generate qualified type 1 and type 2 DM models. However, due to the mild renal lesions, these models cannot be directly used as suitable DKD models. To achieve greater similarity between nephropathies in DM models and human DKD, a modified high‐sugar diet and unilateral nephrectomy (UNx) can be used to exacerbate pathological changes in the kidney. As previously mentioned, a high‐sugar diet can increase vascular permeability and cause albuminuria by increasing the reactivity of vasoactive factors.^9^ It can also promote the production of TNF‐β in the kidney, which leads to the deposition of extracellular collagen, mesangial expansion, GBM thickening, and other renal pathological injuries.^10^ The addition of sugar to the diet can directly increase the rats' blood glucose values, improve the palatability of the diet, and increase food intake, which significantly accelerates the modeling process of DKD. Unlike a high‐sugar diet, which exacerbates renal lesions through a separate mechanism, UNx increases the burden on the remaining kidney by removing one kidney, allowing the key lesions of advanced DKD in humans, such as renal tubule interstitial fibrosis, glomerular basement membrane thickening, and mesangial dilation, to be seen in a relatively short period of time.^44^, ^47^, ^48^ For better reproduction of DKD features in DM mice, UNx can be performed inaddition to STZ injection combined with a high‐fat and high‐sugar diet. This approach is commonly referred to as the triple method and produces significant biochemical and physiological changes in a relatively short timeframe. However, it has some disadvantages, including complex operation, susceptibility to infection, and high mortality rates.^29^, ^48^, ^49^

In genetic mouse models, additional diet changes and chemical induction are common approaches used to exacerbate DKD progression. STZ remains the most commonly used reagent for this purpose. eNOS^−/−^ mice and TTRhRen renin‐transgenic mice exhibit significant renal function decline and renal lesions, and STZ administration can further aggravate diabetic metabolism.^23^, ^41^ It has also been shown that STZ‐treated C57BL/6 mice following TauT gene knockout displayed more advanced diabetic nephropathy than STZ‐induced C57BL/6 mice, as evidenced by considerable GBM thickening, enlarged mesangial matrix, fibrous thickening of efferent and afferent arterioles with severe dilatation and arteriosclerosis, and major renal hypertrophy. These findings suggest that removing the TauT gene makes C57BL/6 mice more susceptible to STZ‐induced diabetic nephropathy and provides a promising basis for developing a novel DKD model.^50^ Several other stimuli have also been tested for use in combination to promote kidney injuries in DKD models. In this study BTBR^ob/ob^ mice fed a high‐protein diet showed increased mesangial dilatation and signs of interstitial fibrosis, and pro‐inflammatory and pro‐oxidative signaling pathways were activated both systemically and locally in the kidneys.^33^ Another study showed that when Ins2‐Akita mice were given a single intraperitoneal injection of Ang II, JAK–STAT mRNA and protein expression rose, first in the glomeruli, and then gradually in the renal tubulointerstitium, which fits with the natural development of DKD. These data show that increasing JAK2‐specific expression in podocytes may worsen DKD severity.^51^ In the course of DKD development, aggravated podocyte injury has been shown to encourage the emergency of noticeable mesangial enlargement, interstitial fibrosis, increased GBM thickness and robust albuminuria. The author of this study gave 500 mg/kg 5‐FC to Akita‐CD mice for 5 days and discovered that podocyte damage was exacerbated, promoting the advancement of DKD.^52^ Recently, a potential new model for inflamed DKD has been established by administering 0.5 mL of 10% casein to db/db mice daily.^53^

## 4 DISCUSSION

At present, all models have simulated human diabetic kidney disease to some extent, but none have successfully simulated and replicated all features of human diabetic kidney disease, particularly with regard to renal dysfunction and nephropathy. Therefore, there is an urgent need for a model that demonstrates significant changes in all three aspects of the disease as it progresses. Such a model would allow for a more complete understanding of the pathogenesis and treatment of DKD. Generally speaking, chemical‐induced animal models are relatively inexpensive, easy to operate, and have a high modeling success rate. Spontaneous DKD animal models reproduce human factors to a certain extent and are closer to the natural process of human diabetic kidney disease. Nonetheless, obtaining these models can be difficult due to their limited availability, requirements for particular breeding and feeding conditions, low incidence rate, lengthy modeling cycle, and potential cost. The combination of pathogenic genes and chemical stimuli can quickly establish DKD models, some of which exhibit the basic characteristics of human DKD, and this may provide the development direction of DKD animal models in the future. Compared to the genetic mouse models, genetic rats provide a more comprehensive representation of the natural chronic progression of DKD. However, the associated expenses of developing and sustaining these rats has proved to be challenging and the high time and cost considerations of genetic manipulation in rats make it is almost impossible to study the impact of additional specific genes on DKD. Consequently, developing a DKD model using mice may prove to be a more practical solution.

## 5 CONCLUSION

In this study, 84 literature reports related to DKD that met initial requirements were included in a meta‐analysis to analyze the relationship between different modeling methods and diabetic metabolism and renal function. Based on the analysis results and actual modeling requirements, such as price, operability, mortality and modeling rate, the animal model induced by a high energy diet combined with STZ and UNx represents the ideal DKD model at present. In conclusion, despite the limitations of current DKD animal models in replicating the full spectrum of human DKD, they have still proven invaluable in advancing our understanding of the progression of this complex disease. In the future, addressing the remaining issues in these models will be crucial in achieving an even more accurate replication of human DKD and furthering our ability to develop effective treatments.

## AUTHOR CONTRIBUTIONS

Li Fanghong: Investigation, Formal analysis, Writing‐Original Draft. Ma Zhi: Modification, Methodology. Cai Yajie: Software, Data Curation. Zhou jingwei: Conceptualization, Methodology, Funding acquisition. Liu Runping: Conceptualization, Methodology, Writing‐Review & Editing.

## CONFLICT OF INTEREST STATEMENT

Runping Liu is an Editorial Board member of *Animal Models and Experimental Medicine* and a co‐author of this article. To minimize bias, he was excluded from all editorial decision‐making related to the acceptance of this article for publication.

## ETHICS STATEMENT

This article did not involve human or animal experimentation. There are no ethical issues and other conflicts of interest.

## Supporting information


Supplementary Figure 1.
