# Supplementary material for: Optimizing diabetic kidney disease animal models: Insights from a meta‐analytic approach
Source: Animal Model Exp Med. 2023 Sep 18;6(5):433–51. doi: 10.1002/ame2.12350 (PMC10614131; doi:10.1002/ame2.12350)
Supplement: Supplementary file 1 — Supplementary Figure 1. [file AME2-6-433-s001.pdf]

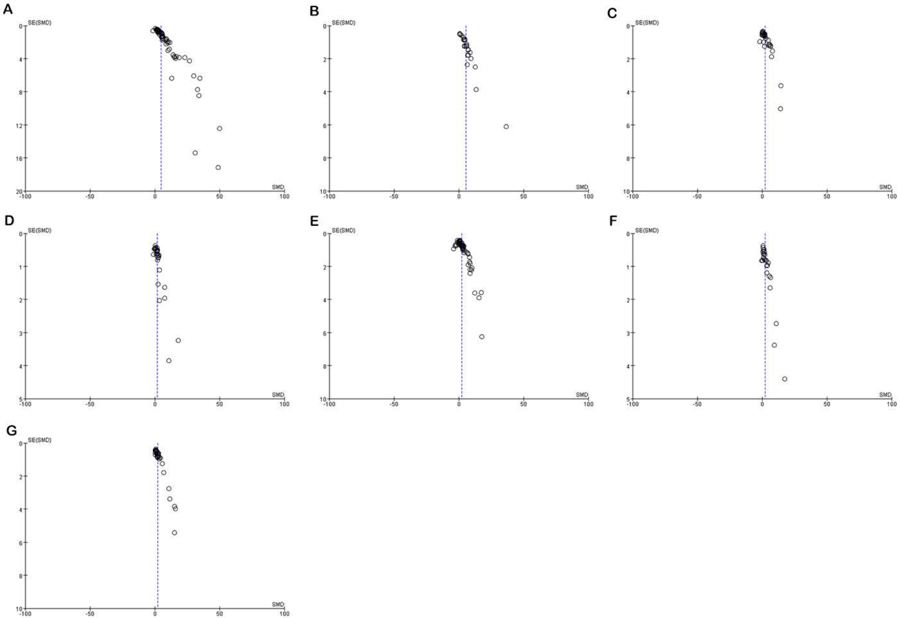

Supplementary Figure. 1. Public bias of **(A)** Blood glucose, **(B)** HbA1c, **(C)** TC, **(D)** TG, **(E)** SCR, **(F)** UAE, **(G)** BUN on DKD.

Study or Subgroup	Model Mean	Model SD	Model Total	Control Mean	Control SD	Control Total	Weight	Std. Mean Difference IV, Random, 95% CI
**2.1.1 Genetic mouse models**								
Morigi, M., et al. (2020)-14wk old	596	11.31	8	135	15.65	5	0.1%	32.85 [17.68, 48.01]
Wang, X. X., et al. (2017)-type1-22wk old	510	13.61	12	143	12.71	12	0.4%	26.91 [18.54, 35.28]
He, X., et al. (2019)-26wk old	543.6	25.2	6	163.8	10.8	16	0.5%	23.28 [15.63, 30.94]
Itano, S., et al. (2020)-13wk old	721.8	39.68	6	237.6	13.23	6	0.5%	15.11 [7.65, 22.58]
Ma, K. L., et al. (2014)-16 wk old	836.46	70.2	10	231.84	31.14	10	1.1%	10.66 [6.87, 14.45]
Li, H. Y., et al. (2017)-16 wk old	591.4	53.13	10	142.2	39.84	10	1.3%	9.16 [5.87, 12.45]
Cassis, P., et al. (2019)-19/20 wk old	540	84	9	138	18	9	1.5%	6.30 [3.80, 8.81]
Liu, H. W., et al. (2019)-13wk old	551	104.65	8	85	11.31	8	1.5%	5.92 [3.36, 8.48]
Somineni, H. K., et al. (2014)-17wk old	613.8	101.41	6	167.4	17.64	6	1.4%	5.66 [2.68, 8.65]
Wang, X. X., et al. (2017)-type2-22wk old	376	66.16	12	116	23.56	12	1.8%	5.06 [3.30, 6.81]
Chodavarapu, H., et al. (2013)-14wk old	677.6	179.06	6	172.5	12.28	6	1.7%	3.67 [1.55, 5.79]
Björnson Granqvist, A., et al. (2020)-HPD-20wk old	627.3	186.12	9	167.22	14.76	7	1.8%	3.08 [1.51, 4.66]
Arimura, E., et al. (2018)-H diet-11 week old	923	308.64	6	225	41.64	6	1.7%	2.93 [1.10, 4.75]
Klein, J., et al. (2016)-20 wk old	437.4	151.2	9	163.8	10.8	9	1.9%	2.43 [1.14, 3.72]
Li, M., et al. (2013)-16wk old	502.38	249.98	8	66.96	31.06	8	1.9%	2.31 [0.97, 3.66]
Klein, J., et al. (2016)-22 wk old	599.4	280.59	11	102.6	27	9	1.9%	2.27 [1.09, 3.45]
Pichaiwong, W., et al. (2013)-24wk old	503.4	190.81	6	161	56.13	5	1.8%	2.13 [0.50, 3.75]
Björnson Granqvist, A., et al. (2020)-20wk old	428.94	171	9	167.22	14.76	7	1.9%	1.91 [0.66, 3.16]
Zhang, Y., et al. (2018)-12wk old	309.6	157.83	8	95.4	25.46	8	1.9%	1.79 [0.58, 3.00]
Li, M., et al. (2013)-12 wk old	326.52	422.06	8	35.28	23.42	8	2.0%	0.92 [-0.13, 1.97]
**Subtotal (95% CI)**			**167**			**167**	**28.6%**	**5.05 [3.77, 6.33]**
Heterogeneity: Tau² = 6.25; Chi² = 158.87, df = 19 (P < 0.00001); I² = 88%								
Test for overall effect: Z = 7.72 (P < 0.00001)								
**2.1.2 Genetic rat models**								
Toyoda, K., et al. (2018)-31wk old	762	19	3	203	7	3	0.0%	31.23 [1.03, 61.44]
Toyoda, K., et al. (2018)-HFD-31wk old	934	64	3	203	7	3	0.2%	12.85 [0.34, 25.35]
Lezcano, E. J., et al. (2014)-19 wk old	593	63	8	188	78	5	1.4%	5.47 [2.72, 8.23]
**Subtotal (95% CI)**			**14**			**11**	**1.7%**	**9.90 [0.45, 19.34]**
Heterogeneity: Tau² = 35.98; Chi² = 3.97, df = 2 (P = 0.14); I² = 50%								
Test for overall effect: Z = 2.05 (P = 0.04)								
**2.1.3 Chemical-induced mouse models**								
Lopez-Parra, V., et al. (2012)-STZ-15wk	512	12	12	137	6	7	0.2%	34.81 [22.34, 47.28]
Tung, C. W., et al. (2019)-8wk DM	439.4	3	6	140	11.1	6	0.1%	33.99 [17.36, 50.62]
Yang, S., et al. (2016)-2wk DM	505.575	28.32	6	122.1	33.86	6	0.7%	11.34 [5.69, 16.99]
Lian, H., et al. (2019)-4wk DM	348.66	30.78	6	113.94	17.64	6	1.0%	8.64 [4.27, 13.00]
Mohamed, R., et al. (2013)-12wk DM	682	98.99	8	217	31.11	8	1.5%	5.99 [3.41, 8.58]
Franzén, S., et al. (2014)-C57Bl/6 -5wk DM	381.6	56.12	12	129.6	18.71	12	1.7%	5.82 [3.85, 7.79]
Dusabimana, T., et al. (2020)-6wk-STZ	316	59.78	10	104.375	26.98	8	1.8%	4.18 [2.37, 5.98]
Franzén, S., et al. (2015)- BALB/c-10wk DM	406.8	87.3	12	115.2	43.65	12	1.8%	4.08 [2.58, 5.57]
Glastras, S. J., et al. (2016)-lean-32wk old	400.14	52.38	9	253.98	24.84	9	1.8%	3.40 [1.84, 4.95]
Franzén, S., et al. (2015)- BALB/c-5wk DM	390.6	106	12	127.8	31.18	12	1.9%	3.25 [1.96, 4.53]
Franzén, S., et al. (2017)-129Sv-5wk DM	372.6	118.47	12	108	18.71	12	1.9%	3.01 [1.78, 4.24]
Franzén, S., et al. (2014)-C57Bl/6 -10wk DM	430.2	149.65	12	118.8	24.94	12	1.9%	2.80 [1.62, 3.98]
Franzén, S., et al. (2015)-NMRI-10wk DM	505.8	193.3	12	109.8	24.94	12	1.9%	2.77 [1.60, 3.95]
Franzén, S., et al. (2015)-NMRI-5wk DM	462.6	168.36	12	115.2	31.18	12	1.9%	2.77 [1.60, 3.94]
Glastras, S. J., et al. (2016)-obese-32wk old	365.04	59.4	9	253.98	24.84	9	1.9%	2.32 [1.06, 3.58]
Jeong, B. Y., et al. (2016)-STZ 16wk	427	177.27	7	204	31.75	7	1.9%	1.64 [0.37, 2.91]
Franzén, S., et al. (2017)-129Sv-10wk DM	379.8	230.71	12	120.6	37.41	12	2.0%	1.51 [0.59, 2.44]
**Subtotal (95% CI)**			**169**			**162**	**26.1%**	**3.83 [2.89, 4.78]**
Heterogeneity: Tau² = 2.66; Chi² = 85.91, df = 16 (P < 0.00001); I² = 81%								
Test for overall effect: Z = 7.95 (P < 0.00001)								
**2.1.4 Chemical-induced rat models**								
Ibrahim, D. S. (2015)-30Day DM	264.67	1.53	6	118.33	3.51	6	0.1%	49.89 [25.51, 74.27]
Shiju, T. M., et al. (2013)-STZ 12wk	582	12	4	100	2	4	0.0%	48.72 [15.18, 82.26]
Elseweidy, M. M., et al. (2013)-8wk DM	480	13.2	8	94.3	11.1	8	0.2%	29.90 [17.93, 41.88]
Garud, M. S. and Y. A. Kulkarni (2018)-STZ 8wk	753.3	35.64	8	139.68	24.44	8	0.5%	18.99 [11.35, 26.63]
Liu, W., et al. (2013)-16wk DM	601.02	30.78	6	140.58	22.32	6	0.5%	15.81 [8.01, 23.61]
Ladeira, L. C. M., et al. (2021)-6wk DM	475	33.14	6	85.38	7.53	6	0.5%	14.97 [7.57, 22.36]
Gargouri, M., et al. (2018)-3wk DM	525.4	36.74	6	113.4	10.04	6	0.5%	14.12 [7.14, 21.11]
Sadar, S., et al. (2016)-STZ 4wk	261.2	10.96	5	115.8	14.36	5	0.7%	10.28 [4.36, 16.20]
Ibrahim, Z. S., et al. (2016)-8wk DM	479.3	50	8	101	0.7	8	1.0%	10.12 [5.96, 14.27]
Goru, S. K., et al. (2017)-STZ 8wk	468	50.91	8	108	25.46	8	1.2%	8.46 [4.94, 11.97]
Alzahrani, S., et al. (2020)-8wk DM	170.47	19.65	6	45.94	7.94	6	1.1%	7.67 [3.76, 11.58]
Christensen, M., et al. (2019)-STZ 4wk	509.4	92.6	6	90	17.64	6	1.3%	5.81 [2.76, 8.86]
Amin, S. N., et al. (2020)-6wk DM	270.533	42.6	6	94.233	13.35	6	1.4%	5.16 [2.40, 7.91]
Thisted, L., et al. (2020)-px unx 8wk	471.6	107.46	11	86.4	6.24	12	1.8%	5.00 [3.21, 6.78]
Nordquist, L., et al. (2015)-4wk DM	482.4	106	12	108	10.8	9	1.8%	4.44 [2.72, 6.16]
Hu, S. J., et al. (2018)-STZ 1wk	407.52	105.12	10	110.16	23.94	12	1.8%	3.93 [2.40, 5.47]
Alomari, G., et al. (2020)-drug 7wk	340.83	111.41	7	113.1	14.82	7	1.8%	2.68 [1.11, 4.25]
Xin, R., et al. (2018)-4wk DM	509.4	216	9	100.8	27	9	1.9%	2.53 [1.21, 3.84]
Ding, D., et al. (2016)-STZ 14wk	568.8	216.81	15	140.4	128.27	15	2.0%	2.34 [1.38, 3.30]
Ali, M. A. M., et al. (2017)-STZ 60day	464	197.99	8	142	22.63	8	1.9%	2.16 [0.86, 3.47]
Nunes, S., et al. (2020)-23wk old	273.14	94.84	8	149.14	22.49	8	1.9%	1.70 [0.51, 2.89]
Chen, Y., et al. (2016)-8wk DM	480.6	318	12	113.4	51.23	10	2.0%	1.48 [0.51, 2.45]
Dong, D., et al. (2019)-STZ 8wk	691.02	104.57	15	291.78	1,377.34	15	2.0%	0.40 [-0.33, 1.12]
**Subtotal (95% CI)**			**190**			**188**	**28.0%**	**5.50 [4.21, 6.80]**
Heterogeneity: Tau² = 6.34; Chi² = 192.38, df = 22 (P < 0.00001); I² = 89%								
Test for overall effect: Z = 8.32 (P < 0.00001)								
**2.1.5 Genetic mouse models with treatment**								
Huang, C., et al. (2018)-38 week old	467.28	22.24	7	188.28	0.63	7	0.5%	16.60 [9.27, 23.93]
Ma, K. L., et al. (2014)-inflame-16wk	943.56	77.4	10	231.84	31.14	10	1.0%	11.55 [7.46, 15.65]
Thibodeau, J. F., et al. (2014)-TTRhRen-18wk old	561.6	76.5	5	189	32.22	5	1.2%	5.73 [2.28, 9.19]
Dschietzig, T. B., et al. (2015)-12wk DM	523	173.21	12	145	64.9	13	1.9%	2.84 [1.68, 4.00]
Han, X., et al. (2015)-5mo DM	369	107.52	10	128	47.43	10	1.9%	2.78 [1.48, 4.08]
Han, X., et al. (2015)-3mo DM	360	183.41	10	119	47.43	10	2.0%	1.72 [0.66, 2.78]
Han, X., et al. (2015) TauT-/- 5mo DM	396	189.74	10	136	101.19	10	2.0%	1.64 [0.59, 2.68]
Han, X., et al. (2015)-TauT+/- 5mo DM	380	177.09	10	139	113.84	10	2.0%	1.55 [0.52, 2.58]
Han, X., et al. (2015)-TauT-/- 3mo DM	385	332.04	10	125	110.68	10	2.0%	1.01 [0.06, 1.95]
**Subtotal (95% CI)**			**84**			**85**	**14.5%**	**3.07 [1.85, 4.29]**
Heterogeneity: Tau² = 2.42; Chi² = 49.78, df = 8 (P < 0.00001); I² = 84%								
Test for overall effect: Z = 4.94 (P < 0.00001)								
**2.1.6 Genetic rat models with treatment**								
te Riet, L., et al. (2014)-5wk DM	441	40.73	8	108	25.46	8	1.1%	9.27 [5.44, 13.10]
**Subtotal (95% CI)**			**8**			**8**	**1.1%**	**9.27 [5.44, 13.10]**
Heterogeneity: Not applicable								
Test for overall effect: Z = 4.75 (P < 0.00001)								
**Total (95% CI)**			**632**			**621**	**100.0%**	**4.57 [3.97, 5.16]**
Heterogeneity: Tau² = 4.39; Chi² = 522.06, df = 72 (P < 0.00001); I² = 86%								
Test for overall effect: Z = 15.08 (P < 0.00001)								
Test for subgroup differences: Chi² = 17.23, df = 5 (P = 0.004), I² = 71.0%								

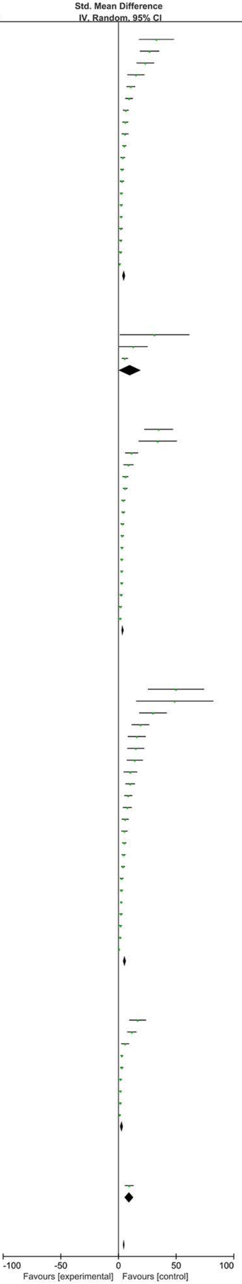

Supplementary Figure.2. Forest plot of blood glucose

Study or Subgroup	Model Mean	Model SD	Model Total	Control Mean	Control SD	Control Total	Weight	Std. Mean Difference IV, Random, 95% CI
**3.1.1 Genetic mouse models**								
He, X., et al. (2019)-26wk old	13.4	0.4	6	5.1	0.1	16	0.8%	36.64 [24.65, 48.62]
Terami, N., et al. (2014)-20wk old	9.2	0.45	5	4	0.22	5	1.7%	13.26 [5.69, 20.83]
Björnson Granqvist, A., et al. (2020)-HPD-20wk old	12.35	0.98	9	4.81	0.18	7	3.5%	9.50 [5.58, 13.42]
Klein, J., et al. (2016)-22 wk old	13.3	1.33	11	4	0.3	9	4.0%	8.81 [5.64, 11.98]
Klein, J., et al. (2016)-20 wk old	11.5	1.2	9	4.7	0.3	9	4.2%	7.40 [4.52, 10.29]
Zhang, Y., et al. (2018)-12wk old	9.3	1.24	8	4.07	0.198	8	4.5%	5.57 [3.14, 8.00]
Li, H. Y., et al. (2017)-16 wk old	11.1	1.9	10	4.5	0.32	10	4.9%	4.64 [2.81, 6.47]
Wang, X. X., et al. (2017)-type1-22wk old	9.6	1.63	12	4	0.35	12	5.0%	4.59 [2.96, 6.22]
Itano, S., et al. (2020)-13wk old	10.3	1.71	6	4.6	0.24	6	4.5%	4.31 [1.92, 6.70]
Li, Z., et al. (2020)-15wk old	10.5	1.3	15	5.9	1	9	5.1%	3.70 [2.29, 5.11]
Björnson Granqvist, A., et al. (2020)-20wk old	9.26	1.57	9	4.81	0.18	7	4.9%	3.53 [1.81, 5.25]
**Subtotal (95% CI)**			**100**			**98**	**43.0%**	**6.37 [4.65, 8.09]**
Heterogeneity: Tau² = 5.84; Chi² = 51.58, df = 10 (P < 0.00001); I² = 81%								
Test for overall effect: Z = 7.26 (P < 0.00001)								
**3.1.2 Genetic rat models**								
Habibi, J., et al. (2019)-26wk old	5	1.58	10	3.7	0.24	6	5.2%	0.96 [-0.12, 2.05]
**Subtotal (95% CI)**			**10**			**6**	**5.2%**	**0.96 [-0.12, 2.05]**
Heterogeneity: Not applicable								
Test for overall effect: Z = 1.75 (P = 0.08)								
**3.1.3 Chemical-induced mouse modelse**								
Glastras, S. J., et al. (2016)-lean-32wk old	5.85	0.24	9	4.51	0.06	9	4.2%	7.30 [4.45, 10.15]
Glastras, S. J., et al. (2016)-obese-32wk old	5.65	0.25	9	4.51	0.06	9	4.5%	5.97 [3.58, 8.37]
Tung, C. W., et al. (2019)-8wk DM	8.1	1.1	6	4.2	0.3	6	4.5%	4.47 [2.01, 6.92]
Glastras, S. J., et al. (2016)-32wk old	5.85	0.81	9	4.51	0.21	9	5.2%	2.16 [0.94, 3.38]
Glastras, S. J., et al. (2016)-HFD-32wk old	4.67	0.27	9	4.51	0.21	9	5.3%	0.63 [-0.32, 1.58]
**Subtotal (95% CI)**			**42**			**42**	**23.6%**	**3.85 [1.58, 6.12]**
Heterogeneity: Tau² = 5.62; Chi² = 35.51, df = 4 (P < 0.00001); I² = 89%								
Test for overall effect: Z = 3.32 (P = 0.0009)								
**3.1.4 Chemical-induced rat modelsrat**								
Xu, J., et al. (2014)-5wk -DM	8.7	0.8	8	3.6	0.2	5	3.7%	7.30 [3.76, 10.85]
Faisal Lutfi, M., et al. (2021)-8wk DM	9	0.7	6	4.5	0.5	6	3.7%	6.83 [3.31, 10.35]
Shiju, T. M., et al. (2013)-STZ 12wk	9.8	0.2	4	6	0.7	4	3.0%	6.42 [1.79, 11.05]
Gad, H. I. (2014)-2 mo DM	6.95	0.56	10	3.89	0.39	10	4.6%	6.07 [3.80, 8.35]
Wang, X., et al. (2019)-8wk DM	10.48	1.85	10	4.33	0.74	10	4.9%	4.18 [2.49, 5.87]
Nunes, S., et al. (2020)-23wk old	4	0.42	8	3.73	0.14	8	5.3%	0.82 [-0.22, 1.85]
**Subtotal (95% CI)**			**46**			**43**	**25.2%**	**4.98 [2.42, 7.55]**
Heterogeneity: Tau² = 8.14; Chi² = 38.26, df = 5 (P < 0.00001); I² = 87%								
Test for overall effect: Z = 3.81 (P = 0.0001)								
**3.1.5 Genetic mouse models with treatment**								
Zhang, H., et al. (2017)-14wk old	10.5	0.57	8	4.6	0.3	9	2.9%	12.53 [7.63, 17.43]
**Subtotal (95% CI)**			**8**			**9**	**2.9%**	**12.53 [7.63, 17.43]**
Heterogeneity: Not applicable								
Test for overall effect: Z = 5.01 (P < 0.00001)								
**3.1.6 Genetic rat models with treatment**								
**Subtotal (95% CI)**			**0**			**0**		**Not estimable**
Heterogeneity: Not applicable								
Test for overall effect: Not applicable								
**Total (95% CI)**			**206**			**198**	**100.0%**	**5.36 [4.16, 6.56]**
Heterogeneity: Tau² = 6.85; Chi² = 191.01, df = 23 (P < 0.00001); I² = 88%								
Test for overall effect: Z = 8.75 (P < 0.00001)								
Test for subgroup differences: Chi² = 45.52, df = 4 (P < 0.00001), I² = 91.2%								

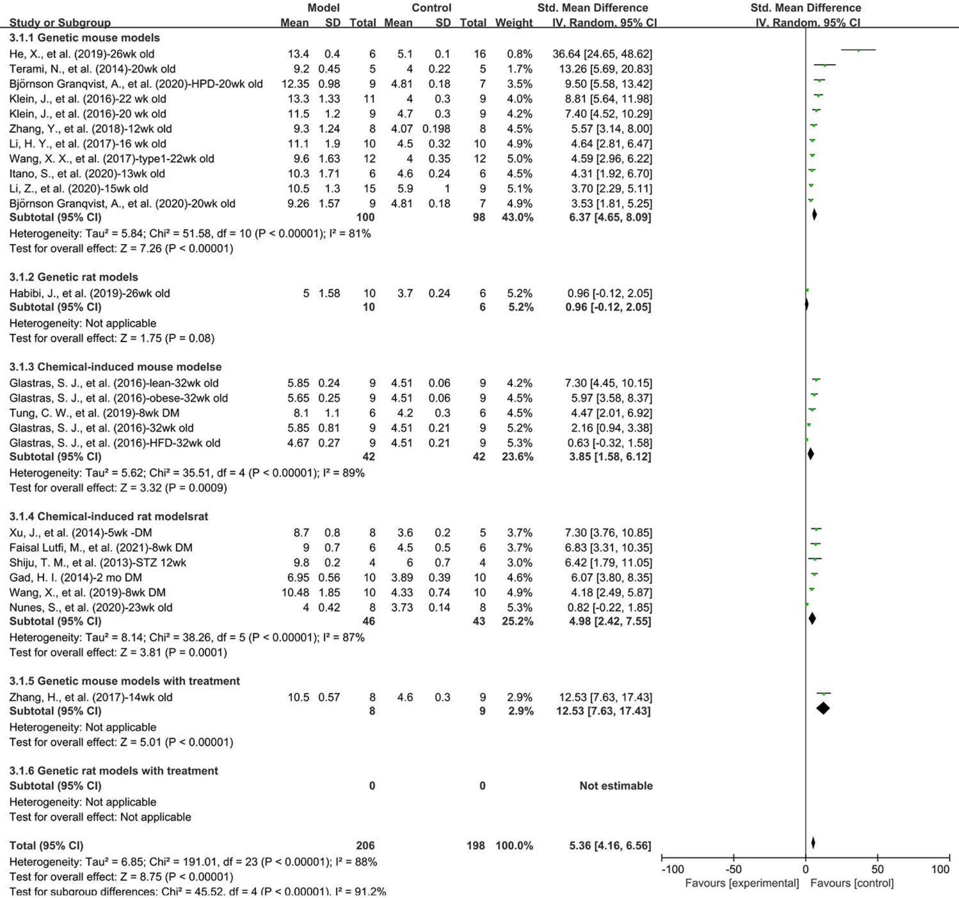

Supplementary Figure.3. Forest plot of HbA1c

	Model			Control				Std. Mean Difference
Study or Subgroup	Mean	SD	Total	Mean	SD	Total	Weight	IV, Random, 95% CI
**5.1.1 Genetic mouse models**								
Ma, K. L., et al. (2014)-16 wk old	129.93	6.57	10	94.74	4.64	10	2.6%	5.93 [3.70, 8.15]
Björnson Granqvist, A., et al. (2020)-HPD-20wk old	305.11	43.7	9	106.34	11.6	7	2.4%	5.54 [3.12, 7.97]
Björnson Granqvist, A., et al. (2020)-20wk old	243.62	32.48	9	106.34	11.6	7	2.6%	5.05 [2.81, 7.29]
Wang, X. X., et al. (2017)-type2-22wk old	128.8	49.54	12	53.4	10.74	12	3.6%	2.03 [1.01, 3.05]
Li, H. Y., et al. (2017)-16 wk old	118.19	16.16	10	93.18	8.6	10	3.6%	1.85 [0.77, 2.94]
Morigi, M., et al. (2020)-14wk old	130	11.31	8	117	6.71	5	3.4%	1.22 [-0.03, 2.47]
Cassis, P., et al. (2019)-19/20 wk old	217	111	9	119	15	9	3.6%	1.18 [0.16, 2.20]
Somineni, H. K., et al. (2014)-17wk old	116.01	18.94	6	116.01	18.94	6	3.5%	0.00 [-1.13, 1.13]
**Subtotal (95% CI)**			**73**			**66**	**25.3%**	**2.53 [1.34, 3.73]**
Heterogeneity: Tau² = 2.32; Chi² = 42.56, df = 7 (P < 0.00001); I² = 84%								
Test for overall effect: Z = 4.17 (P < 0.0001)								
**5.1.2 Genetic rat models**								
Habibi, J., et al. (2019)-26wk old	468	208.71	10	100	26.94	6	3.4%	2.07 [0.76, 3.38]
Toyoda, K., et al. (2018)-HFD-31wk old	97	9	3	74	10	3	2.4%	1.93 [-0.53, 4.39]
Toyoda, K., et al. (2018)-31wk old	99	21	3	74	10	3	2.8%	1.22 [-0.77, 3.20]
Lezcano, E. J., et al. (2014)-19 wk old	278.42	104.41	8	286.16	23.2	5	3.5%	-0.09 [-1.20, 1.03]
Domon, A., et al. (2019)-30wk old	81.3	16.4	4	108.8	5.6	4	2.8%	-1.95 [-3.88, -0.02]
**Subtotal (95% CI)**			**28**			**21**	**14.9%**	**0.61 [-0.80, 2.02]**
Heterogeneity: Tau² = 1.80; Chi² = 14.52, df = 4 (P = 0.006); I² = 72%								
Test for overall effect: Z = 0.85 (P = 0.40)								
**5.1.3 Chemical-induced mouse models**								
Glastras, S. J., et al. (2016)-HFD-32wk old	170.92	10.44	9	90.1	9.28	9	2.0%	7.79 [4.77, 10.82]
Ying, C., et al. (2021)-12 wk DM	358.86	82.37	8	159.71	42.15	8	3.2%	2.88 [1.37, 4.39]
Lopez-Parra, V., et al. (2012)-STZ-15wk	73	8	12	65	12	7	3.7%	0.80 [-0.18, 1.77]
Glastras, S. J., et al. (2016)-32wk old	92.81	22.04	9	90.1	9.28	9	3.7%	0.15 [-0.77, 1.08]
**Subtotal (95% CI)**			**38**			**33**	**12.5%**	**2.42 [0.36, 4.48]**
Heterogeneity: Tau² = 3.69; Chi² = 28.52, df = 3 (P < 0.00001); I² = 89%								
Test for overall effect: Z = 2.31 (P = 0.02)								
**5.1.4 Chemical-induced rat models**								
Shiju, T. M., et al. (2013)-STZ 12wk	201	5.9	4	112	5	4	0.3%	14.15 [4.32, 23.98]
Ibrahim, Z. S., et al. (2016)-8wk DM	95.3	0.47	8	72.3	2.59	8	1.1%	11.68 [6.92, 16.45]
Gargouri, M., et al. (2018)-3wk DM	240.4	20.09	6	122.3	7.84	6	1.6%	7.15 [3.48, 10.82]
Gad, H. I. (2014)-2 mo DM	68.665	2.914	10	50.884	2.231	10	2.4%	6.56 [4.13, 9.00]
Hu, S. J., et al. (2018)-8wk DM	935.04	239.96	10	148.76	11.51	12	3.0%	4.69 [2.95, 6.44]
Nasri, M., et al. (2020)-8wk DM	89.27	13.41	9	65.46	4.65	9	3.4%	2.26 [1.01, 3.50]
Chen, Y., et al. (2016)-8wk DM	292.73	123.24	12	110.21	34.18	10	3.6%	1.86 [0.83, 2.90]
Ali, M. A. M., et al. (2017)-STZ 60day	79	5.66	8	71	2.83	8	3.5%	1.69 [0.50, 2.88]
Ding, D., et al. (2016)-STZ 14wk	95.9	83.87	15	57.62	14.98	15	3.8%	0.62 [-0.12, 1.35]
Faisal Lutfi, M., et al. (2021)-8wk DM	117.5	13.3	6	112.2	3.2	6	3.5%	0.51 [-0.65, 1.66]
Huang, H., et al. (2017)-8wk DM	75.79	65.64	12	54.52	41.58	10	3.7%	0.36 [-0.48, 1.21]
Huang, H., et al. (2017)-4wk DM	86.23	77.69	12	63.81	20.79	10	3.7%	0.36 [-0.48, 1.21]
Dong, D., et al. (2019)-STZ 8wk	78.5	56.91	15	69.61	28.46	15	3.8%	0.19 [-0.53, 0.91]
Huang, H., et al. (2017)-12wk DM	59.55	52.24	12	54.52	23.23	10	3.7%	0.12 [-0.72, 0.96]
**Subtotal (95% CI)**			**139**			**133**	**41.3%**	**2.09 [1.19, 2.99]**
Heterogeneity: Tau² = 2.13; Chi² = 97.29, df = 13 (P < 0.00001); I² = 87%								
Test for overall effect: Z = 4.55 (P < 0.00001)								
**5.1.5 Genetic mouse models with treatment**								
Ma, K. L., et al. (2014)-inflame-16wk	128.38	5.03	10	94.74	4.64	10	2.4%	6.66 [4.19, 9.12]
Zhang, H., et al. (2017)-14wk old	198.6	72.69	8	108.8	5.1	9	3.5%	1.71 [0.55, 2.87]
**Subtotal (95% CI)**			**18**			**19**	**5.9%**	**4.06 [-0.78, 8.90]**
Heterogeneity: Tau² = 11.27; Chi² = 12.69, df = 1 (P = 0.0004); I² = 92%								
Test for overall effect: Z = 1.64 (P = 0.10)								
**5.1.6 Genetic rat models with treatment**								
**Subtotal (95% CI)**			**0**			**0**		**Not estimable**
Heterogeneity: Not applicable								
Test for overall effect: Not applicable								
**Total (95% CI)**			**296**			**272**	**100.0%**	**2.10 [1.51, 2.70]**
Heterogeneity: Tau² = 2.25; Chi² = 214.38, df = 32 (P < 0.00001); I² = 85%								
Test for overall effect: Z = 6.97 (P < 0.00001)								
Test for subgroup differences: Chi² = 5.37, df = 4 (P = 0.25), I² = 25.5%								

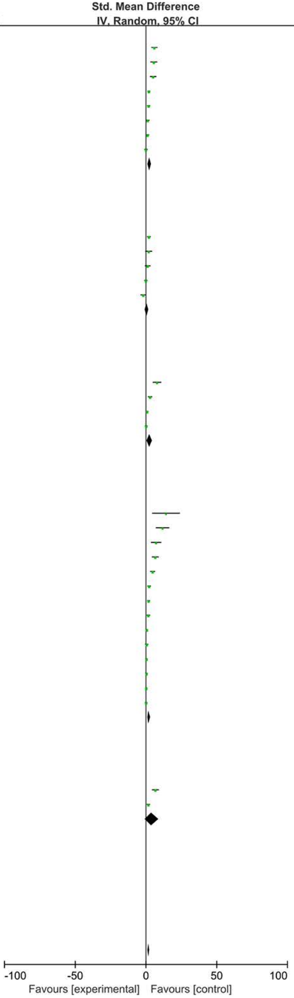

Supplementary Figure.4. Forest plot of TC

Study or Subgroup	Model Mean	Model SD	Model Total	Control Mean	Control SD	Control Total	Weight	Std. Mean Difference IV, Random, 95% CI
**4.1.1 Genetic mouse models**								
Chodavarapu, H., et al. (2013)-14wk old	274	60.5	6	94.5	12.49	6	2.5%	3.79 [1.62, 5.96]
Li, H. Y., et al. (2017)-16 wk old	138.66	23.15	10	75.03	20.9	10	3.5%	2.76 [1.47, 4.06]
Somineni, H. K., et al. (2014)-17wk old	274.49	86.76	6	115.11	21.69	6	3.2%	2.33 [0.72, 3.93]
Cassis, P., et al. (2019)-19/20 wk old	179	24	9	118	27	9	3.6%	2.27 [1.03, 3.52]
Wang, X. X., et al. (2017)-type2-22wk old	215.3	99.42	12	64.3	10.74	12	3.9%	2.06 [1.04, 3.09]
Ma, K. L., et al. (2014)-16 wk old	108.02	23.91	10	77.03	4.43	10	3.8%	1.73 [0.67, 2.79]
Morigi, M., et al. (2020)-14wk old	238	73.54	8	135	6.71	5	3.5%	1.63 [0.28, 2.98]
**Subtotal (95% CI)**			**61**			**58**	**24.0%**	**2.18 [1.69, 2.66]**
Heterogeneity: Tau² = 0.00; Chi² = 4.35, df = 6 (P = 0.63); I² = 0%								
Test for overall effect: Z = 8.78 (P < 0.00001)								
**4.1.2 Genetic rat models**								
Toyoda, K., et al. (2018)-HFD-31wk old	452	66	3	134	69	3	1.2%	3.77 [-0.21, 7.74]
Toyoda, K., et al. (2018)-31wk old	773	263	3	134	69	3	1.8%	2.66 [-0.37, 5.68]
Habibi, J., et al. (2019)-26wk old	2,000	1,245.94	10	84	29.39	6	3.6%	1.81 [0.57, 3.06]
Lezcano, E. J., et al. (2014)-19 wk old	1,016	654	8	365	202	5	3.6%	1.13 [-0.10, 2.36]
**Subtotal (95% CI)**			**24**			**17**	**10.2%**	**1.66 [0.83, 2.48]**
Heterogeneity: Tau² = 0.00; Chi² = 2.27, df = 3 (P = 0.52); I² = 0%								
Test for overall effect: Z = 3.94 (P < 0.0001)								
**4.1.3 Chemical-induced mouse models**								
Ying, C., et al. (2021)-12 wk DM	224.02	21.25	8	71.72	15.94	8	1.6%	7.67 [4.45, 10.88]
Glastras, S. J., et al. (2016)-HFD-32wk old	74.38	26.56	9	61.98	31.88	9	4.0%	0.40 [-0.53, 1.34]
Glastras, S. J., et al. (2016)-lean-32wk old	59.33	177.98	9	22.14	490.54	9	4.0%	0.10 [-0.83, 1.02]
Glastras, S. J., et al. (2016)-obese-32wk old	38.07	63.75	9	22.14	490.54	9	4.0%	0.04 [-0.88, 0.97]
Glastras, S. J., et al. (2016)-32wk old	44.27	39.85	9	61.98	31.88	9	4.0%	-0.47 [-1.41, 0.47]
**Subtotal (95% CI)**			**44**			**44**	**17.6%**	**0.71 [-0.48, 1.89]**
Heterogeneity: Tau² = 1.38; Chi² = 23.02, df = 4 (P = 0.0001); I² = 83%								
Test for overall effect: Z = 1.17 (P = 0.24)								
**4.1.4 Chemical-induced rat models**								
Gad, H. I. (2014)-2 mo DM	169.47	6.344	10	75.445	2.967	10	0.6%	18.18 [11.83, 24.53]
Shiju, T. M., et al. (2013)-STZ 12wk	112	5	4	50	5	4	0.4%	10.78 [3.24, 18.33]
Gargouri, M., et al. (2018)-3wk DM	256.7	24.25	6	97.8	12.98	6	1.3%	7.54 [3.69, 11.39]
Hu, S. J., et al. (2018)-8wk DM	145.21	41.62	10	48.7	14.17	12	3.5%	3.11 [1.80, 4.43]
Nasri, M., et al. (2020)-8wk DM	102	16.53	9	60	9.78	9	3.4%	2.95 [1.52, 4.37]
Ali, M. A. M., et al. (2017)-STZ 60day	46	2.83	8	38	2.83	8	3.4%	2.67 [1.22, 4.12]
Chen, Y., et al. (2016)-8wk DM	541.01	122.7	12	265.635	151.2	10	3.9%	1.94 [0.89, 3.00]
Ding, D., et al. (2016)-STZ 14wk	224.02	96.02	15	75.26	65.16	15	4.1%	1.76 [0.90, 2.63]
Ibrahim, Z. S., et al. (2016)-8wk DM	63.3	12.5	8	52.3	8	8	3.8%	0.99 [-0.07, 2.05]
Huang, H., et al. (2017)-12wk DM	155.84	150.3	12	54.01	30.8	10	4.0%	0.86 [-0.02, 1.75]
Huang, H., et al. (2017)-4wk DM	268.29	361.94	12	49.56	44.8	10	4.1%	0.78 [-0.10, 1.66]
Huang, H., et al. (2017)-8wk DM	226.68	319	12	84.12	89.6	10	4.1%	0.56 [-0.30, 1.42]
Dong, D., et al. (2019)-STZ 8wk	236.43	456.1	15	87.66	68.59	15	4.2%	0.44 [-0.28, 1.17]
Faisal Lutfi, M., et al. (2021)-8wk DM	108.8	17.7	6	124.6	2.1	6	3.6%	-1.16 [-2.42, 0.11]
**Subtotal (95% CI)**			**139**			**133**	**44.2%**	**1.89 [1.03, 2.74]**
Heterogeneity: Tau² = 1.91; Chi² = 86.69, df = 13 (P < 0.00001); I² = 85%								
Test for overall effect: Z = 4.34 (P < 0.0001)								
**4.1.5 Genetic mouse models with treatment**								
Ma, K. L., et al. (2014)-inflame-16wk	103.6	25.68	10	77.03	4.43	10	3.9%	1.38 [0.38, 2.38]
**Subtotal (95% CI)**			**10**			**10**	**3.9%**	**1.38 [0.38, 2.38]**
Heterogeneity: Not applicable								
Test for overall effect: Z = 2.71 (P = 0.007)								
**4.1.6 Genetic rat models with treatment**								
**Subtotal (95% CI)**			**0**			**0**		**Not estimable**
Heterogeneity: Not applicable								
Test for overall effect: Not applicable								
**Total (95% CI)**			**278**			**262**	**100.0%**	**1.73 [1.22, 2.24]**
Heterogeneity: Tau² = 1.47; Chi² = 152.05, df = 30 (P < 0.00001); I² = 80%								
Test for overall effect: Z = 6.64 (P < 0.00001)								
Test for subgroup differences: Chi² = 6.37, df = 4 (P = 0.17), I² = 37.2%								

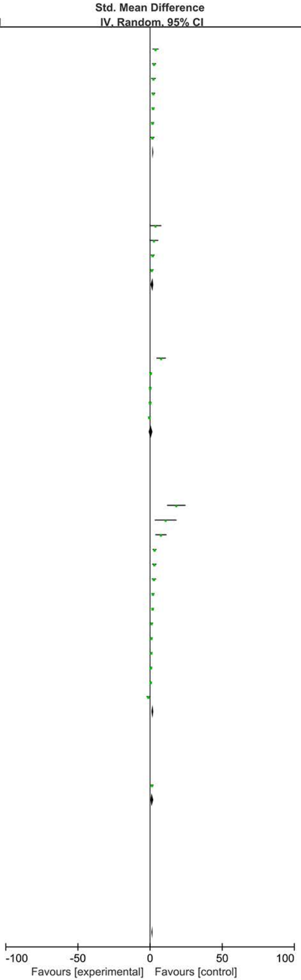

Supplementary Figure.5. Forest plot of TG

Study or Subgroup	Model Mean	Model SD	Model Total	Control Mean	Control SD	Control Total	Weight	Std. Mean Difference IV, Random, 95% CI
**6.1.1 Genetic mouse models**								
He, X., et al. (2019)-26wk old	0.77	0.06	6	0.43	0.03	16	1.4%	8.24 [5.39, 11.09]
Zhang, X., et al. (2020)-ZnT8-KOdb/db-24wk old	1.21	0.33	6	0.168	0.064	6	1.6%	4.05 [1.77, 6.32]
Li, H. Y., et al. (2017)-16 wk old	0.4	0.01	10	0.32	0.03	10	1.9%	3.43 [1.95, 4.90]
Zhang, X., et al. (2020)-type1-24wk old	0.896	0.29	6	0.168	0.064	6	1.7%	3.20 [1.27, 5.13]
Pichaiwong, W., et al. (2013)-24wk old	0.29	0.2	6	0.17	0.04	5	2.0%	0.72 [-0.52, 1.97]
Kim, J. E., et al. (2013)-4wk calestrol	0.59	0.22	8	0.46	0.15	8	2.0%	0.65 [-0.36, 1.67]
Terami, N., et al. (2014)-20wk old	0.12	0.04	5	0.1	0.02	5	2.0%	0.57 [-0.71, 1.85]
Itano, S., et al. (2020)-13wk old	0.11	0.01	6	0.13	0.01	6	1.9%	-1.85 [-3.29, -0.40]
Björnson Granqvist, A., et al. (2020)-HPD-20wk old	0.045	0.011	9	0.08	0.011	7	1.9%	-3.01 [-4.56, -1.45]
Björnson Granqvist, A., et al. (2020)-20wk old	0.043	0.007	9	0.08	0.011	7	1.8%	-3.91 [-5.76, -2.07]
**Subtotal (95% CI)**			**71**			**76**	**18.2%**	**1.07 [-0.65, 2.79]**
Heterogeneity: Tau² = 6.92; Chi² = 112.18, df = 9 (P < 0.00001); I² = 92%								
Test for overall effect: Z = 1.22 (P = 0.22)								
**6.1.2 Genetic rat models**								
Habibi, J., et al. (2019)-26wk old	0.45	0.28	10	0.32	0.05	6	2.0%	0.54 [-0.49, 1.58]
Domon, A., et al. (2019)-30wk old	0.43	0.05	4	0.43	0.05	4	1.9%	0.00 [-1.39, 1.39]
Lezcano, E. J., et al. (2014)-19 wk old	0.32	0.1	8	0.34	0.11	5	2.0%	-0.18 [-1.30, 0.94]
**Subtotal (95% CI)**			**22**			**15**	**6.0%**	**0.16 [-0.50, 0.83]**
Heterogeneity: Tau² = 0.00; Chi² = 0.93, df = 2 (P = 0.63); I² = 0%								
Test for overall effect: Z = 0.48 (P = 0.63)								
**6.1.3 Chemical-induced mouse models**								
Lopez-Parra, V., et al. (2012)-STZ-15wk	0.38	0.03	12	0.22	0.02	7	1.6%	5.68 [3.45, 7.91]
Ying, C., et al. (2021)-12 wk DM	0.3	0.055	8	0.11	0.017	8	1.7%	4.41 [2.40, 6.43]
Dusabimana, T., et al. (2020)-6wk-STZ	4.116	1.12	10	0.95	0.15	8	1.9%	3.56 [1.95, 5.18]
Zhang, X., et al. (2020)-STZ-24wk old	1.04	0.296	8	0.36	0.141	8	1.9%	2.77 [1.29, 4.25]
Glastras, S. J., et al. (2016)-lean-32wk old	0.38	0.05	9	0.31	0.02	9	2.0%	1.75 [0.62, 2.88]
Yang, S., et al. (2016)-2wk DM	1.43	0.63	6	0.75	0.46	6	2.0%	1.14 [-0.12, 2.40]
Glastras, S. J., et al. (2016)-obese-32wk old	0.37	0.07	9	0.31	0.02	9	2.0%	1.11 [0.10, 2.12]
Lian, H., et al. (2019)-4wk DM	0.89	0.11	6	0.82	0.09	6	2.0%	0.64 [-0.53, 1.82]
Mohamed, R., et al. (2013)-12wk DM	0.14	0.08	8	0.1	0.11	8	2.0%	0.39 [-0.60, 1.39]
Uil, M., et al. (2018)-STZ 12wk	0.144	0.046	7	0.135	0.045	10	2.0%	0.19 [-0.78, 1.16]
Uil, M., et al. (2018)-STZ 12wk-western diet	0.139	0.025	8	0.135	0.045	10	2.1%	0.10 [-0.83, 1.03]
**Subtotal (95% CI)**			**91**			**89**	**21.2%**	**1.74 [0.89, 2.59]**
Heterogeneity: Tau² = 1.60; Chi² = 51.76, df = 10 (P < 0.00001); I² = 81%								
Test for overall effect: Z = 4.01 (P < 0.0001)								
**6.1.4 Chemical-induced rat models**								
Shiju, T. M., et al. (2013)-STZ 12wk	1.74	0.03	4	0.9	0.05	4	0.2%	17.72 [5.45, 29.98]
Ibrahim, Z. S., et al. (2016)-8wk DM	2.3	0.061	8	1.09	0.07	8	0.5%	17.42 [10.40, 24.45]
Maheshwari, R. A., et al. (2014)-STZ 7wk	2.1	0.03	6	0.63	0.12	6	0.4%	15.51 [7.86, 23.17]
Sadar, S., et al. (2016)-STZ 4wk	0.75	0.04	5	0.2	0.04	5	0.5%	12.42 [5.32, 19.52]
Elseweidy, M. M., et al. (2013)-8wk DM	2.5	0.3	8	0.23	0.04	8	1.0%	10.03 [5.91, 14.15]
Couto, S. M. F., et al. (2020)-4wk DM	0.92	0.06	7	0.3	0.06	7	1.0%	9.67 [5.32, 14.03]
Alzahrani, S., et al. (2020)-8wk DM	4.065	0.48	6	0.572	0.1	5	0.9%	8.78 [4.05, 13.50]
Ali, M. A. M., et al. (2017)-STZ 60day	3.93	0.4	8	0.95	0.23	8	1.2%	8.64 [5.05, 12.22]
Fernandes, S. M., et al. (2016)-UNx-85 days DM	0.64	0.05	6	0.22	0.04	6	1.0%	8.56 [4.23, 12.89]
Chen, H. W., et al. (2019)-6wk DM	1.4	0.09	8	0.77	0.05	8	1.2%	8.18 [4.77, 11.59]
Ibrahim, D. S. (2015)-30Day DM	0.53	0.06	6	0.19	0.01	6	1.1%	7.30 [3.56, 11.03]
Chen, Y., et al. (2016)-8wk DM	1.4	0.09	12	0.77	0.08	10	1.6%	7.08 [4.62, 9.53]
Hu, S. J., et al. (2018)-STZ 1wk	1.44	0.28	10	0.1	0.05	12	1.6%	6.73 [4.38, 9.08]
Hu, S. J., et al. (2018)-8wk DM	1.58	0.33	10	0.56	0.16	12	1.9%	3.91 [2.38, 5.43]
Garud, M. S. and Y. A. Kulkarni (2018)-STZ 8wk	110.65	13.35	8	70.93	6.3	8	1.8%	3.60 [1.86, 5.34]
Amin, S. N., et al. (2020)-6wk DM	1.468	0.502	6	0.163	0.047	6	1.7%	3.38 [1.38, 5.38]
Gargouri, M., et al. (2018)-3wk DM	1.1	0.13	6	0.7	0.12	6	1.8%	2.95 [1.12, 4.78]
Sathibabu Uddandrao, V. V., et al. (2019) 45daysDN	2.89	0.92	6	0.69	0.4	6	1.8%	2.86 [1.06, 4.66]
Souza, C. S., et al. (2019)-9wk DM	1.87	0.54	6	0.71	0.06	6	1.8%	2.79 [1.02, 4.56]
Goru, S. K., et al. (2017)-STZ 8wk	2.81	0.44	8	1.73	0.28	8	1.9%	2.77 [1.29, 4.25]
Wang, X., et al. (2019)-8wk DM	0.38	0.04	10	0.29	0.03	10	2.0%	2.44 [1.22, 3.65]
Fernandes, S. M., et al. (2016)-85 days DM	0.55	0.18	6	0.22	0.04	6	1.9%	2.34 [0.73, 3.94]
Faisal Lutfi, M., et al. (2021)-8wk DM	1.2	0.2	6	0.9	0.1	6	1.9%	1.75 [0.33, 3.17]
Xu, J., et al. (2014)-5wk -DM	0.71	0.22	8	0.39	0.04	5	1.9%	1.68 [0.32, 3.04]
Huang, H., et al. (2017)-4wk DM	0.41	0.1	12	0.3	0.07	10	2.1%	1.21 [0.28, 2.13]
Huang, H., et al. (2017)-8wk DM	0.49	0.15	12	0.38	0.1	10	2.1%	0.81 [-0.07, 1.69]
Huang, H., et al. (2017)-12wk DM	0.54	0.34	12	0.38	0.1	10	2.1%	0.59 [-0.27, 1.45]
Nasri, M., et al. (2020)-8wk DM	0.75	0.36	9	0.6	0.18	9	2.1%	0.50 [-0.44, 1.44]
Nunes, S., et al. (2020)-23wk old	0.38	0.06	8	0.36	0.03	8	2.0%	0.40 [-0.59, 1.39]
Thisted, L., et al. (2020)-px unx 8wk	0.28	0.03	11	0.31	0.027	12	2.1%	-1.02 [-1.90, -0.14]
Zakaria, E. M., et al. (2017)-10wk DM	71.2	13.1	8	89.8	14.7	8	2.0%	-1.26 [-2.37, -0.16]
**Subtotal (95% CI)**			**246**			**239**	**46.9%**	**3.81 [2.88, 4.74]**
Heterogeneity: Tau² = 5.16; Chi² = 278.73, df = 30 (P < 0.00001); I² = 89%								
Test for overall effect: Z = 8.00 (P < 0.00001)								
**6.1.5 Genetic mouse models with treatment**								
Zhang, X., et al. (2020)-ZnT8-KO-24wk old	1.05	0.314	8	0.36	0.141	8	1.9%	2.68 [1.23, 4.13]
Zhang, H., et al. (2017)-14wk old	0.123	0.013	7	0.09	0.016	5	1.9%	2.13 [0.58, 3.68]
Wang, L., et al. (2014)-20wk old	0.32	0.118	5	0.2	0.12	9	2.0%	0.94 [-0.23, 2.11]
**Subtotal (95% CI)**			**20**			**22**	**5.8%**	**1.84 [0.76, 2.91]**
Heterogeneity: Tau² = 0.41; Chi² = 3.66, df = 2 (P = 0.16); I² = 45%								
Test for overall effect: Z = 3.34 (P = 0.0008)								
**6.1.6 Genetic rat models with treatment**								
te Riet, L., et al. (2014)-5wk DM	0.31	0.03	8	0.43	0.06	8	1.9%	-2.39 [-3.76, -1.02]
**Subtotal (95% CI)**			**8**			**8**	**1.9%**	**-2.39 [-3.76, -1.02]**
Heterogeneity: Not applicable								
Test for overall effect: Z = 3.43 (P = 0.0006)								
**Total (95% CI)**			**458**			**449**	**100.0%**	**2.35 [1.77, 2.92]**
Heterogeneity: Tau² = 3.94; Chi² = 499.26, df = 58 (P < 0.00001); I² = 88%								
Test for overall effect: Z = 8.02 (P < 0.00001)								
Test for subgroup differences: Chi² = 68.75, df = 5 (P < 0.00001), I² = 92.7%								

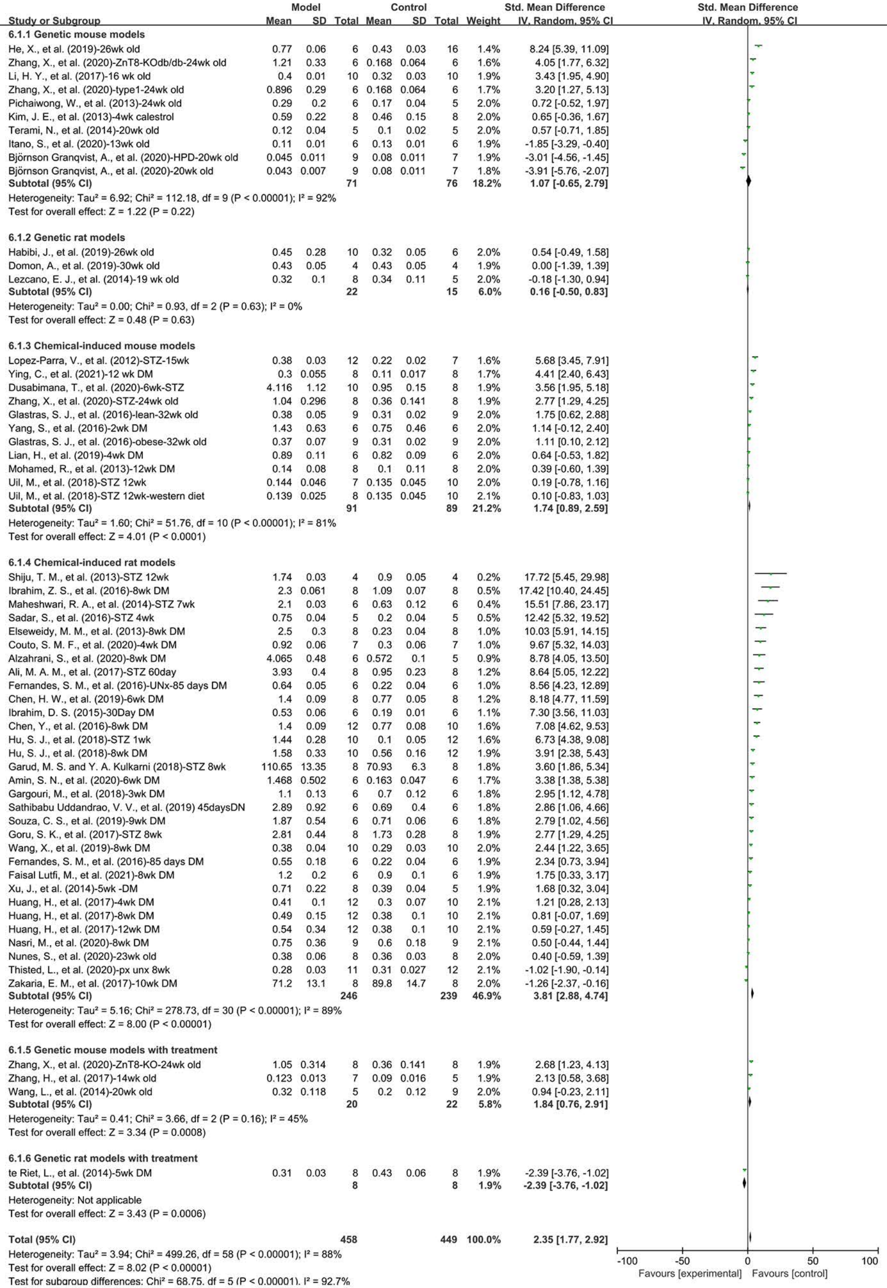

Supplementary Figure.6. Forest plot of SCR

Study or Subgroup	Model Mean	Model SD	Model Total	Control Mean	Control SD	Control Total	Weight	Std. Mean Difference IV, Random, 95% CI
**7.1.1 Genetic mouse models**								
Zhang, X., et al. (2020)-type1-24wk old	30.23	1.3	6	14.88	1.3	6	0.7%	10.90 [5.46, 16.34]
Pichaiwong, W., et al. (2013)-24wk old	64.07	27.36	6	13.83	2.75	5	2.7%	2.24 [0.58, 3.91]
Terami, N., et al. (2014)-20wk old	29.1	6.26	5	20.3	1.57	5	2.7%	1.74 [0.16, 3.32]
Cassis, P., et al. (2019)-19/20 wk old	37.3	19.5	9	24.6	4.5	9	3.4%	0.85 [-0.12, 1.83]
Zhang, X., et al. (2020)-ZnT8-KOdb/db-24wk old	36.45	57.1	6	14.88	1.3	6	3.2%	0.49 [-0.66, 1.65]
**Subtotal (95% CI)**			**32**			**31**	**12.6%**	**1.80 [0.40, 3.21]**
Heterogeneity: Tau² = 1.71; Chi² = 16.08, df = 4 (P = 0.003); I² = 75%								
Test for overall effect: Z = 2.51 (P = 0.01)								
**7.1.2 Genetic rat models**								
Domon, A., et al. (2019)-30wk old	18.1	6.7	4	15	2.7	4	2.9%	0.53 [-0.90, 1.96]
**Subtotal (95% CI)**			**4**			**4**	**2.9%**	**0.53 [-0.90, 1.96]**
Heterogeneity: Not applicable								
Test for overall effect: Z = 0.72 (P = 0.47)								
**7.1.3 Chemical-induced mouse models**								
Lopez-Parra, V., et al. (2012)-STZ-15wk	37.3	2.7	12	27.1	1.1	7	2.5%	4.30 [2.50, 6.09]
Ying, C., et al. (2021)-12 wk DM	46.79	9.61	8	21.3	5.56	8	2.8%	3.07 [1.50, 4.64]
Hong, G. L., et al. (2021)-STZ 12wk	41.5	4.47	5	29.8	5.14	5	2.6%	2.19 [0.44, 3.94]
Zhang, X., et al. (2020)-STZ-24wk old	65.48	39.94	8	17.46	5.14	8	3.2%	1.59 [0.43, 2.76]
Yang, S., et al. (2016)-2wk DM	27.91	9.28	6	15.14	6.98	6	3.0%	1.44 [0.10, 2.77]
**Subtotal (95% CI)**			**39**			**34**	**14.0%**	**2.40 [1.41, 3.38]**
Heterogeneity: Tau² = 0.67; Chi² = 8.69, df = 4 (P = 0.07); I² = 54%								
Test for overall effect: Z = 4.77 (P < 0.00001)								
**7.1.4 Chemical-induced rat models**								
Ibrahim, D. S. (2015)-30Day DM	49.03	0.85	6	28.72	1.44	6	0.3%	15.86 [8.03, 23.68]
Shiju, T. M., et al. (2013)-STZ 12wk	59	3.5	4	15	0.5	4	0.2%	15.30 [4.69, 25.92]
Sathibabu Uddandrao, V. V., et al. (2019) 45daysDN	43.1	1.8	6	18.2	1.15	6	0.4%	15.22 [7.70, 22.73]
Sadar, S., et al. (2016)-STZ 4wk	63.02	4.45	5	10.37	3.73	5	0.5%	11.58 [4.94, 18.22]
Liu, W., et al. (2013)-16wk DM	59.07	6.89	6	18.89	3.5	6	1.3%	6.79 [3.29, 10.29]
Garud, M. S. and Y. A. Kulkarni (2018)-STZ 8wk	41.81	4.99	8	18.44	2.14	8	1.9%	5.76 [3.26, 8.25]
Goru, S. K., et al. (2017)-STZ 8wk	45.81	5.55	8	21.58	7.13	8	2.6%	3.59 [1.85, 5.32]
Amin, S. N., et al. (2020)-6wk DM	92.8	18.783	6	39.017	11.133	6	2.4%	3.22 [1.28, 5.15]
Hu, S. J., et al. (2018)-STZ 1wk	45.2	12.89	10	16.17	4.62	12	3.0%	3.00 [1.71, 4.29]
Hu, S. J., et al. (2018)-8wk DM	42.76	12.38	10	15.83	5.18	12	3.1%	2.83 [1.58, 4.08]
Wang, X., et al. (2019)-8wk DM	11.52	1.98	10	6.82	1.45	10	3.1%	2.59 [1.34, 3.85]
Chen, H. W., et al. (2019)-6wk DM	22.83	5.99	8	11.42	1.24	8	2.9%	2.49 [1.10, 3.89]
Chen, Y., et al. (2016)-8wk DM	8.38	4.66	12	2.3	2.22	10	3.4%	1.55 [0.57, 2.53]
Huang, H., et al. (2017)-4wk DM	36.76	20.67	12	14.01	4.43	10	3.4%	1.40 [0.45, 2.36]
Huang, H., et al. (2017)-8wk DM	39.68	29.9	12	16.84	2.66	10	3.4%	0.99 [0.09, 1.89]
Huang, H., et al. (2017)-12wk DM	44.33	46.4	12	15.8	2.84	10	3.4%	0.80 [-0.08, 1.67]
Dong, D., et al. (2019)-STZ 8wk	36.2	30.49	15	22.98	3.47	15	3.6%	0.59 [-0.14, 1.33]
Nunes, S., et al. (2020)-23wk old	8.57	1.7	8	14.19	1.64	8	2.7%	-3.18 [-4.78, -1.58]
**Subtotal (95% CI)**			**158**			**154**	**41.6%**	**2.71 [1.73, 3.69]**
Heterogeneity: Tau² = 3.10; Chi² = 126.07, df = 17 (P < 0.00001); I² = 87%								
Test for overall effect: Z = 5.42 (P < 0.00001)								
**7.1.5 Genetic mouse models with treatment**								
Han, X., et al. (2015)-TauT+/- 5mo DM	32.8	10.75	10	12.8	6.64	10	3.2%	2.14 [1.00, 3.29]
Hong, G. L., et al. (2021)-Nqo1 KO-STZ 12wk	51.9	13.64	5	29.8	5.14	5	2.7%	1.94 [0.29, 3.59]
Maekawa, M., et al. (2022)-40wk old	46.6	19.8	5	20.4	1.7	5	2.8%	1.68 [0.12, 3.24]
Han, X., et al. (2015) TauT-/- 5mo DM	31.7	9.17	10	17.2	8.85	10	3.3%	1.54 [0.52, 2.57]
Zhang, X., et al. (2020)-ZnT8-KO-24wk old	70.3	57.3	8	17.46	4.91	8	3.2%	1.23 [0.13, 2.32]
Han, X., et al. (2015)-5mo DM	20.3	8.85	10	12.2	5.69	10	3.4%	1.04 [0.09, 1.99]
Han, X., et al. (2015)-TauT+/- 3mo DM	21.6	6.32	10	12.9	9.49	10	3.4%	1.03 [0.09, 1.98]
Han, X., et al. (2015)-TauT-/- 3mo DM	22.8	6.64	10	15.2	8.85	10	3.4%	0.93 [-0.00, 1.86]
Han, X., et al. (2015)-3mo DM	15.6	6.64	10	12.9	51.4	10	3.4%	0.07 [-0.81, 0.95]
**Subtotal (95% CI)**			**78**			**78**	**28.8%**	**1.16 [0.74, 1.58]**
Heterogeneity: Tau² = 0.11; Chi² = 10.90, df = 8 (P = 0.21); I² = 27%								
Test for overall effect: Z = 5.44 (P < 0.00001)								
**7.1.6 Genetic rat models with treatment**								
**Subtotal (95% CI)**			**0**			**0**		**Not estimable**
Heterogeneity: Not applicable								
Test for overall effect: Not applicable								
**Total (95% CI)**			**311**			**301**	**100.0%**	**1.94 [1.45, 2.42]**
Heterogeneity: Tau² = 1.57; Chi² = 173.76, df = 37 (P < 0.00001); I² = 79%								
Test for overall effect: Z = 7.84 (P < 0.00001)								
Test for subgroup differences: Chi² = 13.47, df = 4 (P = 0.009), I² = 70.3%								

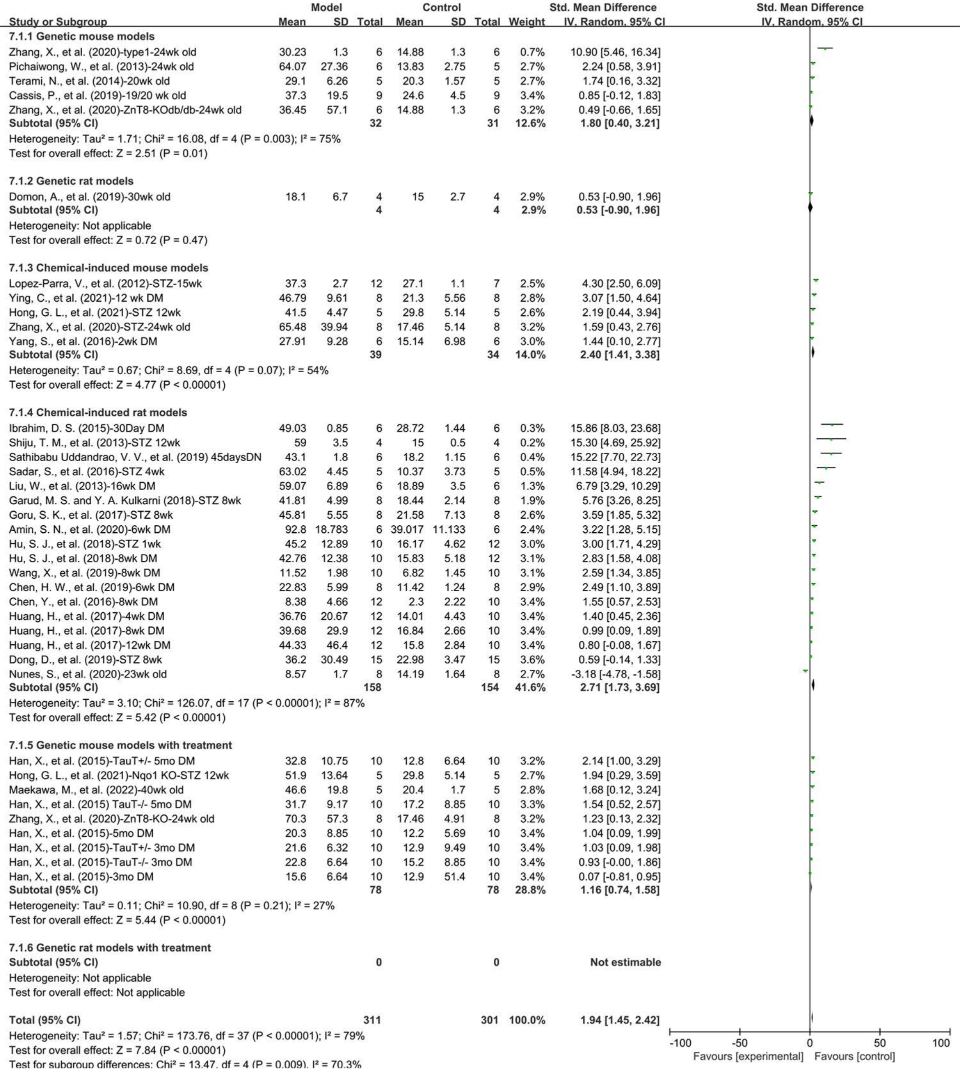

Supplementary Figure.7. Forest plot of BUN

Study or Subgroup	Experimental Mean	Experimental SD	Experimental Total	Control Mean	Control SD	Control Total	Weight	Std. Mean Difference IV, Random, 95% CI
**14.1.1 Genetic mouse models**								
Zhang, Y., et al. (2018)-12wk old	0.3938	0.146	8	0.0416	0.015	8	4.0%	3.21 [1.60, 4.82]
Itano, S., et al. (2020)-13wk old	0.217	0.071	6	0.02	0.011	4	3.3%	3.15 [0.97, 5.32]
Barati, M. T., et al. (2016)-6mo old	54.38	35.81	8	0.099	0.02	8	4.5%	2.03 [0.76, 3.30]
Pichaiwong, W., et al. (2013)-24wk old	0.741	0.41	6	0.085	0.036	5	4.1%	1.96 [0.39, 3.52]
Barati, M. T., et al. (2016)-2mo old	0.255	0.1	12	0.103	0.03	7	4.7%	1.76 [0.64, 2.89]
**Subtotal (95% CI)**			**40**			**32**	**20.4%**	**2.21 [1.57, 2.86]**
Heterogeneity: Tau² = 0.00; Chi² = 2.97, df = 4 (P = 0.56); I² = 0%								
Test for overall effect: Z = 6.74 (P < 0.00001)								
**14.1.2 Genetic rat models**								
Lezcano, E. J., et al. (2014)-19 wk old	323.63	134.3	8	37.2	17.2	5	4.0%	2.48 [0.88, 4.07]
Toyoda, K., et al. (2018)-HFD-31wk old	6.1	1.3	3	4.6	4.6	3	4.0%	0.36 [-1.28, 1.99]
Toyoda, K., et al. (2018)-31wk old	3.7	0.6	3	4.6	4.6	3	4.0%	-0.22 [-1.83, 1.39]
**Subtotal (95% CI)**			**14**			**11**	**12.0%**	**0.88 [-0.74, 2.49]**
Heterogeneity: Tau² = 1.36; Chi² = 6.02, df = 2 (P = 0.05); I² = 67%								
Test for overall effect: Z = 1.06 (P = 0.29)								
**14.1.3 Chemical-induced mouse models**								
Lian, H., et al. (2019)-4wk DM	0.175	0.01	6	0.035	0.003	6	0.5%	17.51 [8.89, 26.13]
Mohamed, R., et al. (2013)-12wk DM	0.171	0.045	8	0.032	0.006	8	3.6%	4.09 [2.19, 6.00]
Glastras, S. J., et al. (2016)-obese-32wk old	42.66	22.65	9	7.71	5.61	3	4.1%	1.58 [0.06, 3.10]
Glastras, S. J., et al. (2016)-lean-32wk old	31.01	21	9	7.71	5.61	3	4.3%	1.13 [-0.28, 2.55]
Zhao, J., et al. (2019)-8wk DN	0.02409	0.042	7	0.00073	0.0011	7	4.7%	0.74 [-0.36, 1.83]
Zhao, J., et al. (2019)-4wk DN	0.00283	0.0058	7	0.00022	0.0004	7	4.7%	0.59 [-0.48, 1.67]
**Subtotal (95% CI)**			**46**			**34**	**21.9%**	**1.94 [0.49, 3.38]**
Heterogeneity: Tau² = 2.28; Chi² = 24.68, df = 5 (P = 0.0002); I² = 80%								
Test for overall effect: Z = 2.62 (P = 0.009)								
**14.1.4 Chemical-induced rat models**								
Ndisang, J. F. and A. Jadhav (2014)-8wk DM	52.7	6.12	6	2.1	0.73	6	1.1%	10.72 [5.36, 16.07]
Shiju, T. M., et al. (2013)-STZ 12wk	41.7	5.2	4	1.9	0.1	4	0.7%	9.41 [2.79, 16.03]
Li, X., et al. (2017)-STZ 25wk	24.96	2.56	8	10.22	1.67	10	2.7%	6.67 [4.03, 9.30]
Liu, W., et al. (2013)-16wk DM	9.69	1.77	6	1.28	0.14	6	2.2%	6.18 [2.96, 9.41]
Couto, S. M. F., et al. (2020)-4wk DM	39.42	8.89	7	3.76	0.91	7	2.8%	5.28 [2.75, 7.82]
Huang, H., et al. (2017)-12wk DM	0.93	0.17	12	0.18	0.13	10	3.8%	4.71 [2.96, 6.46]
Alomari, G., et al. (2020)-drug 7wk	0.68	0.16	7	0.14	0.11	7	3.6%	3.68 [1.76, 5.60]
Huang, H., et al. (2017)-4wk DM	0.87	0.59	12	0.18	0.16	10	4.9%	1.47 [0.51, 2.44]
Xu, J., et al. (2014)-5wk -DM	6.19	4.78	8	1.31	0.42	5	4.5%	1.19 [-0.06, 2.43]
Xin, R., et al. (2018)-4wk DM	30.8	18.6	9	15.8	7.2	9	4.8%	1.01 [0.02, 2.01]
Dong, D., et al. (2019)-STZ 8wk	4.73	7.82	15	0.41	0.54	15	5.1%	0.76 [0.01, 1.50]
Huang, H., et al. (2017)-8wk DM	0.88	1.63	12	0.18	0.16	10	5.0%	0.55 [-0.30, 1.41]
**Subtotal (95% CI)**			**106**			**99**	**41.2%**	**3.17 [2.02, 4.32]**
Heterogeneity: Tau² = 2.90; Chi² = 75.63, df = 11 (P < 0.00001); I² = 85%								
Test for overall effect: Z = 5.39 (P < 0.00001)								
**14.1.5 Genetic mouse models with treatment**								
Huang, C., et al. (2018)-38 week old	49.19	29.13	7	15.68	9.16	7	4.5%	1.45 [0.23, 2.68]
**Subtotal (95% CI)**			**7**			**7**	**4.5%**	**1.45 [0.23, 2.68]**
Heterogeneity: Not applicable								
Test for overall effect: Z = 2.32 (P = 0.02)								
**14.1.6 Genetic rat models with treatment**								
**Subtotal (95% CI)**			**0**			**0**		**Not estimable**
Heterogeneity: Not applicable								
Test for overall effect: Not applicable								
**Total (95% CI)**			**213**			**183**	**100.0%**	**2.26 [1.65, 2.87]**
Heterogeneity: Tau² = 1.78; Chi² = 116.16, df = 26 (P < 0.00001); I² = 78%								
Test for overall effect: Z = 7.22 (P < 0.00001)								
Test for subgroup differences: Chi² = 6.74, df = 4 (P = 0.15), I² = 40.6%								

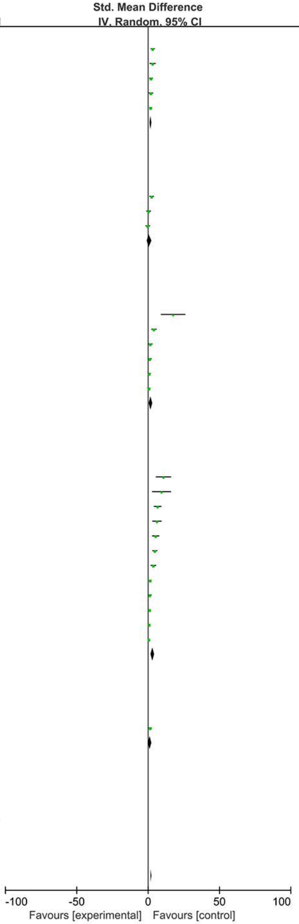

Supplementary Figure.8. Forest plot of UAE

Supplementary Table 1 Pubmed search strategy

#1. (("diabetic nephropathies"[MeSH Terms] OR ("diabetic"[All Fields] AND "nephropathies"[All Fields]) OR "diabetic nephropathies"[All Fields] OR ("diabetic nephropathies"[MeSH Terms] OR ("diabetic"[All Fields] AND "nephropathies"[All Fields]) OR "diabetic nephropathies"[All Fields] OR ("nephropathies"[All Fields] AND "diabetic"[All Fields]) OR "nephropathies diabetic"[All Fields]) OR ("diabetic nephropathies"[MeSH Terms] OR ("diabetic"[All Fields] AND "nephropathies"[All Fields]) OR "diabetic nephropathies"[All Fields] OR ("nephropathy"[All Fields] AND "diabetic"[All Fields]) OR "nephropathy diabetic"[All Fields]) OR ("diabetic nephropathies"[MeSH Terms] OR ("diabetic"[All Fields] AND "nephropathies"[All Fields]) OR "diabetic nephropathies"[All Fields] OR ("diabetic"[All Fields] AND "nephropathy"[All Fields]) OR "diabetic kidney disease"[All Fields]) OR ("diabetic nephropathies"[MeSH Terms] OR ("diabetic"[All Fields] AND "nephropathies"[All Fields]) OR "diabetic nephropathies"[All Fields] OR ("diabetic"[All Fields] AND "kidney"[All Fields] AND "disease"[All Fields]) OR "diabetic kidney disease"[All Fields]) OR ("diabetic nephropathies"[MeSH Terms] OR ("diabetic"[All Fields] AND "nephropathies"[All Fields]) OR "diabetic nephropathies"[All Fields] OR ("diabetic"[All Fields] AND "kidney"[All Fields] AND "diseases"[All Fields]) OR "diabetic kidney diseases"[All Fields]) OR ("diabetic nephropathies"[MeSH Terms] OR ("diabetic"[All Fields] AND "nephropathies"[All Fields]) OR "diabetic nephropathies"[All Fields] OR ("kidney"[All Fields] AND "disease"[All Fields] AND "diabetic"[All Fields]) OR "kidney disease diabetic"[All Fields]) OR ("diabetic nephropathies"[MeSH Terms] OR ("diabetic"[All Fields] AND "nephropathies"[All Fields]) OR "diabetic nephropathies"[All Fields] OR ("kidney"[All Fields] AND "diseases"[All Fields] AND "diabetic"[All Fields]) OR "kidney diseases diabetic"[All Fields]) OR ("diabetic nephropathies"[MeSH Terms] OR ("diabetic"[All Fields] AND "nephropathies"[All Fields]) OR "diabetic nephropathies"[All Fields] OR ("diabetic"[All Fields] AND "glomerulosclerosis"[All Fields]) OR "diabetic glomerulosclerosis"[All Fields]) OR ("diabetic nephropathies"[MeSH Terms] OR ("diabetic"[All Fields] AND "nephropathies"[All Fields]) OR "diabetic nephropathies"[All Fields] OR ("glomerulosclerosis"[All Fields] AND "diabetic"[All Fields]) OR "glomerulosclerosis diabetic"[All Fields]) OR ("diabetic nephropathies"[MeSH Terms] OR ("diabetic"[All Fields] AND "nephropathies"[All Fields]) OR "diabetic nephropathies"[All Fields] OR ("intracapillary"[All Fields] AND "glomerulosclerosis"[All Fields]) OR "intracapillary glomerulosclerosis"[All Fields]) OR ("diabetic nephropathies"[MeSH Terms] OR ("diabetic"[All Fields] AND "nephropathies"[All Fields]) OR "diabetic nephropathies"[All Fields] OR ("nodular"[All Fields] AND "glomerulosclerosis"[All Fields]) OR "nodular glomerulosclerosis"[All Fields]) OR ("diabetic nephropathies"[MeSH Terms] OR ("diabetic"[All Fields] AND "nephropathies"[All Fields]) OR "diabetic nephropathies"[All Fields] OR ("glomerulosclerosis"[All Fields] AND "nodular"[All Fields]) OR "glomerulosclerosis nodular"[All Fields]) OR ("diabetic nephropathies"[MeSH Terms] OR ("diabetic"[All Fields] AND "nephropathies"[All Fields]) OR "diabetic nephropathies"[All Fields] OR ("kimmelstiel"[All Fields] AND "wilson"[All Fields] AND "syndrome"[All Fields]) OR "kimmelstiel wilson syndrome"[All Fields]) OR ("diabetic nephropathies"[MeSH Terms] OR ("diabetic"[All Fields] AND "nephropathies"[All Fields]) OR "diabetic nephropathies"[All Fields] OR ("kimmelstiel"[All Fields] AND "wilson"[All Fields] AND "syndrome"[All Fields]) OR "kimmelstiel wilson syndrome"[All Fields]) OR ("diabetic nephropathies"[MeSH Terms] OR ("diabetic"[All Fields] AND "nephropathies"[All Fields]) OR "diabetic nephropathies"[All Fields] OR ("kimmelstiel"[All Fields] AND "wilson"[All Fields] AND "disease"[All Fields]) OR "kimmelstiel wilson disease"[All Fields]) OR ("diabetic nephropathies"[MeSH Terms] OR ("diabetic"[All Fields] AND "nephropathies"[All Fields]) OR "diabetic nephropathies"[All Fields] OR ("kimmelstiel"[All Fields] AND "wilson"[All Fields] AND

"disease"[All Fields]) OR "kimmelstiel wilson disease"[All Fields]))

#2. ((("animals"[MeSH Terms:noexp] OR "animals"[All Fields]) NOT ("human s"[All Fields] OR "humans"[MeSH Terms] OR "humans"[All Fields] OR "human"[All Fields])) OR ("mice"[MeSH Terms] OR "mice"[All Fields] OR "mouse"[All Fields] OR "mouse s"[All Fields] OR "mouses"[All Fields]) OR ("mice"[MeSH Terms] OR "mice"[All Fields]) OR ("rats"[MeSH Terms] OR "rats"[All Fields]) OR ("rats"[MeSH Terms] OR "rats"[All Fields] OR "rat"[All Fields]))

#3. ("animal experimentation"[MeSH Terms] OR ("animal"[All Fields] AND "experimentation"[All Fields]) OR "animal experimentation"[All Fields] OR ("animal experimentation"[MeSH Terms] OR ("animal"[All Fields] AND "experimentation"[All Fields]) OR "animal experimentation"[All Fields] OR ("experimentation"[All Fields] AND "animal"[All Fields]) OR "experimentation animal"[All Fields]) OR ("animal experimentation"[MeSH Terms] OR ("animal"[All Fields] AND "experimentation"[All Fields]) OR "animal experimentation"[All Fields] OR ("animal"[All Fields] AND "research"[All Fields]) OR "animal research"[All Fields]) OR ("animal experimentation"[MeSH Terms] OR ("animal"[All Fields] AND "experimentation"[All Fields]) OR "animal experimentation"[All Fields] OR ("research"[All Fields] AND "animal"[All Fields]) OR "research animal"[All Fields]) OR ("animal experimentation"[MeSH Terms] OR ("animal"[All Fields] AND "experimentation"[All Fields]) OR "animal experimentation"[All Fields] OR ("animal"[All Fields] AND "experimental"[All Fields]) OR "animal experimental use"[All Fields]) OR ("animal experimentation"[MeSH Terms] OR ("animal"[All Fields] AND "experimentation"[All Fields]) OR "animal experimentation"[All Fields] OR ("animal"[All Fields] AND "experimental"[All Fields] AND "uses"[All Fields])) OR ("animal experimentation"[MeSH Terms] OR ("animal"[All Fields] AND "experimentation"[All Fields]) OR "animal experimentation"[All Fields] OR ("experimental"[All Fields] AND "animal"[All Fields])) OR ("animal experimentation"[MeSH Terms] OR ("animal"[All Fields] AND "experimentation"[All Fields]) OR "animal experimentation"[All Fields] OR ("experimental"[All Fields] AND "uses"[All Fields] AND "animal"[All Fields])) OR ("animal experimentation"[MeSH Terms] OR ("animal"[All Fields] AND "experimentation"[All Fields]) OR "animal experimentation"[All Fields] OR ("animal"[All Fields] AND "experiments"[All Fields]) OR "animal experiments"[All Fields]) OR ("animal experimentation"[MeSH Terms] OR ("animal"[All Fields] AND "experimentation"[All Fields]) OR "animal experimentation"[All Fields] OR ("animal"[All Fields] AND "experiment"[All Fields]) OR "animal experiment"[All Fields]) OR ("animal experimentation"[MeSH Terms] OR ("animal"[All Fields] AND "experimentation"[All Fields]) OR "animal experimentation"[All Fields] OR ("experiment"[All Fields] AND "animal"[All Fields]) OR "experiment animal"[All Fields]) OR ("animal experimentation"[MeSH Terms] OR ("animal"[All Fields] AND "experimentation"[All Fields]) OR "animal experimentation"[All Fields] OR ("experiments"[All Fields] AND "animal"[All Fields]) OR "experiments animal"[All Fields])))

#4. (y_10[Filter])

#5. #1 And #2 And #3 And #4

Supplementary file1 84-included studies

Ali, M. A. M., et al. (2017). "Modulation of heme oxygenase-1 expression and activity affects streptozotocin-induced diabetic nephropathy in rats." Fundam Clin Pharmacol 31(5): 546-557.
Control: N = 8 Model: N = 8

Heme oxygenase (HO)-1 has exhibited nephro-protective actions in different animal models; however, its full mechanistic potential in diabetic nephropathy (DN) has not yet been elucidated. Hence, the present study has been undertaken by inducing DN in rats using streptozotocin (50 mg/kg i.p.), with or without either HO-1 inducer; hemin (HM; 40 μmol/kg, s.c.), or HO-1 blocker; zinc protoporphyrin-IX (ZnPP; 50 μmol/kg, i.p.), for one month. Compared to control, rats with DN suffered from hyperglycemia and hyperlipidemia, with signs of renal damage, as assessed by distortion in renal histopathologic architecture and kidney function. Renal oxidative/nitrosative stress was evident by increased malondialdehyde, nitric oxide, myeloperoxidase, with decreased reduced glutathione, superoxide dismutase, and catalase. DN group also exhibited high renal expression of the pro-inflammatory cytokine; tumor necrosis factor (TNF)-α, and the apoptotic marker; caspase 3, assessed by Western blot. Renal HO-1 protein expression and activity were increased in DN rats compared to control. Administration of HM, but not ZnPP, to DN rats improved kidney function, histopathologic features, lipid profile, TNF-α, and caspase 3 expressions, with no effect on blood glucose level. HM increased, while ZnPP decreased renal HO-1 activity in DN rats. It is noteworthy that neither intervention affected HO-1 activity or renal oxidative capacity in non-diabetic rats. Interestingly, the expression of HO-1 was upregulated by both HM and ZnPP in DN rats. In conclusion, activation of HO-1 via HM ameliorated renal damage in STZ-induced DN in rats, probably through antioxidant, anti-nitrosative, anti-inflammatory, and anti-apoptotic mechanisms.

Alomari, G., et al. (2020). "Gold nanoparticles attenuate albuminuria by inhibiting podocyte injury in a rat model of diabetic nephropathy." Drug Deliv Transl Res 10(1): 216-226.
Control: N = 7 Model: N = 7

Several recent studies have reported that gold nanoparticles (AuNPs) attenuate hyperglycemia in diabetic animal models without any observed side effects. The present study was intended to provide insight into the effects of 50-nm AuNPs on diabetic kidney disease. Adult male rats were divided into three groups (n = 7/group): control (non-diabetic, ND), diabetic (D), and diabetic treated intraperitoneally with 50-nm AuNPs (AuNPs + D; 2.5 mg/kg/day) for 7 weeks. Diabetes was induced by a single-dose injection of 55 mg/kg streptozotocin. The result showed that AuNP treatment prevented diabetes-associated increases in the blood glucose level. Reduction in 24-h urinary albumin excretion rate, glomerular basement membrane thickness, foot process width, and renal oxidative stress markers was also demonstrated in the AuNP-treated group. In addition, the results showed downregulation effect of AuNPs in renal mRNA or protein expression of transforming growth factor β1 (TGF-β(1)), fibronectin, collagen IV, tumor necrosis factor-α (TNF-α), and vascular endothelial growth factor-A (VEGF-A). Moreover, the protein expression of nephrin and podocin, podocyte markers, in glomeruli was increased in the AuNPs + D group compared with the D group. These results provide evidence that 50-nm AuNPs can ameliorate renal damage in experimental models of diabetic nephropathy through improving the renal function and downregulating extracellular matrix protein accumulation, along with inhibiting renal oxidative stress and amelioration of podocyte injury.

Alzahrani, S., et al. (2020). "Protective effect of isoliquiritigenin on experimental diabetic nephropathy in rats: Impact on Sirt-1/NFκB balance and NLRP3 expression." Int Immunopharmacol 87: 106813.
Control: N = 6 Model: N = 6

The prevalence of diabetes mellitus (DM) drastically increases worldwide. Persistent hyperglycemia affects body microvasculature causing injuries to kidney producing diabetic nephropathy (DNE). Manifestation of these microvascular complications is associated with disturbed redox homeostasis. The current study evaluated the effect of isoliquiritigenin (ISLQ), a bioactive chalcone found in licorice which is known for its antioxidant effect, on diabetes-induced renal injury. DM was prompted in male rats by streptozotocin (STZ, 50 mg/kg, intraperitoneally). ISLQ was administrated by oral gavage for 8 weeks at a dose (20 mg/kg/day). Features of renal injury were observed in kidneys of diabetic rats including, albuminuria and deteriorated renal function. Renal dysfunction was associated with reduced sirtuin-1 (Sirt-1) expression, increased renal oxidative stress, nucleotide-binding domain and leucine-rich repeat containing protein-3 (NLRP3), nuclear factor-κB (NFκB) and inflammatory cytokines interleukin-1β (IL-1β) and tumor necrosis factor-α (TNF-α). Moreover, there was significant downregulation of anti-inflammatory cytokine interleukin-10 (IL-10), glomerular and tubular injury and collagen accumulation. ISLQ administration preserved renal function and architecture, restored Sirt1 and renal oxidant-antioxidant balance, dampened inflammation and attenuated collagen accumulation. It can be inferred that ISLQ possess a protective effect and could have a potential as a food supplement to halt development and

progression of DNE.

Amin, S. N., et al. (2020). "Inhibition of notch signalling and mesangial expansion by combined glucagon like peptide-1 agonist and crocin therapy in animal model of diabetic nephropathy." Arch Physiol Biochem: 1-11.
Control: N = 6 Model: N = 6

Diabetic nephropathy (DN) is one of the devastating complications in diabetes mellitus (DM). Glucagon-like peptide-1 (GLP-1) is one of the incretins secreted from L cells in the intestine. Crocin (a carotenoid component of saffron) has antioxidants properties. We investigated the renal effects of Exendin-4 as a GLP-1 agonist and Crocin in DN.Thirty male rats were divided into five groups: control, type II DM, type II DM + Exendin-4, type II DM + Crocin and type II DM + Exendine-4 + Crocin. At the end of the experimental period, systolic and diastolic blood pressures were measured, and GFR was calculated. Blood and urine samples were collected for biochemical analysis. Tissue samples were collected from the kidney for histological examination and biochemical measurements of protein expression.Treatment with GLP-1 agonist or Crocin caused a significant improvement in renal function. Better results were achieved with simultaneous administration of both drugs with inhibition of notch signalling pathway and the related proteins.

Arimura, E., et al. (2018). "Effects of Diets with Different Proportions of Protein/Carbohydrate on Retinal Manifestations in db Mice." In Vivo 32(2): 265-272.
Control: N = 6 Model: N = 6

BACKGROUND/AIM: Diabetic nephropathy is aggravated by a higher intake of total protein. The effects of diets with different proportions of protein and carbohydrate on diabetic retinopathy in db mice, a type-2 diabetes animal model, were examined, as well as diabetic nephropathy. MATERIALS AND METHODS: Control and db mice at 5 weeks of age were fed the diets (% energy of protein/carbohydrate/fat; L-diet: 12/71/17; H-diet: 24/59/17) under ad libitum conditions and pair-feeding conditions for 6 weeks, respectively. RESULTS: Mice fed the H-diet showed significantly greater retinal thickness by optical coherence tomography, and lower mRNA levels of angiotensinogen. Comparing combinations of diets and genotypes, db-H mice showed significantly higher mRNA levels of angiotensin-converting enzyme, advanced glycosylation end product-specific receptor, and cluster of differentiation molecule 11b (a microglial marker) than db-L mice. CONCLUSION: Dietary protein and carbohydrate proportions influenced retinal manifestations, including retinal thickness and gene expression in control and diabetic mice.

Barati, M. T., et al. (2016). "Differential expression of endoplasmic reticulum stress-response proteins in different renal tubule subtypes of OVE26 diabetic mice." Cell Stress Chaperones 21(1): 155-166.
Control: N = 8 Model: N = 8

Regulation of the endoplasmic reticulum (ER) stress-response pathway during the course of diabetes specifically in renal tubules is unclear. Since tubule cell dysfunction is critical to progression of diabetic nephropathy, this study analyzed markers of ER stress response and ER chaperones at different stages of diabetes and in different renal tubule subtypes of OVE26 type-1 diabetic mice. ER stress-responseinduced chaperones GRP78, GRP94, and protein disulfide isomerase (PDI) were increased in isolated cortical tubules of older diabetic mice, while PDI was decreased in tubules of young diabetic mice. Immunofluorescence staining of kidneys from older mice showed GRP78 and PDI upregulation in all cortical tubule segments, with substantial induction of PDI in distal tubules. Protein kinase RNA-like endoplasmic reticulum kinase (PERK) phosphorylation was increased in cortical tubules of young diabetic mice, with no differences between older diabetic and control mice. Expression of ER stress-induced PERK inhibitor p58IPK was decreased and then increased in all tubule subtypes of young and older mice, respectively. Knockdown of PERK by small interfering RNA (siRNA) increased fibronectin secretion in cultured proximal tubule cells. Tubules of older diabetic mice had significantly more apoptotic cells, and ER stress-induced proapoptotic transcription factor C/EBP homologous protein (CHOP) was increased in proximal and distal tubules of diabetic mice and diabetic humans. CHOP induction in OVE26 mice was not altered by severity of proteinuria. Overexpression of CHOP in cultured proximal tubule cells increased expression of fibronectin. These findings demonstrate differential ER stress-response signaling in tubule subtypes of diabetic mice and implicate a role for PERK and CHOP in tubule cell matrix protein production.

Björnson Granqvist, A., et al. (2020). "High-protein diet accelerates diabetes and kidney disease in the BTBRob/ob mouse." Am J Physiol Renal Physiol 318(3): F763-f771.
Control: N = 7 Model: N = 9

There is a need for improved animal models that better translate to human kidney disease to

predict outcome of pharmacological effects in the patient. The diabetic BTBRob/ob mouse model mimics key features of early diabetic nephropathy in humans, but with chronic injury limited to glomeruli. To explore if we could induce an accelerated and more advanced disease phenotype that closer translates to human disease, we challenged BTBRob/ob mice with a high-protein diet (HPD; 30%) and followed the progression of metabolic and renal changes up to 20 wk of age. Animals on the HPD showed enhanced metabolic derangements, evidenced by further increased levels of glucose, HbA(1C), cholesterol, and alanine aminotransferase. The urinary albumin-to-creatinine ratio was markedly increased with a 53-fold change compared with lean controls, whereas BTBRob/ob mice on the standard diet only presented an 8-fold change. HPD resulted in more advanced mesangial expansion already at 14 wk of age compared with BTBRob/ob mice on the standard diet and also aggravated glomerular pathology as well as interstitial fibrosis. Gene expression analysis revealed that HPD triggered expression of markers of fibrosis and inflammation in the kidney and increased oxidative stress markers in urine. This study showed that HPD significantly aggravated renal injury in BTBRob/ob mice by further advancing albuminuria, glomerular, and tubulointerstitial pathology by 20 wk of age. This mouse model offers closer translation to humans and enables exploration of new end points for pharmacological efficacy studies that also holds promise to shorten study length.

Cassis, P., et al. (2019). "Addition of cyclic angiotensin-(1-7) to angiotensin-converting enzyme inhibitor therapy has a positive add-on effect in experimental diabetic nephropathy." Kidney Int 96(4): 906-917.
Control: N = 9 Model: N = 9

The Renin-Angiotensin System (RAS) possesses a counter-regulatory axis composed of angiotensin converting enzyme (ACE)2, angiotensin-(1-7) [Ang-(1-7)] and the Mas receptor, which opposes many AT1-receptor-mediated effects of ligand angiotensin II. Ang-(1-7), as a ligand of the Mas receptor, has inhibitory effects on renal inflammation and fibrosis in experimental diabetes. However, Ang-(1-7) has a short half-life in plasma, which may render it unsuitable for use in clinics. Here, we investigated the effects of the lanthionine-stabilized Ang-(1-7), cyclic (c)Ang-(1-7), a lanthipeptide that is more peptidase-resistant than the linear peptide, in BTBR ob/ob mice with type 2 diabetic nephropathy. BTBR ob/ob mice received vehicle, cAng-(1-7), or the ACE inhibitor lisinopril. The treatment started at ten weeks of age, when the animals had already developed albuminuria, and ended at 19-20 weeks of age. cAng-(1-7) limited albuminuria progression, and limited podocyte dysfunction similarly to lisinopril. cAng-(1-7), unlike lisinopril, reduced glomerular fibrosis and inflammation, and counteracted glomerular capillary rarefaction. Furthermore, when cAng-(1-7) was combined with lisinopril, a superior antiproteinuric effect than with lisinopril alone was found, in association with better preservation of podocyte proteins and amelioration of capillary density. Thus, adding cAng-(1-7) to ACE-inhibitor therapy could benefit those diabetic patients who do not respond completely to ACE-inhibitor therapy.

Chen, H. W., et al. (2019). "Nelumbo nucifera leaves extract attenuate the pathological progression of diabetic nephropathy in high-fat diet-fed and streptozotocin-induced diabetic rats." J Food Drug Anal 27(3): 736-748.
Control: N = 8 Model: N = 8

Diabetic nephropathy is not only a common and severe microvascular complication of diabetes mellitus but also the leading cause of renal failure. Lotus (Nelumbo nucifera) possesses antioxidative and anticancer properties. The present study aimed to investigate the antidiabetic and renoprotective effects of N. nucifera leaf extract (NLE) in a rat model of type 2 diabetic mellitus. Male Sprague-Dawley rats with type 2 diabetes induced by a high-fat diet (HFD)/streptozotocin (STZ) were treated with NLE at dosages of 0.5% and 1% (w/w) daily for 6 weeks. At the end of the experimental period, body weight, serum glucose levels, insulin levels, and kidney function were assessed. Furthermore, antioxidant enzyme and lipid peroxide levels were determined in the kidney, and histopathological examination was performed using hematoxylin and eosin staining, periodic acid Schiff staining, and Masson trichrome staining. To shed light on the molecular mechanism underlying the functioning of NLE, mouse glomerular mesangial cells (MES-13) treated with high glucose (HG, 25 mM glucose) were chosen as a model for an examination of the signal transduction pathway of NLE. The results revealed that NLE improved diabetic kidney injury by reducing blood glucose, serum creatinine, and blood urea nitrogen levels and enhanced antioxidant enzyme activities in kidney tissue. Treatment with NLE significantly reduced the malondialdehyde and 8-hydroxy-2-deoxyguanosine levels and increased serum insulin levels; expression of renal superoxide dismutase, catalase, and glutathione peroxidase activities; and glutathione content. Histological studies have also demonstrated that NLE treatment inhibited the dilation of Bowman's capsule, which confirmed its renoprotective action in diabetes. In addition, treatment with NLE and its major component quercetin 3-glucuronide attenuated 25 mM HG-induced suppressed nuclear factor erythroid 2-related factor 2 and antioxidant enzyme expression in MES-13 cells. Collectively, these

findings indicate that NLE may have antidiabetic and renoprotective effects against HFD/STZ-induced diabetes, at least in part, through antioxidative pathways.

Chen, Y., et al. (2016). "Evaluating Pharmacological Effects of Two Major Components of Shuangdan Oral Liquid: Role of Danshensu and Paeonol in Diabetic Nephropathy Rat." Biomol Ther (Seoul) 24(5): 536-542.
Control: N = 10 Model: N = 12

Shuangdan oral liquid (SDO) containing radix Salviae miltiorrhizae (Chinese name Danshen) and cortex moutan (Chinese name Mudanpi) is a traditional Chinese medicine using for treating vascular diseases. Danshensu (DSS) is a main effective monomer composition derived from radix Salviae miltiorrhizae and paeonol (Pae) from cortex moutan. Although the two herbs are widely used in traditional Chinese medicine, the pharmacological functions of their active compositions were not reported. Therefore, the research of DSS and Pae in mechanisms and pharmacodynamics interaction can provide scientific evidence to support clinical application. The diabetic nephropathy (DN) rats which were induced by streptozotocin (STZ) were treated with SDO, DSS, Pae, and DSS+Pae for eight weeks. The positive effects on DN animal models were investigated by detection of physiological and biochemical indexes and oxidative stress markers, within five treatments: SDO, DSS, Pae, DSS+Pae and insulin group. Compared with the model group, the DSS+Pae group improved the renal function, blood lipid metabolism and blood viscosity, increased the vitality of T-SOD or T-AOC and decreased the level of MDA or NO after the treatment. The study was successfully showed that the DSS+Pae group could delay the process of DN, especially in the renal injury part of histopathology changes. Our results suggest that the co-administration of DSS and Pae significantly may play a protective role in DN rats through decreasing the oxidative stress and improving the blood lipid metabolism mechanisms.

Chodavarapu, H., et al. (2013). "Rosiglitazone treatment of type 2 diabetic db/db mice attenuates urinary albumin and angiotensin converting enzyme 2 excretion." PLoS One 8(4): e62833.
Control: N = 6 Model: N = 6

Alterations within the renal renin angiotensin system play a pivotal role in the development and progression of cardiovascular and renal disease. Angiotensin converting enzyme 2 (ACE2) is highly expressed in renal tubules and has been shown to be renoprotective in diabetes. The protease, a disintegrin and metalloprotease (ADAM) 17, is involved in the ectodomain shedding of several transmembrane proteins including ACE2. Renal ACE2 and ADAM17 were significantly increased in db/db mice compared to controls. We investigated the effect of the insulin sensitizer, rosiglitazone, on albuminuria, renal ADAM17 protein expression and ACE2 shedding in db/db diabetic mice. Rosiglitazone treatment of db/db mice normalized hyperglycemia, attenuated renal injury and decreased urinary ACE2 and renal ADAM17 protein expression. Urinary excreted ACE2 is enzymatically active. Western blot analysis of urinary ACE2 demonstrated two prominent immunoreactive bands at approximately 70 & 90 kDa. The predominant immunoreactive band is approximately 20 kDa shorter than the one demonstrated for kidney lysate, indicating possible ectodomain shedding of active renal ACE2 in the urine. Therefore, it is tempting to speculate that renoprotection of rosiglitazone could be partially mediated via downregulation of renal ADAM17 and ACE2 shedding. In addition, there was a positive correlation between blood glucose, urinary albumin, plasma glucagon, and triglyceride levels with urinary ACE2 excretion. In conclusion, urinary ACE2 could be used as a sensitive biomarker of diabetic nephropathy and for monitoring the effectiveness of renoprotective medication.

Christensen, M., et al. (2019). "Metformin attenuates renal medullary hypoxia in diabetic nephropathy through inhibition uncoupling protein-2." Diabetes Metab Res Rev 35(2): e3091.
Control: N = 6 Model: N = 6

BACKGROUND: The purpose of the study is to examine the effect of metformin on oxygen metabolism and mitochondrial function in the kidney of an animal model of insulinopenic diabetes in order to isolate any renoprotective effect from any concomitant effect on blood glucose homeostasis. METHODS: Sprague-Dawley rats were injected with streptozotocin (STZ) (50 mg kg(-1)) and when stable started on metformin treatment (250 mg kg(-1)) in the drinking water. Rats were prepared for in vivo measurements 25 to 30 days after STZ injection, where renal function, including glomerular filtration rate and sodium transport, was estimated in anesthetized rats. Intrarenal oxygen tension was measured using oxygen sensors. Furthermore, mitochondrial function was assessed in mitochondria isolated from kidney cortex and medulla analysed by high-resolution respirometry, and superoxide production was evaluated using electron paramagnetic resonance. RESULTS: Insulinopenic rats chronically treated with metformin for 4 weeks displayed improved medullary tissue oxygen tension despite of no effect of metformin on blood glucose homeostasis. Metformin reduced UCP2-dependent LEAK and differentially affected medullary mitochondrial superoxide radical production in control and diabetic rats.

CONCLUSIONS: Metformin attenuates diabetes-induced renal medullary tissue hypoxia in an animal model of insulinopenic type 1 diabetes. The results suggest that the mechanistic pathway to attenuate the diabetes-induced medullary hypoxia is independent of blood glucose homeostasis and includes reduced UCP2-mediated mitochondrial proton LEAK.

Couto, S. M. F., et al. (2020). "Physical Training Is a Potential Modifier of Risk for Contrast-Induced Acute Kidney Injury in Diabetes Mellitus." Biomed Res Int 2020: 1830934.
Control: N = 7 Model: N = 7

BACKGROUND: Iodinated contrast (IC) is a leading cause of hospital-based acute kidney injury (AKI). Contrast-induced acute kidney injury (CI-AKI) is a decline in renal function due to iodinated contrast administration and occurs more frequently in individuals with increasingly common risk factors, such as diabetes mellitus (DM). Physical training (PT) can have renoprotective effects on CI-AKI in diabetic nephropathy. The aim of this study was to evaluate the injury in kidneys of diabetic rats submitted to treatment with IC, evaluating the impact of PT on hemodynamics and renal function in addition to oxidative profile in diabetic rats submitted to IC-AKI. MATERIALS AND METHODS: Adult male Wistar rats are randomized into four groups: citrate (n = 7): control group, citrate buffer (streptozotocin-STZ vehicle), intravenous tail (iv), single dose; DM (n = 7): STZ, 60 mg/kg, iv, single dose; DM+IC (n = 7): DM rats treated with IC (sodium meglumine ioxithalamate, 6 mL/kg, intraperitoneal (ip), single dose); DM+IC+PT (n = 7): DM rats treated with IC as mentioned and submitted to physical training. Renal function parameters (inulin clearance, neutrophil gelatinase-associated lipocalin (NGAL), serum creatinine, and urinary albumin), hemodynamics (renal blood flow and renal vascular resistance), and oxidative profile (urinary peroxides, urinary TBARS, urinary nitric oxide, and renal tissue thiols) were evaluated. RESULTS: It was possible to observe a decrease in inulin clearance, renal blood flow, and thiols in renal tissue accompanied by an increase in urinary flow, serum creatinine, urinary albumin, renal vascular resistance, urinary peroxides, urinary nitrate, and TBARS in the DM group compared to the citrate group. The DM+IC group showed a reduction in inulin clearance, and the renal dysfunction was also seen by the increased NGAL. Renal hemodynamics and oxidative profile compared were also worsened in the DM group. PT improved renal function by increasing renal blood flow and thiol levels in renal tissue and reduced renal vascular resistance, metabolites of reactive oxygen, nitrogen species, and lipid peroxidation in the DM+IC+PT group compared to DM+IC. CONCLUSIONS: Our results confirmed that DM induction increases renal vulnerability to the toxicity of IC and an association between DM with IC predisposes to severe AKI with reduced renal function alongside with renal hemodynamic alterations and oxidative mechanism of injury. The PT showed a renoprotective effect in DM animals subjected to damage with IC by modulating renal hemodynamics and oxidative profile, confirming a potential to modify the risk of CI-AKI when diabetes mellitus is present.

Ding, D., et al. (2016). "Vaccination against type 1 angiotensin receptor prevents streptozotocin-induced diabetic nephropathy." J Mol Med (Berl) 94(2): 207-218.
Control: N = 15 Model: N = 15

Recently, our group has developed a therapeutic hypertensive vaccine against angiotensin (Ang) II type 1 receptor (AT1R) named ATRQβ-001. To explore its potential effectiveness on streptozotocin-induced diabetic nephropathy, male Sprague Dawley rats were randomly divided into two groups: a control and a diabetic model. After 1 week, the diabetic rats were divided into four subgroups (each with 15 rats) for 14-week treatments with saline, olmesartan, ATRQβ-001, and Qβ virus-like particle (VLP), respectively. In addition to lower blood pressure, ATRQβ-001 vaccination ameliorated biochemical parameter changes of renal dysfunction, mesangial expansion, and fibrosis through inhibiting oxidative stress, macrophage infiltration, and proinflammatory factor expression. Furthermore, ATRQβ-001 vaccination suppressed renal Ang II-AT1R activation and abrogated the downregulation of angiotensin-converting enzyme 2-Ang (1-7), similar to olmesartan treatment, while no obvious feedback activation of circulating or local renin-angiotensin system (RAS) was only observed in vaccine group. In rat mesangial cells, the anti-ATR-001 antibody inhibited high glucose-induced transforming growth factor-β1 (TGF)-β1/Smad3 signal pathway. Additionally, no significant immune-mediated damage was detected in vaccinated animals. In conclusion, the ATRQβ-001 vaccine ameliorated streptozotocin-induced diabetic renal injury via modulating two RAS axes and inhibiting TGF-β1/Smad3 signal pathway, providing a novel, safe, and promising method to treat diabetic nephropathy. KEY MESSAGES: Overactivation of RAS plays a crucial role in the development of the DN. Our aim was to verify the effectiveness of ATRQβ-001 vaccine in STZ-induced DN. The ATRQβ-001 modulated two RAS axes and inhibited TGF-β1/Smad3 signal pathway. The vaccine therapy may provide a novel, safe, and promising method to treat DN.

Domon, A., et al. (2019). "Characterization of Novel Nonobese Type 2 Diabetes Rat Model with Enlarged Kidneys." J Diabetes Res 2019: 8153140.
Control: N = 4 Model: N = 4

A variety of animal models of diabetes mellitus (DM) are required to study the genetics and pathophysiology of DM. We established a novel rat strain showing nonobese type 2 diabetes with enlarged kidneys from the LEA.PET-pet congenic strain and named it Diabetes with Enlarged Kidney (DEK). The body growth of DEK affected rats was similar to that of normal rats before the development of DM but was attenuated with the deterioration of DM. There was a marked difference in the etiology of DEK by gender: DM phenotypes including polyuria, polydipsia, and hyperglycemia (nonfasting blood glucose over 300 mg/dl) were found in male rats aged over 10 weeks but not in female rats. The cumulative incidence of DM in DEK males at the age of 30 weeks was 44.8%. Oral glucose tolerance tests showed glucose intolerance and decreased insulin secretion in response to glucose loading in affected males, features which were exacerbated with age. Affected males exhibited disorganized architecture of pancreatic islets, decreased numbers of β cells, and markedly decreased expression of insulin, despite no pathological findings of hemorrhage or infiltration of inflammatory cells in the pancreatic islet. Age-related islet fibrosis appeared similar in normal and affected males. Affected males also showed enlarged kidneys with dilation of renal tubules in both the cortex and medulla, but no obvious glomerular lesions typical of diabetic nephropathy (DN) at the age of 30 weeks. Plasma levels of urea nitrogen and creatinine were normal, but hypoalbuminemia was detected. These pathophysiological features in affected males indicated that their renal function was almost maintained despite severe DM. Taken together, these findings indicate that the affected males of the DEK strain are a novel nonobese type 2 diabetes rat model useful for studying the mechanisms underlying β cell loss and identifying genetic factors protective against DN.

Dong, D., et al. (2019). "Spironolactone alleviates diabetic nephropathy through promoting autophagy in podocytes." Int Urol Nephrol 51(4): 755-764.
Control: N = 15 Model: N = 15

PURPOSE: Podocytes are terminally differentiated cells lining the Bowman's capsule. Podocytes are critical for the proper glomerular filtration barrier function. At the same time, autophagy is crucial for maintaining podocyte homeostasis and insufficient autophagy could cause podocyte loss and proteinuria that is commonly observed in diabetic nephropathy (DN). METHODS: In this study, we investigated the role of spironolactone in podocyte loss and autophagy. DN model was established in male Sprague-Dawley rats using high-fat diet and low-dose streptozotocin. The impact of spironolactone on metabolic and biochemical parameters were tested by automatic biochemical analyzer. The angiotensin converting enzyme 1 and 2 (ACE1 and ACE2) and aldosterone were examined by ELISA. We examined the kidney histology and autophagy in podocytes by histochemical staining and electron microscopy. Podocyte loss and autophagy were analyzed by anti-NPHS2 and anti-WT1 as well as anti-Beclin1 and anti-LC3B, respectively. RESULTS: Spironolacton decreased the urinary albumin excretion, lipids and fasting glucose levels, and alleviated kidney damage. Further, spironolactone increased the expression of the podocyte-specific markers WT1 and NPHS2, as well as the autophagic markers Beclin1 and LC3B ($P < 0.05$). Additionally, spironolactone partially blocked the rennin angiotensin aldosterone system (RAAS) by regulating the ACE1, ACE2 and aldosterone levels. CONCLUSIONS: In conclusion, spironolactone promoted autophagy in podocytes and further alleviated DN through partially blocking the RAAS.

Dschietzig, T. B., et al. (2015). "Relaxin-2 does not ameliorate nephropathy in an experimental model of type-1 diabetes." Kidney Blood Press Res 40(1): 77-88.
Control: N = 13 Model: N = 12

BACKGROUND/AIMS: In diabetic nephropathy (DN), the current angiotensin-II-blocking pharmacotherapy is frequently failing. For diabetic cardiomyopathy (DC), there is no specific remedy available. Relaxin-2 (Rlx) - an anti-fibrotic, anti-inflammatory, and vasoprotecting peptide – is a candidate drug for both. METHODS: Low-dose (32 μg/kg/day) and high-dose (320 μg/kg/day) Rlx were tested against vehicle (n = 20 each) and non-diabetic controls (n = 14) for 12 weeks in a model of type-1 diabetes induced in endothelial nitric oxide synthase knock-out (eNOS-KO) mice by intraperitoneal injection of streptozotocin. RESULTS: Diabetic animals showed normal plasma creatinine, markedly increased albuminuria and urinary malonyldialdehyde, elevated relative kidney weight, glomerulosclerosis, and increased glomerular size, but no relevant interstitial fibrosis. Neither dose of Rlx affected these changes although the drug was active and targeted plasma levels were achieved. Of note, we found no activation of the renal TGF-β pathway in this model. In the hearts of diabetic animals, no fibrotic alterations indicative of DC could be determined which precluded testing of the initial hypothesis. CONCLUSIONS: We investigated a model showing early DN without overt tubulointerstitial fibrosis and activation of the TGF-β-Smad-2/3 pathway. In this model, Rlx proved ineffective; however, the same may

not apply to other models and types of diabetes.

Dusabimana, T., et al. (2020). "P2Y2R contributes to the development of diabetic nephropathy by inhibiting autophagy response." Mol Metab 42: 101089.
Control: N = 8 Model: N = 10

OBJECTIVE: Diabetic nephropathy (DN) is one of the most common complications of diabetes and a critical risk factor for developing end-stage renal disease. Activation of purinergic receptors, including P2Y2R has been associated with the pathogenesis of renal diseases, such as polycystic kidney and glomerulonephritis. However, the role of P2Y2R and its precise mechanisms in DN remain unknown. We hypothesised that P2Y2R deficiency may play a protective role in DN by modulating the autophagy signalling pathway. METHODS: We used a mouse model of DN by combining a treatment of high-fat diet and streptozotocin after unilateral nephrectomy in wild-type or P2Y2R knockout mice. We measured renal functional parameter in plasma, examined renal histology, and analysed expression of autophagy regulatory proteins. RESULTS: Hyperglycaemia and ATP release were induced in wild type-DN mice and positively correlated with renal dysfunction. Conversely, P2Y2R knockout markedly attenuates albuminuria, podocyte loss, development of glomerulopathy, renal tubular injury, apoptosis and interstitial fibrosis induced by DN. These protective effects were associated with inhibition of AKT-mediated FOXO3a (forkhead box O3a) phosphorylation and induction of FOXO3a-induced autophagy gene transcription. Furthermore, inhibitory phosphorylation of ULK-1 was decreased, and the downstream Beclin-1 autophagy signalling was activated in P2Y2R deficiency. Increased SIRT-1 (sirtuin-1) and FOXO3a expression in P2Y2R deficiency also enhanced autophagy response, thereby ameliorating renal dysfunction in DN. CONCLUSIONS: P2Y2R contributes to the pathogenesis of DN by impairing autophagy and serves as a therapeutic target for treating DN.

Elseweidy, M. M., et al. (2013). "Pyridoxamine, an inhibitor of protein glycation, in relation to microalbuminuria and proinflammatory cytokines in experimental diabetic nephropathy." Exp Biol Med (Maywood) 238(8): 881-888.
Control: N = 8 Model: N = 8

Diabetic nephropathy (DN) is one of the major complications that develop as consequence of chronic and uncontrolled hyperglycaemia. Hyperglycaemia initiates various processes, one of which is protein glycation, leading to the formation of advanced glycation end products. Alteration of intracellular signalling, gene expression, release of proinflammatory molecules and free radicals are examples of such changes and they contribute to the initiation of diabetic complications. In the current manuscript, we studied the effect of pyridoxamine (PM) on protein glycation, oxidative stress, interleukin-1α (IL-1α), IL-6, C-reactive protein (CRP), gene expression of tumour necrosis factor-α (TNF-α) and transforming growth factor-β1 (TGF-β1) in relation to microalbuminuria and kidney functions in a model of alloxan-induced diabetic rats. We have observed that onset of microalbuminuria has preceded the gradual increase of blood sugar level in diabetic rats. In diabetic rats, gene expression of TNF-α and TGF-β1 recorded a gradual increase and marked increase was observed after one and two weeks of alloxan administration, in comparison with normal rats. PM induced significant decrease in kidney malondialdehyde content and the gene expression of TNF-α and TGF-β1, in addition to levels of serum glucose, fructosamine, urea, creatinine, IL-1α, IL-6, CRP and urine microalbumin. Histopathological examination of kidney tissues showed certain improvements as compared with diabetic control. In conclusion, our results may provide a supporting evidence for the therapeutic benefit of PM in DN.

Faisal Lutfi, M., et al. (2021). "Thymoquinone Lowers Blood Glucose and Reduces Oxidative Stress in a Rat Model of Diabetes." Molecules 26(8).
Control: N = 6 Model: N = 6

The aim of the present study was to assess the short-term effects of Thymoquinone (TQ) on oxidative stress, glycaemic control, and renal functions in diabetic rats. DM was induced in groups II and III with a single dose of streptozotocin (STZ), while group I received no medication (control). The rats in groups I and II were then given distilled water, while the rats in group III were given TQ at a dose of 50 mg/kg body weight/day for 4 weeks. Lipid peroxidase, nitric oxide (NO), total antioxidant capacity (TAC), glycated haemoglobin (HbA1c), lipid profiles, and renal function were assessed. Moreover, the renal tissues were used for histopathological examination. STZ increased the levels of HbA1c, lipid peroxidase, NO, and creatinine in STZ-induced diabetic rats in comparison to control rats. TAC was lower in STZ-induced diabetic rats than in the control group. Furthermore, rats treated with TQ exhibited significantly lower levels of HbA1c, lipid peroxidase, and NO than did untreated diabetic rats. TAC was higher in diabetic rats treated with TQ than in untreated diabetic rats. The histopathological results showed that

treatment with TQ greatly attenuated the effect of STZ-induced diabetic nephropathy. TQ effectively adjusts glycaemic control and reduces oxidative stress in STZ-induced diabetic rats without significant damaging effects on the renal function.

Fernandes, S. M., et al. (2016). "The role of oxidative stress in streptozotocin-induced diabetic nephropathy in rats." Arch Endocrinol Metab 60(5): 443-449.
Control: N = 6 Model: N = 6
OBJECTIVE: The objective of this study was to evaluate the role of oxidative stress in an experimental model of streptozotocin-induced diabetic nephropathy in rats. MATERIALS AND METHODS: Wistar, adult, male rats were used in the study. Animals were divided in the following groups: Citrate (control, citrate buffer 0.01M, pH 4.2 was administrated intravenously - i.v - in the caudal vein), Uninephrectomy+Citrate (left uninephrectomy-20 days before the study), DM (streptozotocin, 65 mg/kg, i.v, on the 20th day of the study), Uninephrectomy+DM. Physiological parameters (water and food intake, body weight, blood glucose, kidney weight, and relative kidney weight); renal function (creatinine clearance), urine albumin (immunodiffusion method); oxidative metabolites (urinary peroxides, thiobarbituric acid reactive substances, and thiols in renal tissue), and kidney histology were evaluated. RESULTS: Polyphagia, polydipsia, hyperglycemia, and reduced body weight were observed in diabetic rats. Renal function was reduced in diabetic groups (creatinine clearance, $p < 0.05$). Uninephrectomy potentiated urine albumin and increased kidney weight and relative kidney weight in diabetic animals ($p < 0.05$). Urinary peroxides and thiobarbituric acid reactive substances were increased, and the reduction in thiol levels demonstrated endogenous substrate consumption in diabetic groups ($p < 0.05$). The histological analysis revealed moderate lesions of diabetic nephropathy. CONCLUSION: This study confirms lipid peroxidation and intense consumption of the antioxidant defense system in diabetic rats. The association of hyperglycemia and uninephrectomy resulted in additional renal injury, demonstrating that the model is adequate for the study of diabetic nephropathy.

Franzén, S., et al. (2014). "Differences in susceptibility to develop parameters of diabetic nephropathy in four mouse strains with type 1 diabetes." Am J Physiol Renal Physiol 306(10): F1171-1178.
Control: N = 12 Model: N = 12
One-third of diabetes mellitus patients develop diabetic nephropathy, and with underlying mechanisms unknown it is imperative that diabetic animal models resemble human disease. The present study investigated the susceptibility to develop diabetic nephropathy in four commonly used and commercially available mouse strains with type 1 diabetes to determine the suitability of each strain. Type 1 diabetes was induced in C57Bl/6, NMRI, BALB/c, and 129Sv mice by alloxan, and conscious glomerular filtration rate, proteinuria, and oxidative stress levels were measured in control and diabetic animals at baseline and after 5 and 10 wk. Histological alterations were analyzed using periodic acid-Schiff staining. Diabetic C57Bl/6 displayed increased glomerular filtration rate, i.e., hyperfiltration, whereas all other parameters remained unchanged. Diabetic NMRI developed the most pronounced hyperfiltration as well as increased oxidative stress and proteinuria but without glomerular damage. Diabetic BALB/c did not develop hyperfiltration but presented with pronounced proteinuria, increased oxidative stress, and glomerular damage. Diabetic 129Sv displayed proteinuria and increased oxidative stress without glomerular hyperfiltration or damage. However, all strains displayed intrastrain correlation between oxidative stress and proteinuria. In conclusion, diabetic C57Bl/6 and NMRI both developed glomerular hyperfiltration but neither presented with histological damage, although NMRI developed low-degree proteinuria. Thus these strains may be suitable when investigating the mechanism causing hyperfiltration. Neither BALB/c nor 129Sv developed hyperfiltration although both developed pronounced proteinuria. However, only BALB/c developed detectable histological damage. Thus BALB/c may be suitable when studying the roles of proteinuria and histological alterations for the progression of diabetic nephropathy.

Gad, H. I. (2014). "Does combined peroxisome proliferator-activated receptors-agonist and pravastatin therapy attenuate the onset of diabetes-induced experimental nephropathy?" Saudi Med J 35(11): 1339-1347.
Control: N = 10 Model: N = 10
OBJECTIVES: To investigate the combined effects of rosiglitazone and pravastatin on renal functions in early streptozotocin induced diabetic nephropathy (DN). METHODS: This study was carried out at King Khalid University Hospital Animal House, Riyadh, Saudi Arabia from August 2013 to February 2014. Fifty male Wistar rats were assigned to normal control rats and diabetic rats that received saline, rosiglitazone, pravastatin, or rosiglitazone+pravastatin for 2 months. Their weight range was 230-250 gm, and age range was from 18-20 weeks. At the end of experiment, creatinine clearance, and urinary albumin to creatinine ratio (ACR) were measured. Blood samples were analyzed for transferrin, glycosylated

hemoglobin (HbA1c), lipid profile, tumor necrosis factor-alpha (TNF-α), intercellular adhesion molecule-1 (ICAM-1), and lipid peroxide. RESULTS: Rosiglitazone treatment increased creatinine clearance and plasma transferrin, and decreased urinary ACR, HbA1c, plasma TNF-α, ICAM-1, and serum lipid peroxide levels without affecting the altered lipid profile. Pravastatin treatment produced similar results and normalized the lipid alteration. The combination of rosiglitazone and pravastatin was more effective in attenuating the diabetes-induced nephropathy compared with treatment with either drug alone. CONCLUSION: The combination strategy of rosiglitazone and pravastatin may provide a potential synergistic renoprotective effect against DN by improving renal functions and reducing indices of DN.

Gargouri, M., et al. (2018). "Effects of Spirulina platensis on lipid peroxidation, antioxidant defenses, and tissue damage in kidney of alloxan-induced diabetic rats." Appl Physiol Nutr Metab 43(4): 345-354.
Control: N = 6 Model: N = 6

Chronic hyperglycemia in diabetes causes free radicals overproduction, which contributes to the development of diabetic nephropathy. In modern medicine, no satisfactory therapy is available to cure diabetes mellitus. In that context, we investigated the potential therapeutic action of spirulina-enriched diet on renal impairment and oxidative stress in diabetic rats. Diabetes was induced by a single subcutaneous injection of alloxan (120 mg·kg(-1)) in rats. Following alloxan treatment, male Wistar rats were fed daily with 5% spirulina-enriched diet or treated with insulin (0.5 IU·rat(-1)) for 3 weeks. Diabetes was associated with hyperglycemia, increase in renal oxidative parameters (lipid peroxidation, thiobarbituric-acid reactive substances, protein carbonyl and advanced oxidation protein products levels, changes in antioxidant enzyme activities), and nephropathology markers. The renal injury induced by alloxan was confirmed by histological study of the diabetic rat kidney. Treatment with spirulina or insulin significantly ameliorated renal dysfunction by reducing oxidative stress, while rats recovered normal kidney histology. Overall, this study indicates that spirulina is efficient in inhibiting hyperglycemia and oxidative stress induced by diabetes, and suggests that the administration of this alga may be helpful in the prevention of diabetic complications. This amelioration was even more pronounced than that caused by insulin injection.

Garud, M. S. and Y. A. Kulkarni (2018). "Gallic acid attenuates type I diabetic nephropathy in rats." Chem Biol Interact 282: 69-76.
Control: N = 8 Model: N = 8

Literature suggests that TGF-β1 has a central role in the progression of diabetic nephropathy and its down regulation can improve the disease condition. Oxidative stress, generation of advanced glycation end products and activation of renin angiotensin system are the connecting links between hyperglycemia and TGF-β1 over expression. Gallic acid is a phytochemical having wide range of biological activities. Gallic acid is reported to have antioxidant and advanced glycation inhibitory activity. It has also shown inhibitory effects on angiotensin converting enzyme. Gallic acid qualifies as a drug candidate to be tested in the diabetic nephropathy, one of the important complication of diabetes. Streptozotocin (55 mg/kg body weight, i.p.) induced diabetic nephropathy was used as an experimental model. Gallic acid was evaluated for its possible effect at the dose of 20 and 40 mg/kg body weight. Gallic acid treatment significantly lowered plasma levels of the creatinine and blood urea nitrogen and elevated the levels of the protein and albumin. Gallic acid also improved creatinine clearance. Determination of oxidative stress parameters showed that the oxidative stress in kidney tissues was reduced significantly in gallic acid treated animals. Results of the plasma, urine and oxidative stress parameters were also reflected in the histopathological evaluation showing improvement in kidney pathophysiology. ELISA assay for circulating TGF-β1 evaluation and immunohistochemical study for determination of kidney expression of TGF-β1 revealed that gallic acid significantly lowered both the circulating and tissue levels of TGF-β1. Results support the hypothesis that gallic acid can be effectively used in the treatment of diabetic nephropathy.

Glastras, S. J., et al. (2016). "Mouse Models of Diabetes, Obesity and Related Kidney Disease." PLoS One 11(8): e0162131.
Control: N = 11 Model: N = 11

Multiple rodent models have been used to study diabetic kidney disease (DKD). The purpose of the present study was to compare models of diabetes and obesity-induced metabolic syndrome and determine differences in renal outcomes. C57BL/6 male mice were fed either normal chow or high fat diet (HFD). At postnatal week 8, chow-fed mice were randomly assigned to low-dose streptozotocin (STZ, 55 mg/kg/day, five consecutive days) or vehicle control, whereas HFD-fed mice were given either one high-dose of STZ (100 mg/kg) or vehicle control. Intraperitoneal glucose tolerance tests were performed at Week 14, 20 and 30. Urinary albumin to creatinine ratio (ACR) and serum creatinine were measured, and renal structure was assessed using Periodic Acid Schiff (PAS) staining at Week 32. Results showed that chow-fed mice exposed to five doses of STZ resembled type 1 diabetes mellitus with a lean phenotype,

hyperglycaemia, microalbuminuria and increased serum creatinine levels. Their kidneys demonstrated moderate tubular injury with evidence of tubular dilatation and glycogenated nuclear inclusion bodies. HFD-fed mice resembled metabolic syndrome as they were obese with dyslipidaemia, insulin resistance, and significantly impaired glucose tolerance. One dose STZ, in addition to HFD, did not worsen metabolic features (including fasting glucose, non esterified fatty acid, and triglyceride levels). There were significant increases in urinary ACR and serum creatinine levels, and renal structural changes were predominantly related to interstitial vacuolation and tubular dilatation in HFD-fed mice.

Glastras, S. J., et al. (2016). "Maternal Obesity Promotes Diabetic Nephropathy in Rodent Offspring." Sci Rep 6: 27769.
Control: N = 9 Model: N = 9
Maternal obesity is known to increase the risk of obesity and diabetes in offspring. Though diabetes is a key risk factor for the development of chronic kidney disease (CKD), the relationship between maternal obesity and CKD has not been clearly defined. In this study, a mouse model of maternal obesity was employed to determine the impact of maternal obesity on development of diabetic nephropathy in offspring. Female C57BL/6 mice were fed high-fat diet (HFD) for six weeks prior to mating, during gestation and lactation. Male offspring were weaned to normal chow diet. At postnatal Week 8, offspring were randomly administered low dose streptozotocin (STZ, 55 mg/kg/day for five days) to induce diabetes. Assessment of renal damage took place at postnatal Week 32. We found that offspring of obese mothers had increased renal fibrosis, inflammation and oxidative stress. Importantly, offspring exposed to maternal obesity had increased susceptibility to renal damage when an additional insult, such as STZ-induced diabetes, was imposed. Specifically, renal inflammation and oxidative stress induced by diabetes was augmented by maternal obesity. Our findings suggest that developmental programming induced by maternal obesity has implications for renal health in offspring. Maternal obesity should be considered a risk factor for CKD.

Goru, S. K., et al. (2017). "Diminazene aceturate prevents nephropathy by increasing glomerular ACE2 and AT(2) receptor expression in a rat model of type1 diabetes." Br J Pharmacol 174(18): 3118-3130.
Control: N = 8 Model: N = 8
BACKGROUND AND PURPOSE: One of the protective actions of angiotensin converting enzyme-2 (ACE2) is the inactivation of angiotensin II. Expression and activity of ACE2 was reduced in glomeruli of diabetic patients and in animal models of diabetes. Recently the potential role of recombinant ACE2 administration in preventing diabetic nephropathy (DN) has been shown. Here we have tested the effects of the ACE2 activator, diminazene aceturate (DIZE), in a model of DN. EXPERIMENTAL APPROACH: Male Wistar rats were rendered diabetic using a single dose of streptozotocin (55 mg·kg(-1) , i.p.). After 4 weeks, diabetic animals were divided into experimental groups and treated with DIZE, at a low dose (5 mg·kg(-1) ·day(-1)), a high dose (15 mg·kg(-1) ·day(-1)) and the high dose with of the AT(2) receptor antagonist PD123319 (10 mg·kg(-1) ·day(-1)). At the end of the treatment , kidneys from all the groups were collected and processed separately for glomerular isolation, protein isolation, mRNA extraction and for immunohistochemical studies. KEY RESULTS: Treatment with DIZE restored ACE2 expression in glomeruli and increased expression of AT(2) receptors in whole kidney and isolated glomeruli of diabetic animals. DIZE administration reduced angiotensin II levels and increased angiotensin-(1-7) levels in diabetic kidney. However, PD123319 treatment reversed all these actions of DIZE. CONCLUSIONS AND IMPLICATIONS: DIZE treatment reduced diabetes-induced renal damage as shown by reduction of fibrosis and apoptosis. These protective actions of DIZE were blocked by the AT(2) receptor antagonist. Taken together, these results suggest that DIZE protected against DN through the ACE2/angiotensin-(1-7)/ AT(2) receptor axis.

Habibi, J., et al. (2019). "The combination of a neprilysin inhibitor (sacubitril) and angiotensin-II receptor blocker (valsartan) attenuates glomerular and tubular injury in the Zucker Obese rat." Cardiovasc Diabetol 18(1): 40.
Control: N = 6 Model: N = 10
OBJECTIVE: Diabetic nephropathy (DN) is characterized by glomerular and tubulointerstitial injury, proteinuria and remodeling. Here we examined whether the combination of an inhibitor of neprilysin (sacubitril), a natriuretic peptide-degrading enzyme, and an angiotensin II type 1 receptor blocker (valsartan), suppresses renal injury in a pre-clinical model of early DN more effectively than valsartan monotherapy. METHODS: Sixty-four male Zucker Obese rats (ZO) at 16 weeks of age were distributed into 4 different groups: Group 1: saline control (ZOC); Group 2: sacubitril/valsartan (sac/val) (68 mg kg(-1) day(-1); ZOSV); and Group 3: valsartan (val) (31 mg kg(-1) day(-1); ZOV). Group 4 received hydralazine, an anti-hypertensive drug (30 mg kg(-1) day(-1), ZOH). Six Zucker Lean (ZL) rats received saline (Group 5) and served as lean controls (ZLC). Drugs were administered daily for 10 weeks

by oral gavage. RESULTS: Mean arterial pressure (MAP) increased in ZOC (+ 28%), but not in ZOSV (- 4.2%), ZOV (- 3.9%) or ZOH (- 3.7%), during the 10 week-study period. ZOC were mildly hyperglycemic, hyperinsulinemic and hypercholesterolemic. ZOC exhibited proteinuria, hyperfiltration, elevated renal resistivity index (RRI), glomerular mesangial expansion and podocyte foot process flattening and effacement, reduced nephrin and podocin expression, tubulointerstitial and periarterial fibrosis, increased NOX2, NOX4 and AT(1)R expression, glomerular and tubular nitroso-oxidative stress, with associated increases in urinary markers of tubular injury. None of the drugs reduced fasting glucose or HbA1c. Hypercholesterolemia was reduced in ZOSV (- 43%) and ZOV (- 34%) ($p < 0.05$), but not in ZOH (- 13%) (ZOSV > ZOV > ZOH). Proteinuria was ameliorated in ZOSV (- 47%; $p < 0.05$) and ZOV (- 30%; $p > 0.05$), but was exacerbated in ZOH (+ 28%; $p > 0.05$) (ZOSV > ZOV > ZOH). Compared to ZOC, hyperfiltration was improved in ZOSV ($p < 0.05$ vs ZOC), but not in ZOV or ZOH. None of the drugs improved RRI. Mesangial expansion was reduced by all 3 treatments (ZOV > ZOSV > ZOH). Importantly, sac/val was more effective in improving podocyte and tubular mitochondrial ultrastructure than val or hydralazine (ZOSV > ZOV > ZOH) and this was associated with increases in nephrin and podocin gene expression in ZOSV ($p < 0.05$), but not ZOV or ZOH. Periarterial and tubulointerstitial fibrosis and nitroso-oxidative stress were reduced in all 3 treatment groups to a similar extent. Of the eight urinary proximal tubule cell injury markers examined, five were elevated in ZOC ($p < 0.05$). Clusterin and KIM-1 were reduced in ZOSV ($p < 0.05$), clusterin alone was reduced in ZOV and no markers were reduced in ZOH (ZOSV > ZOV > ZOH). CONCLUSIONS: Compared to val monotherapy, sac/val was more effective in reducing proteinuria, renal ultrastructure and tubular injury in a clinically relevant animal model of early DN. More importantly, these renoprotective effects were independent of improvements in blood pressure, glycemia and nitroso-oxidative stress. These novel findings warrant future clinical investigations designed to test whether sac/val may offer renoprotection in the setting of DN.

Han, X., et al. (2015). "Knockout of the TauT gene predisposes C57BL/6 mice to streptozotocin-induced diabetic nephropathy." PLoS One 10(1): e0117718.
Control: N = 10 Model: N = 10

Diabetic nephropathy is the leading cause of end stage renal disease in the world. Although tremendous efforts have been made, scientists have yet to identify an ideal animal model that can reproduce the characteristics of human diabetic nephropathy. In this study, we hypothesize that taurine insufficiency is a critical risk factor for development of diabetic nephropathy associated with diabetes mellitus. This hypothesis was tested in vivo in TauT heterozygous (TauT+/-) and homozygous (TauT-/-) knockout in C57BL/6 background mice. We have shown that alteration of the TauT gene (also known as SLC6A6) has a substantial effect on the susceptibility to development of extensive diabetic kidney disease in both TauT+/- and TauT-/-mouse models of diabetes. These animals developed histological changes characteristic of human diabetic nephropathy that included glomerulosclerosis, nodular lesions, arteriosclerosis, arteriolar dilation, and tubulointerstitial fibrosis. Immunohistochemical staining of molecular markers of smooth muscle actin, CD34, Ki67 and collagen IV further confirmed these observations. Our results demonstrated that both homozygous and heterozygous TauT gene deletion predispose C57BL/6 mice to develop end-stage diabetic kidney disease, which closely replicates the pathological features of diabetic nephropathy in human diabetic patients.

He, X., et al. (2019). "A new, easily generated mouse model of diabetic kidney fibrosis." Sci Rep 9(1): 12549.
Control: N = 16 Model: N = 6

Our understanding of diabetic kidney disease pathogenesis has been hampered by the lack of easily generated pre-clinical animal models that faithfully recapitulate critical features of human disease. While most standard animal models develop manifestations of early stage diabetic injury such as hyperfiltration and mesangial matrix expansion, only a select few develop key late stage features such as interstitial fibrosis and reduced glomerular filtration rate. An underlying theme in these late stage disease models has been the addition of renin-angiotensin system hyperactivation, an important contributor to human disease pathogenesis. Widespread use of these models has been limited, however, as they are either labour intensive to generate, or have been developed in the rat, preventing the use of the many powerful genetic tools developed for mice. Here we describe the Akita(+/-) Ren(+/-) mouse, a new, easily generated murine model of diabetic kidney disease that develops many features of late stage human injury, including not only hyperglycemia, hypertension, and albuminuria, but also reduced glomerular filtration rate, glomerulosclerosis, and interstitial fibrosis.

Hong, G. L., et al. (2021). "NQO1 Deficiency Aggravates Renal Injury by Dysregulating Vps34/ATG14L Complex during Autophagy Initiation in Diabetic Nephropathy." Antioxidants (Basel) 10(2).
Control: N = 5 Model: N = 5

Diabetic nephropathy (DN) is one of the causes of end-stage renal failure, featuring renal fibrosis. However, autophagy, a vital process for intracellular homeostasis, can counteract renal fibrosis. Moreover, NAD(P)H: quinone dehydrogenase 1 (NQO1) modulates the ratios of reduced/oxidized nicotinamide nucleotides, exerting a cytoprotective function. Here, to examine the role of NQO1 genes in DN progression, the levels of autophagy-related proteins and pro-fibrotic markers were assessed in silencing or overexpression of NQO1 in human proximal tubular cells (HK2), and C57BL/6 (wild-type) and Nqo1 knockout (KO) mice injected to streptozotocin (50 mg/kg). NQO1 deficiency impaired the autophagy process by suppressing basal expression of ClassIII PI 3-kinase (Vps34) and autophagy-related (ATG)14L and inducing the expressions of transforming growth factor beta (TGF-β1), Smad3, and matrix metallopeptidase9 (MMP9) in high-glucose (HG) -treated HK2 cells. Meanwhile, NQO1 overexpression increased the expression of Vps34 and ATG14L, while, reducing TGF-β1, Smad3 and MMP9 expression. In vivo, the expression of Vps34 and ATG14L were suppressed in Nqo1 KO mice indicating aggravated glomerular changes and interstitial fibrosis. Therefore, NQO1 deficiency dysregulated autophagy initiation in HK2 cells, with consequent worsened renal cell damage under HG condition. Moreover, STZ-treated Nqo1 KO mice showed that NQO1 deficiency aggravated renal fibrosis by dysregulating autophagy.

Hu, S. J., et al. (2018). "Therapeutic Role of Tangshenkang Granule () in Rat Model with Diabetic Nephropathy." Chin J Integr Med 24(8): 600-605.
Control: N = 12 Model: N = 10

OBJECTIVE: To evaluate the renal protective effect of Tangshenkang Granule () in a rat model of diabetic nephropathy (DN). METHODS: Forty male Sprague-Dawley rats were randomly divided into control, DN, Tangshenkang and benazepril groups. DN model was established in the rats of DN, Tangshenkang and benazepril groups. Tangshenkang Granule solution and benazepril hydrochloride solution were intragastrically administered daily to the rats in the Tangshenkang and benazepril groups for 8 weeks, respectively. Urinary albumin and creatinine were detected. The albumin/creatinine (ACR) was calculated in addition to 24 h urinary protein (24-h UPr), serum creatinine (Scr), blood urea nitrogen (BUN), total cholesterol (TC), triglyceride (TG), low-density lipoprotein (LDL), high-density lipoprotein (HDL), and creatinine clearance rate (Ccr). Right kidneys were harvested for pathological observation using periodic acid-silver methenamine-Masson staining. The average glomerular diameter (DG), average glomerular (AG) and mesangial areas (AM) were measured. The thickness of glomerular basement membrane (TGBM) was detected using transmission electron microscope. RESULTS: Compared with rats in the control group, rats in the DN group showed significantly decreased body weight, increased hypertrophy index, 24-h urinary volume, 24-h UPr, ACR, Scr, BUN, Ccr, blood lipids as well as renal pathological indices including DG, AG, AM, AM/AG and TGBM ($P < 0.05$). Compared with the DN group, the weights of rats in the Tangshenkang and benazepril groups were significantly increased, and the renal hypertrophy indices were significantly decreased ($P < 0.05$). The 24-h urinary volumes, ACR, 24-h UPr, Scr, BUN, Ccr, LDL, DG, AG, AM and TGBM were obviously decreased ($P < 0.05$). Compared with the benazepril group, the Tangshenkang group showed significantly decreased levels of ACR, 24-h UPr, AG and AM ($P < 0.05$). CONCLUSIONS: Tangshenkang Granule decreased the urinary protein, attenuated the high glomerular filtration rate and improved lipid metabolism in DN rats, and prevented further injury induced by diabetic nephropathy.

Huang, C., et al. (2018). "The KCa3.1 blocker TRAM34 reverses renal damage in a mouse model of established diabetic nephropathy." PLoS One 13(2): e0192800.
Control: N = 7 Model: N = 7

Despite optimal control of hyperglycaemia, hypertension, and dyslipidaemia, the number of patients with diabetic nephropathy (DN) continues to grow. Strategies to target various signaling pathways to prevent DN have been intensively investigated in animal models and many have been proved to be promising. However, targeting these pathways once kidney disease is established, remain unsatisfactory. The clinical scenario is that patients with diabetes mellitus often present with established kidney damage and need effective treatments to repair and reverse the kidney damage. In this studies, eNOS-/- mice were administered with streptozotocin to induce diabetes. At 24 weeks, at which time we have previously demonstrated albuminuria and pathological changes of diabetic nephropathy, mice were randomised to receive TRAM34 subcutaneously, a highly selective inhibitor of potassium channel KCa3.1 or DMSO

(vehicle) for a further 14 weeks. Albuminuria was assessed, inflammatory markers (CD68, F4/80) and extracellular matrix deposition (type I collagen and fibronectin) in the kidneys were examined. The results clearly demonstrate that TRAM34 reduced albuminuria, decreased inflammatory markers and reversed extracellular matrix deposition in kidneys via inhibition of the TGF-β1 signaling pathway. These results indicate that KCa3.1 blockade effectively reverses established diabetic nephropathy in this rodent model and provides a basis for progressing to human studies.

Huang, H., et al. (2017). "Protective effects of allicin on streptozotocin-induced diabetic nephropathy in rats." J Sci Food Agric 97(4): 1359-1366.
Control: N = 10 Model: N = 12
BACKGROUND: Studies in animal models have shown that allicin, a major biologically active component of garlic, can play a role in the prevention of tissue fibrosis in the liver, lung and heart, mainly related to the inhibition of fibroblast proliferation, fibrogenic cytokine secretion and extracellular matrix synthesis. This study aimed to investigate the protective effects of allicin on renal damage in streptozotocin (STZ)-induced diabetic rats. STZ-induced diabetic rats were administered allicin (15, 30 and 45 mg · kg(-1) · day(-1)) via daily intra-gastric gavage for 12 weeks. The levels of fasting blood glucose (FBG), blood urea nitrogen (BUN), serum creatinine (sCr), lipid and 24 h urine albumin excretion (UAE) were measured at the end of weeks 4, 8 and 12. The renal histopathology and the expression levels of collagen I, transforming growth factor β1 (TGF-β1) and phosphorylated extracellular signal-regulated kinase 1/2 (p-ERK1/2) were measured using immunohistochemistry and/or western blotting. RESULTS: In 12 week STZ-induced diabetic rats, severe hyperglycemia and albuminuria were markedly developed. Treatment with allicin for 12 weeks ameliorated diabetes-induced morphological alterations of the kidney and decreased FBG, BUN, sCr, triglyceride (TG) and 24 h UAE in diabetic rats. The expression levels of collagen I, TGF-β1 and p-ERK1/2 were significantly decreased by allicin treatment. CONCLUSION: These results suggested that allicin may play a protective role in diabetic nephropathy via the TGF-β1/ERK pathway in diabetic rats. © 2016 Society of Chemical Industry.

Ibrahim, D. S. and M. A. Abd El-Maksoud (2015). "Effect of strawberry (Fragaria × ananassa) leaf extract on diabetic nephropathy in rats." Int J Exp Pathol 96(2): 87-93.
Control: N = 6 Model: N = 6
Diabetic nephropathy is a clinical syndrome characterized by albuminuria, hypertension and progressive renal insufficiency. The aim of this study was to investigate the effect of strawberry (Fragaria × ananassa) leaf extract on diabetic nephropathy in rats. Streptozotocin (STZ) diabetic rats were orally treated with three doses (50, 100 and 200 mg/kg) of strawberry leaf extract for 30 days. Nephropathy biomarkers in plasma and kidney were examined at the end of the experiment. The three doses of strawberry leaf extract significantly decreased the levels of blood glucose, urea nitrogen, plasma creatinine, kidney injury molecule (Kim)-1, renal malondialdehyde (MDA), tumour necrosis factor alpha (TNF-α), interleukin (IL)- 6 and caspase-3 in diabetic rats. Meanwhile, the levels of plasma insulin, albumin, uric acid, renal catalase (CAT), superoxide dismutase (SOD) and vascular endothelial growth factor A (VEGF-A) were significantly elevated in diabetic rats treated with strawberry leaf extract. These results indicate the role of strawberry leaves extract as anti-diabetic, antioxidant, anti-inflammatory and anti-apoptosis in diabetic nephropathy.

Ibrahim, Z. S., et al. (2016). "Renoprotective effect of curcumin against the combined oxidative stress of diabetes and nicotine in rats." Mol Med Rep 13(4): 3017-3026.
Control: N = 8 Model: N = 8
The progression of diabetic nephropathy (DN) is accelerated by smoking. The current study investigated the ability of curcumin to protect the kidneys against damage from oxidative stress induced by diabetes mellitus (DM) and nicotine (NC). A total of 24 male Wistar rats were divided into four groups of six rats each. DM was induced by a single intraperitoneal injection of streptozotocin 60 mg/kg body weight. DM rats were treated with or without NC in the absence or presence of curcumin for 8 weeks. As compared with the controls, DM rats exhibited reduced serum levels of high density lipoprotein, superoxide dismutase and glutathione peroxidase, and decreased renal mRNA expression levels of synaptopodin, connexin 43 and erythropoietin (EPO), which were further suppressed by NC and restored to normal levels by curcumin treatment. Additionally, DM rats exhibited increases in their lipid profiles (cholesterol, triacylglycerol and phospholipids), oxidative markers (malondialdehyde, γ-glutamyltranspeptidase and nitric oxide), kidney function markers (urea and creatinine) and the mRNA expression levels of vimentin, desmin, SREBP-1, iNOS and TGF-β1. These effects were further enhanced by NC, but counteracted by curcumin treatment. Kidneys from DM rats displayed glomerular hypertrophy, sclerosis and tubulo-interstitial changes represented by tubular lipid deposition, interstitial mononuclear

cell infiltration and fibroplasia. Pancreatic islets exhibited cellular vacuolation, morphological irregularity and damaged or reduced in size β-cells. These renal and pancreatic changes became more severe following NC treatment and were ameliorated by curcumin. Therefore, NC-induced DN progression may predominantly operate by increasing oxidative stress, reducing the levels of antioxidants, suppressing EPO levels, and causing perturbations to gap junction and podocyte structure. Curcumin may ameliorate the damaging effects of DM and NC on the kidney through normalization of the mRNA expression levels of several genes important in the progression of DN.

Itano, S., et al. (2020). "Non-purine selective xanthine oxidase inhibitor ameliorates glomerular endothelial injury in Ins(Akita) diabetic mice." Am J Physiol Renal Physiol 319(5): F765-f772.
Control: N = 6 Model: N = 6

Endothelial dysfunction represents a predominant early feature of diabetes, rendering patients with diabetes prone to renal complications, e.g., proteinuria. Recent studies have indicated a possible role for xanthine oxidase (XO) in the pathogenesis of vascular dysfunctions associated with diabetes. In the present study, we investigated the contribution of XO activation on the progression of diabetic nephropathy in a mouse model using selective XO inhibitors. Male Ins2(Akita) heterozygous mice were used with wild-type mice as controls. Akita mice were treated with topiroxostat (Topi) or vehicle for 4 wk. Serum uric acid levels were significantly reduced in Akita + Topi mice compared with Akita + vehicle mice. The Akita + Topi group had a significant reduction in urinary albumin excretion compared with the Akita + vehicle group. Mesangial expansion, glomerular collagen type IV deposition, and glomerular endothelial injury (assessed by lectin staining and transmission electron microscopy) were considerably reduced in the Akita + topi group compared with the Akita + vehicle group. Furthermore, glomerular permeability was significantly higher in the Akita + vehicle group compared with the wild-type group. These changes were reduced with the administration of Topi. We conclude that XO inhibitors preserve glomerular endothelial functions and rescue compromised glomerular permeability, suggesting that XO activation plays a vital role in the pathogenesis of diabetic nephropathy.

Jeong, B. Y., et al. (2016). "Novel Plasminogen Activator Inhibitor-1 Inhibitors Prevent Diabetic Kidney Injury in a Mouse Model." PLoS One 11(6): e0157012.
Control: N = 7 Model: N = 7

Diabetic nephropathy is the leading cause of end-stage renal disease worldwide, but no effective therapeutic strategy is available. Because plasminogen activator inhibitor-1 (PAI-1) is increasingly recognized as a key factor in extracellular matrix (ECM) accumulation in diabetic nephropathy, this study examined the renoprotective effects of TM5275 and TM5441, two novel orally active PAI-1 inhibitors that do not trigger bleeding episodes, in streptozotocin (STZ)-induced diabetic mice. TM5275 (50 mg/kg) and TM5441 (10 mg/kg) were administered orally for 16 weeks to STZ-induced diabetic and age-matched control mice. Relative to the control mice, the diabetic mice showed significantly increased ($p < 0.05$) plasma glucose and creatinine levels, urinary albumin excretion, kidney-to-bodyweight ratios, glomerular volume, and fractional mesangial area. Markers of fibrosis and inflammation along with PAI-1 were also upregulated in the kidney of diabetic mice, and treatment with TM5275 and TM5441 effectively inhibited albuminuria, mesangial expansion, ECM accumulation, and macrophage infiltration in diabetic kidneys. Furthermore, in mouse proximal tubular epithelial (mProx24) cells, both TM5275 and TM5441 effectively inhibited PAI-1-induced mRNA expression of fibrosis and inflammation markers and also reversed PAI-1-induced inhibition of plasmin activity, which confirmed the efficacy of the TM compounds as PAI-1 inhibitors. These data suggest that TM compounds could be used to prevent diabetic kidney injury.

Kim, J. E., et al. (2013). "Celastrol, an NF-κB inhibitor, improves insulin resistance and attenuates renal injury in db/db mice." PLoS One 8(4): e62068.
Control: N = 8 Model: N = 8

The NF-κB pathway plays an important role in chronic inflammatory and autoimmune diseases. Recently, NF-κB has also been suggested as an important mechanism linking obesity, inflammation, and metabolic disorders. However, there is no current evidence regarding the mechanism of action of NF-κB inhibition in insulin resistance and diabetic nephropathy in type 2 diabetic animal models. We investigated the effects of the NF-κB inhibitor celastrol in db/db mice. The treatment with celastrol for 2 months significantly lowered fasting plasma glucose (FPG), HbA1C and homeostasis model assessment index (HOMA-IR) levels. Celastrol also exhibited significant decreases in body weight, kidney/body weight and adiposity. Celastrol reduced insulin resistance and lipid abnormalities and led to higher plasma adiponectin levels. Celastrol treatment also significantly mitigated lipid accumulation and oxidative stress

in organs including the kidney, liver and adipose tissue. The treated group also exhibited significantly lower creatinine levels and urinary albumin excretion was markedly reduced. Celastrol treatment significantly lowered mesangial expansion and suppressed type IV collagen, PAI-1 and TGFβ1 expressions in renal tissues. Celastrol also improved abnormal lipid metabolism, oxidative stress and proinflammatory cytokine activity in the kidney. In cultured podocytes, celastrol treatment abolished saturated fatty acid-induced proinflammatory cytokine synthesis. Taken together, celastrol treatment not only improved insulin resistance, glycemic control and oxidative stress, but also improved renal functional and structural changes through both metabolic and anti-inflammatory effects in the kidney. These results suggest that targeted therapy for NF-κB may be a useful new therapeutic approach for the management of type II diabetes and diabetic nephropathy.

Klein, J., et al. (2016). "Urinary peptidomics provides a noninvasive humanized readout of diabetic nephropathy in mice." Kidney Int 90(5): 1045-1055.
Control: N = 9 Model: N = 11

Nephropathy is among the most frequent complications of diabetes and the leading cause of end-stage renal disease. Despite the success of novel drugs in animal models, the majority of the subsequent clinical trials employing those drugs targeting diabetic nephropathy failed. This lack of translational value may in part be due to an inadequate comparability of human disease and animal models that often capture only a few aspects of disease. Here we overcome this limitation by developing a multimolecular noninvasive humanized readout of diabetic nephropathy based on urinary peptidomics. The disease-modified urinary peptides of 2 type 2 diabetic nephropathy mouse models were identified and compared with previously validated urinary peptide markers of diabetic nephropathy in humans to generate a classifier composed of 21 ortholog peptides. This classifier predicted the response to disease and treatment with inhibitors of the renin-angiotensin system in mice. The humanized classifier was significantly correlated with glomerular lesions. Using a human type 2 diabetic validation cohort of 207 patients, the classifier also distinguished between patients with and without diabetic nephropathy, and their response to renin-angiotensin system inhibition. Thus, a combination of multiple molecular features common to both human and murine disease could provide a significant change in translational drug discovery research in type 2 diabetic nephropathy.

Ladeira, L. C. M., et al. (2021). "Green tea infusion prevents diabetic nephropathy aggravation in recent-onset type 1 diabetes regardless of glycemic control." J Ethnopharmacol 274: 114032.
Control: N = 6 Model: N = 6

ETHNOPHARMACOLOGICAL RELEVANCE: Green tea, traditionally used as antidiabetic medicine, positively affects the diabetic nephropathy. It was assumed that these beneficial effects were due to the hypoglycemiant capacity of the tea, wich reduces the glycemic overload and, consequently, the advanced glycation end products rate and oxidative damage. However, these results are still controversial, since tea is not always able to exert a hypoglycemic action, as demonstrated by previous studies. AIM: Investigate if green tea infusion can generate positive outcomes for the kidney independently of glycemic control, using a model of severe type 1 diabetes. MATERIAL AND METHODS: We treated streptozotocin type 1 diabetic young rats with 100 mg/kg of green tea, daily, for 42 days, and evaluated the serum and tissue markers for stress and function. We also analyzed the ion dynamics in the organ and the morphological alterations promoted by diabetes and green tea treatment. Besides, we analyzed, by an in silico approach, the interactions of the green tea main catechins with the proteins expressed in the kidney. RESULTS: Our findings reveal that the components of green tea can interact with the proteins participating in cell signaling pathways that regulate energy metabolism, including glucose and glycogen synthesis, glucose reabsorption, hypoxia management, and cell death by apoptosis. Such interaction reduces glycogen accumulation in the organ, and protects the DNA. These results also reflect in a preserved glomerulus morphology, with improvement in pathological features, and suggesting a prevention of kidney function impairment. CONCLUSION: Our results show that such benefits are achieved regardless of the blood glucose status, and are not dependent on the reduction of hyperglycemia.

Lezcano, E. J., et al. (2014). "Caloric restriction or telmisartan control dyslipidemia and nephropathy in obese diabetic Zücker rats." Diabetol Metab Syndr 6(1): 10.
Control: N = 5 Model: N = 8

BACKGROUND: The obese Zücker diabetic fatty male rat (ZDF:Gmi™-fa) is an animal model of type II diabetes associated with obesity and related metabolic disturbances like dyslipidaemia and diabetic nephropathy. In addition, diabetic dyslipidaemia has been linked to vascular and glomerular damage too. Dietary fat restriction is a current strategy to tackle obesity and, telmisartan, as a renoprotective agent, may mediate cholesterol efflux by activating PPARγ. To test the hypothesis that both therapeutical alternatives may influence dyslipidaemia and nephropathy in the ZDF rat, we studied their effect on development of diabetes. METHODS: Male Zücker Diabetic Fatty (ZDF) rats received a low-calorie diet, vehicle or telmisartan for 9 weeks. Blood samples were obtained for analyses of lipids and lipoproteins, LDL-oxidisability, HDL structural and functional properties. Urinalysis was carried out to estimate albumin loss. At the end of the experimental period, rats were sacrificed, liver extracted and APOA1 mRNA quantified. RESULTS: Results indicated that low-calorie diet and telmisartan can slower the onset of overt hyperglycaemia and renal damage assessed as albuminuria. Both interventions decreased the oxidative susceptibility of LDL and hepatic APOA1 mRNA expression but only dietary restriction lowered hyperlipidaemia. CONCLUSION: Either a dietary or pharmacologic interventions with telmisartan have important beneficial effects in terms of LDL oxidative susceptibility and progression of albuminuria in obesity related type II diabetes.

Li, H. Y., et al. (2017). "Blocking lysophosphatidic acid receptor 1 signaling inhibits diabetic nephropathy in db/db mice." Kidney Int 91(6): 1362-1373.
Control: N = 10 Model: N = 10

Lysophosphatidic acid (LPA) is known to regulate various biological responses by binding to LPA receptors. The serum level of LPA is elevated in diabetes, but the involvement of LPA in the development of diabetes and its complications remains unknown. Therefore, we studied LPA signaling in diabetic nephropathy and the molecular mechanisms involved. The expression of autotaxin, an LPA synthesis enzyme, and LPA receptor 1 was significantly increased in both mesangial cells (SV40 MES13) maintained in high-glucose media and the kidney cortex of diabetic db/db mice. Increased urinary albumin excretion, increased glomerular tuft area and volume, and mesangial matrix expansion were observed in db/db mice and reduced by treatment with ki16425, a LPA receptor 1/3 antagonist. Transforming growth factor (TGF)β expression and Smad-2/3 phosphorylation were upregulated in SV40 MES13 cells by LPA stimulation or in the kidney cortex of db/db mice, and this was blocked by ki16425 treatment. LPA receptor 1 siRNA treatment inhibited LPA-induced TGFβ expression, whereas cells overexpressing LPA receptor 1 showed enhanced LPA-induced TGFβ expression. LPA treatment of SV40 MES13 cells increased phosphorylated glycogen synthase kinase (GSK)3β at Ser9 and induced translocation of sterol regulatory element-binding protein (SREBP)1 into the nucleus. Blocking GSK3β phosphorylation inhibited SREBP1 activation and consequently blocked LPA-induced TGFβ expression in SV40 MES13 cells. Phosphorylated GSK3β and nuclear SREBP1 accumulation were increased in the kidney cortex of db/db mice and ki16425 treatment blocked these pathways. Thus, LPA receptor 1 signaling increased TGFβ expression via GSK3β phosphorylation and SREBP1 activation, contributing to the development of diabetic nephropathy.

Li, M., et al. (2013). "GC/TOFMS analysis of metabolites in serum and urine reveals metabolic perturbation of TCA cycle in db/db mice involved in diabetic nephropathy." Am J Physiol Renal Physiol 304(11): F1317-1324.
Control: N = 8 Model: N = 8

Early diagnosis of diabetic nephropathy (DN) is difficult although it is of crucial importance to prevent its development. To probe potential markers and the underlying mechanism of DN, an animal model of DN, the db/db mice, was used and serum and urine metabolites were profiled using gas chromatography/time-of-flight mass spectrometry. Metabolic patterns were evaluated based on serum and urine data. Principal component analysis of the data revealed an obvious metabonomic difference between db/db mice and controls, and db/db mice showed distinctly different metabolic patterns during the progression from diabetes to early, medium, and later DN. The identified metabolites discriminating between db/db mice and controls suggested that db/db mice have perturbations in the tricarboxylic acid cycle (TCA, citrate, malate, succinate, and aconitate), lipid metabolism, glycolysis, and amino acid turnover. The db/db mice were characterized by acidic urine, high TCA intermediates in serum at week 6 and a sharp decline thereafter, and gradual elevation of free fatty acids in the serum. The sharp drop of serum TCA intermediates from week 6 to 8 indicated the downregulated glycolysis and insulin resistance. However, urinary TCA intermediates did not decrease in parallel with those in the serum from week 6 to 10, and an increased portion of TCA intermediates in the serum was excreted into the urine at 8, 10, and 12 wk than at 6 wk, indicating kidney dysfunction occurred. The relative abundances of TCA intermediates in urine relative to those in serum were suggested as an index of renal damage.

Li, X., et al. (2017). "Nephrin loss is reduced by grape seed proanthocyanidins in the experimental

diabetic nephropathy rat model." Mol Med Rep 16(6): 9393-9400.
Control: N = 10 Model: N = 8

Diabetic nephropathy (DN) is one of the major causes of end-stage renal failure. Grape seed proanthocyanidin extracts (GSPE) are known to act as antioxidants. The current study aimed to determine the effects of GSPE on the streptozotocin (STZ)-induced diabetic rat model and to explore the underlying mechanism of its action. Wistar rats were induced into a diabetic state by injection of STZ and were treated with 250 mg·kg-1·day-1 GSPE for 24 weeks. Kidney samples were collected for observation of renal pathological changes by light microscope (periodic acid-Schiff staining) and electron microscopy. Reverse transcription-polymerase chain reaction, western blotting, and immunohistochemical staining were used to detect the mRNA and protein expression of the receptor for advanced glycation end-products (RAGE), nephrin and podocin. The results indicated that diabetic rats treated with GSPE had markedly reduced Ccr, urinary albumin excretion, ratio of kidney weight to body weight, AGEs and ECM accumulation (P<0.01) compared with that in the diabetic rats. GSPE treatment can also reverse the renal pathological damage in diabetic rats. Further results indicated that GSPE treatment significantly decreased the RAGE expression level (P<0.01), and significantly increased the expression level of nephrin in the kidney and glomeruli of diabetic rats (P<0.01). However, no significant differences were identified in the expression of podocin following GSPE treatment (P>0.05). In conclusion, the results demonstrated that GSPE exerts a reno-protective effect by decreasing urinary albumin excretion and reversing renal pathological damage in diabetic rats. The underlying mechanism of GSPE activity is associated with the decreased expression of the AGEs/RAGE axis and the increased expression of nephrin in diabetic rats.

Li, Z., et al. (2020). "The sodium-glucose cotransporter 2 inhibitor tofogliflozin prevents diabetic kidney disease progression in type 2 diabetic mice." FEBS Open Bio 10(12): 2761-2770.
Control: N = 9 Model: N = 15

Trials on cardiovascular and renal outcomes in patients with type 2 diabetes have consistently demonstrated that sodium-glucose cotransporter 2 (SGLT2) inhibitors reduce the risk of diabetic kidney disease (DKD) progression. However, their renal protective mechanisms have yet to be completely understood and the effect on albuminuria reduction in animal models is controversial. We investigated these issues using KK and KK-A(y) mice as a control (CTRL) and as a model for type 2 diabetes (DKD), respectively. KK-A(y) mice were treated with 0.015% tofogliflozin, which is an SGLT2 inhibitor, starting at seven weeks of age for eight weeks. Compared with the CTRL mice, the DKD mice had higher HbA1c levels and albuminuria. Although tofogliflozin treatment significantly lowered HbA1c levels, it did not reverse albuminuria. Tofogliflozin treatment enhanced damage in both the glomerular (i.e., enlarged mesangial area, increased foot process effacement rate, and decreased number of WT-1-positive cells) and tubulointerstitial (increased protein levels of KIM-1 and MCP-1, increased number of macrophages, and abnormal mitochondrial morphology) areas. Our results suggest that tofogliflozin may prevent glomerular and tubulointerstitial damage, partly by ameliorating hyperglycemia, renal inflammation, and abnormal mitochondrial morphology.

Lian, H., et al. (2019). "Malignant fibrous histiocytoma amplified sequence 1 alleviates inflammation and renal fibrosis in diabetic nephropathy by inhibiting TLR4." Biosci Rep 39(11).
Control: N = 6 Model: N = 10

BACKGROUND: Diabetic nephropathy (DN) is the most common complication of diabetes mellitus (DM). The signal pathway and molecular mechanism of renal fibrosis are not fully understood. In the present study, we aimed to explore the function of malignant fibrous histiocytoma amplified sequence 1 (MFHAS1) in DN. METHOD: Mouse mesangial cells (MMCs) were treated with low glucose (LG) or high glucose (HG). TAK242 or short hairpin TLR4 (shTLR4) were employed to down-regulate Toll-like receptor 4 (TLR4). The effect of MFHAS1 knockdown or overexpression on fibrosis-related factors, inflammatory factors and TLR4 in MMCs were examined after transfecting with short hairpin RNA (shRNA) or MFHAS1 overexpressed plasmid, respectively. The expression levels of MFHAS1, inflammatory factors, fibrosis factors and TLR4 in db/db or streptozotocin (STZ) mice tissues and MMCs were examined by quantitative real-time polymerase chain reaction (qRT-PCR) and Western blot. The effect of MFHAS1 overexpression in vivo was also evaluated. RESULTS: The expression of MFHAS1 in db/db or STZ mice and HG-treated MMCs were significantly increased compared with normal control mice and LG-treated MMCs. Overexpression of MFHAS1 inhibited the expression of inflammatory and fibrotic factors, while knockdown of MFHAS1 promoted them. MFHAS1 suppressed the activation of TLR4 pathway via inhibiting the expression of TLR4, and then alleviating inflammation and fibrosis in DN. MFHAS1 overexpression in vivo improved the symptoms of STZ-induced DN mice. CONCLUSION: The current study demonstrated that MFHAS1 relieved inflammation and renal fibrosis in DN mice via inhibiting TLR4. The results revealed that the MFHAS1 may be a molecular target in DN therapy.

Liu, H. W., et al. (2019). "Exercise training upregulates SIRT1 to attenuate inflammation and metabolic dysfunction in kidney and liver of diabetic db/db mice." Nutr Metab (Lond) 16: 22.
Control: N = 8 Model: N = 8

BACKGROUND: Chronic inflammation and metabolic dysregulation may eventually cause tissue damage in obesity-related diseases such as type 2 diabetes. The effects of SIRT1 on integration of metabolism and inflammation may provide a therapeutic target for treatment of obesity-related diseases. We examined the underlying mechanism of moderate intensity aerobic exercise on kidney and liver in obese diabetic db/db mice, mainly focusing on inflammation and metabolic dysfunction. METHODS: Functional and morphological alterations and metabolic and inflammatory signaling were examined in type 2 diabetic db/db mice with or without exercise training (5.2 m/min, 1 h/day, and 5 days/week for a total of 8 weeks). RESULTS: Exercise training prevented weight gain in db/db + Ex mice, but it did not reduce glucose and insulin levels. Exercise lowered serum creatinine, urea, and triglyceride levels and hepatic AST and ALT activity in db/db + Ex mice. Reduced kidney size and morphological alterations including decreased glomerular cross-sectional area and hepatic macrovesicles were observed in db/db + Ex mice compared with untrained db/db mice. Mechanistically, preventing loss of SIRT1 through exercise was linked to reduced acetylation of NF-κB in kidney and liver of db/db + Ex mice. Exercise increased citrate synthase and mitochondrial complex I activity, subunits of mitochondrial complexes (I, II, and V) and PGC1α at protein level in kidney of db/db + Ex mice compared with non-exercise db/db mice. Changes in enzyme activity and subunits of mitochondrial complexes were not observed in liver among three groups. CONCLUSION: Exercise-induced upregulation of SIRT1 attenuates inflammation and metabolic dysfunction, thereby alleviating the progression of diabetic nephropathy and hepatic steatosis in type 2 diabetes mellitus.

Liu, W., et al. (2013). "The expression of intermediate filament protein nestin and its association with cyclin-dependent kinase 5 in the glomeruli of rats with diabetic nephropathy." Am J Med Sci 345(6): 470-477.
Control: N = 6 Model: N = 6

BACKGROUND: Podocyte injury plays a crucial role in the development of diabetic nephropathy (DN), but its underlying mechanism remains poorly understood. Emerging evidences suggest that the cytoskeleton disruption is related to podocyte injury. The aim of this study was to investigate whether nestin, a cytoskeleton-associated intermediate filament protein, is involved in the development of DN. METHODS: Rat diabetes was induced by intraperitoneal injection of streptozotocin. The renal histological changes were investigated by light microscopy and transmission electron microscopy. The location of nestin and vimentin in renal tissues was observed by immunohistochemistry. The protein or messenger RNA levels of nestin and cyclin-dependent kinase 5 (Cdk5) were detected by Western blot and real-time polymerase chain reaction. The relationship between nestin and vimentin was detected by co-immunoprecipitation. RESULTS: Compared with controls, diabetic rats showed significant characteristics of renal damage. The expression of nestin and vimentin in the glomeruli was increased at the early stage of diabetes, which then gradually decreased. Co-immunoprecipitation assays demonstrated that nestin disassembled with vimentin in diabetic rats. The expression of Cdk5 was increased in a time-dependent manner in diabetic rats. The degree of albuminuria in diabetic rats was negatively correlated with nestin and positively correlated with Cdk5. Roscovitine, a Cdk5 inhibitor, reduced the degradation of nestin. Moreover, podocyte injuries were significantly ameliorated by treatment with roscovitine. CONCLUSIONS: The intermediate filament protein nestin is associated with development of DN. Blockage of Cdk5 increases the level of nestin and attenuates renal damage, which would provide a useful target for DN therapy.

Lopez-Parra, V., et al. (2012). "Fcγ receptor deficiency attenuates diabetic nephropathy." J Am Soc Nephrol 23(9): 1518-1527.
Control: N = 7 Model: N = 12

Among patients with diabetes, increased production of immunoglobulins against proteins modified by diabetes is associated with proteinuria and cardiovascular risk, suggesting that immune mechanisms may contribute to the development of diabetes complications, such as nephropathy. We investigated the contribution of IgG Fcγ receptors to diabetic renal injury in hyperglycemic, hypercholesterolemic mice. We used streptozotocin to induce diabetes in apolipoprotein E-deficient mice and in mice deficient in both apolipoprotein E and γ-chain, the common subunit of activating Fcγ receptors. After 15 weeks, the mice lacking Fcγ receptors had significantly less albuminuria and renal hypertrophy, despite similar degrees of hyperglycemia and hypercholesterolemia, immunoglobulin production, and glomerular immune deposits. Moreover, diabetic Fcγ receptor-deficient mice had less mesangial matrix expansion, inflammatory cell infiltration, and collagen and α-smooth muscle actin content in their kidneys. Accordingly, expression of genes involved in leukocyte infiltration, fibrosis, and oxidative stress was significantly reduced in diabetic

kidneys and in mesangial cells cultured from Fcγ receptor-deficient mice. In summary, preventing the activation of Fcγ receptors alleviates renal hypertrophy, inflammation, and fibrosis in hypercholesterolemic mice with diabetes, suggesting that modulating Fcγ receptor signaling may be renoprotective in diabetic nephropathy.

Ma, K. L., et al. (2014). "Establishment of an inflamed animal model of diabetic nephropathy." Int J Biol Sci 10(2): 149-159.
Control: N = 10 Model: N = 10

AIMS: Inflammatory stress plays a crucial role in the progression of diabetic nephropathy (DN). This study aimed to establish a novel inflamed animal model of DN and to evaluate its significance in DN. METHODS: Nondiabetic db/m mice and diabetic db/db mice were randomly divided into four groups: db/m, db/m+casein, db/db, and db/db+casein for eight weeks. Casein was subcutaneously injected to induce chronic inflammation. Body weight and albumin to creatinine ratio (ACR) in the urine were measured every week. The plasma levels of serum amyloid protein A (SAA) and tumour necrotic factor-α (TNF-α) were determined with the enzyme-linked immunosorbent assay. The morphological changes to the renal pathology and ultra-microstructures were checked by pathological staining and electron microscopy. Immunofluorescent staining and Western blotting were used to determine the protein expression of podocyte-specific molecules and inflammatory cytokines in kidneys. RESULTS: ACR, plasma levels of SAA and TNF-α, protein expression of inflammatory cytokines, mesangial expansion, collagen accumulation, and foot process effacement in kidneys of casein-injected db/db mice were significantly increased compared with the db/db mice. Casein injection markedly decreased the protein expression of Wilms' tumor-1 and nephrin in kidneys of db/db mice, which are specific podocyte biomarkers, suggesting that chronic inflammation accelerates podocyte injuries in db/db mice. Interestingly, no obvious urinary protein, inflammatory cytokine expression, or histological changes in the kidneys of casein-injected db/m mice were found compared with the db/m mice. CONCLUSION: An inflamed animal model of DN was successfully established and may provide a useful tool for investigating the pathogenesis of DN under inflammatory stress.

Maheshwari, R. A., et al. (2014). "Effect of coenzyme Q10 alone and its combination with metformin on streptozotocin-nicotinamide-induced diabetic nephropathy in rats." Indian J Pharmacol 46(6): 627-632.
Control: N = 6 Model: N = 6

OBJECTIVES: This study was aimed to investigate the therapeutic potential of coenzyme Q10 and its combination with metformin on streptozotocin (STZ)-nicotinamide-induced diabetic nephropathy (DN). MATERIALS AND METHODS: Type 2 diabetes in rats was induced with STZ-nicotinamide. The diabetic rats were treated with coenzyme Q10 (10 mg/kg, p.o.) alone or coenzyme Q10 + metformin. Various parameters of renal function tests such as serum creatinine, urea, uric acid, and markers of oxidative stress such as renal malondialdehyde (MDA) level, superoxide dismutase (SOD), and catalase (CAT) activities were measured. Tumor necrosis factor-α (TNF-α), myeloperoxidase (MPO) activity, transforming growth factor-β (TGF-β), and nitrite content were estimated in renal tissues. All treated animal were subjected to histopathological changes of kidney. RESULT: Diabetic rats showed a significant reduction in renal function, which was reflected with an increase in serum urea, serum creatinine, uric acid. In addition, STZ-nicotinamide caused renal tubular damage with a higher MDA level, depletion of SOD and CAT activity and glutathione (GSH) level. Moreover, TNF-α, MPO activity, TGF-β, and nitrite content were significantly increased in diabetic rats, while treatment with coenzyme Q10 or metformin or their combination ameliorate STZ-nicotinamide induced renal damage due to improvement in renal function, oxidative stress, suppression of TNF-α, MPO activity, TGF-β and nitrite content along with histopathological changes. CONCLUSIONS: This finding suggests that the treatment with coenzyme Q10 or metformin showed significant renoprotective effect against STZ-nicotinamide-induced DN. However, concomitant administration of both showed a better renoprotective effect than coenzyme Q10 or metformin alone treatment.

Mohamed, R., et al. (2013). "Chronic administration of EP4-selective agonist exacerbates albuminuria and fibrosis of the kidney in streptozotocin-induced diabetic mice through IL-6." Lab Invest 93(8): 933-945.
Control: N = 8 Model: N = 8

Diabetic nephropathy is currently the most common cause of end-stage renal disease in the western world. Exacerbated inflammation of the kidney is known to contribute acceleration of nephropathy.

Despite increased COX-2-mediated production of prostanoid metabolite PGE2, knowledge on its involvement in the progression of diabetic kidney disease is not complete. Here, we show the cross talk of the PGE2-EP4 pathways and IL-6 in inducing albuminuria and fibrosis in an animal model of type 1 diabetes. Hyperglycemia causes enhanced COX-2 expression and PGE2 production. Administration of PGE2 receptor EP4-selective agonist ONO-AE1-329 for 12 weeks exacerbated fibrosis and albuminuria. Diabetes-induced expression of inflammatory cytokines TNFα and TGFβ1 was enhanced in EP4 agonist-treated mice kidney. In addition, urinary excretion of cytokines (TNFα and IL-6) and chemokines (MCP-1 and IP-10) were significantly more in EP4-treated mice than vehicle-treated diabetes. Diabetes-induced collagen I and CTGF expression were also significantly higher in EP4-treated mice. However, EP4 agonist did not alter macrophage infiltration but increased cytokine and chemokine production in RAW264.7 cells. Interestingly, EP4-induced IL-6 expression in the kidney was localized in proximal and distal tubular epithelial cells. To confirm further whether EP4 agonist increases fibrosis and albuminuria through an increase in IL-6 expression, IL-6-knockout mice were administered with EP4 agonist. IL-6-knockout mice were resistant to EP4-induced exacerbation of albuminuria and diabetes and EP4-induced fibrosis. Our data suggest that EP4 agonist through IL-6 induces glomerulosclerosis and interstitial fibrosis, and IL-6 represents a new factor in the EP4 pathway.

Moon, J. Y., et al. (2016). "The Dose-Dependent Organ-Specific Effects of a Dipeptidyl Peptidase-4 Inhibitor on Cardiovascular Complications in a Model of Type 2 Diabetes." PLoS One 11(3): e0150745.
Control: N = 8 Model: N = 8

OBJECTIVE: Although dipeptidyl peptidase-4 (DPP-4) inhibitors have been suggested to have a non-glucoregulatory protective effect in various tissues, the effects of long-term inhibition of DPP-4 on the micro- and macro-vascular complications of type 2 diabetes remain uncertain. The aim of the present study was to investigate the organ-specific protective effects of DPP-4 inhibitor in rodent model of type 2 diabetes. METHODS: Eight-week-old diabetic and obese db/db mice and controls (db/m mice) received vehicle or one of two doses of gemigliptin (0.04 and 0.4%) daily for 12 weeks. Urine albumin excretion and echocardiography measured at 20 weeks of age. Heart and kidney tissue were subjected to molecular analysis and immunohistochemical evaluation. RESULTS: Gemigliptin effectively suppressed plasma DPP-4 activation in db/db mice in a dose-dependent manner. The HbA1c level was normalized in the 0.4% gemigliptin, but not in the 0.04% gemigliptin group. Gemigliptin showed a dose-dependent protective effect on podocytes, anti-apoptotic and anti-oxidant effects in the diabetic kidney. However, the dose-dependent effect of gemigliptin on diabetic cardiomyopathy was ambivalent. The lower dose significantly attenuated left ventricular (LV) dysfunction, apoptosis, and cardiac fibrosis, but the higher dose could not protect the LV dysfunction and cardiac fibrosis. CONCLUSION: Gemigliptin exerted non-glucoregulatory protective effects on both diabetic nephropathy and cardiomyopathy. However, high-level inhibition of DPP-4 was associated with an organ-specific effect on cardiovascular complications in type 2 diabetes.

Morigi, M., et al. (2020). "C3a receptor blockade protects podocytes from injury in diabetic nephropathy." JCI Insight 5(5).
Control: N = 5 Model: N = 8

Renal activation of the complement system has been described in patients with diabetic nephropathy (DN), although its pathological relevance is still ill-defined. Here, we studied whether glomerular C3a, generated by uncontrolled complement activation, promotes podocyte damage, leading to proteinuria and renal injury in mice with type 2 diabetes. BTBR ob/ob mice exhibited podocyte loss, albuminuria, and glomerular injury accompanied by C3 deposits and increased C3a and C3a receptor (C3aR) levels. Decreased glomerular nephrin and α-actinin4 expression, coupled with integrin-linked kinase induction, were also observed. Treatment of DN mice with a C3aR antagonist enhanced podocyte density and preserved their phenotype, limiting proteinuria and glomerular injury. Mechanistically, ultrastructural and functional mitochondrial alterations, accompanied by downregulation of antioxidant superoxide dismutase 2 (SOD2) and increased protein oxidation, occurred in podocytes and were normalized by C3aR blockade. In cultured podocytes, C3a induced cAMP-dependent mitochondrial fragmentation. Alterations of mitochondrial membrane potential, SOD2 expression, and energetic metabolism were also found in response to C3a. Notably, C3a-induced podocyte motility was inhibited by SS-31, a peptide with mitochondrial protective effects. These data indicate that C3a blockade represents a potentially novel therapeutic strategy in DN for preserving podocyte integrity through the maintenance of mitochondrial functions.

Nasri, M., et al. (2020). "Exogenous glutamine ameliorates diabetic nephropathy in a rat model of type 2 diabetes mellitus through its antioxidant and anti-inflammatory activities." Arch Physiol Biochem: 1-10.

Control: N = 9 Model: N = 9

This study aimed to evaluate the effects of glutamine (Gln) on diabetic nephropathy and other complications in a rat model of type 2 diabetes mellitus. Streptozotocin/nicotinamide induced diabetic rats were enrolled as an animal model of type 2 diabetes mellitus. Animals were divided into control, diabetic, and Gln (1000 mg/l in drinking water, eight weeks) treated diabetic groups. Gln alleviated renal inflammatory and oxidative stress biomarkers (tumour necrosis factor-alpha, interleukin 6, glutathione peroxidase, total superoxide dismutase, and glutathione), decreased serum uric acid and creatinine, and restored renal histopathological changes (glomerular volume, sclerosis, and leukocyte infiltration). Additionally, Gln ameliorated other complications, including systemic oxidative stress (serum malondialdehyde and nitric oxide, serum and liver glutathione, glutathione peroxidase, and total superoxide dismutase, and liver catalase), insulin resistance, hyperglycaemia, and hyperlipidaemia. Collectively, Gln attenuates diabetic nephropathy and other complications in type 2 diabetes mellitus in rats through its antioxidant and anti-inflammatory activities.

Ndisang, J. F. and A. Jadhav (2014). "Hemin therapy improves kidney function in male streptozotocin-induced diabetic rats: role of the heme oxygenase/atrial natriuretic peptide/adiponectin axis." Endocrinology 155(1): 215-229.

Control: N = 6 Model: N = 6

Diabetic nephropathy is characterized by elevated macrophage infiltration and inflammation. Although heme-oxygenase (HO) is cytoprotective, its role in macrophage infiltration and nephropathy in type 1 diabetes is not completely elucidated. Administering the HO inducer, hemin, to streptozotocin-diabetic rats suppressed renal proinflammatory macrophage-M1 phenotype alongside several proinflammatory agents, chemokines, and cytokines including macrophage inflammatory protein 1α (MIP-1α), macrophage-chemoattractant protein-1 (MCP-1), TNF-α, IL-1β, IL-6, nuclear factor-κB (NF-κB), and aldosterone, a stimulator of the inflammatory/oxidative transcription factor, NF-κB. Similarly, hemin therapy attenuated extracellular matrix/profibrotic proteins implicated in renal injury including fibronectin, collagen-IV, and TGF-β1 and reduced several renal histopathological lesions such as glomerulosclerosis, tubular necrosis, tubular vacuolization, and interstitial macrophage infiltration. Furthermore, hemin reduced markers of kidney dysfunction like proteinuria and albuminuria but increased creatinine clearance, suggesting improved kidney function. Correspondingly, hemin significantly enhanced the antiinflammatory macrophage-M2 phenotype, IL-10, adiponectin, HO-1, HO activity, and atrial natriuretic-peptide (ANP), a substance that abates TNF-α, IL-6, and IL-1β, with parallel increase of urinary cGMP, a surrogate marker of ANP. Contrarily, coadministering the HO inhibitor, chromium-mesoporphyrin with the HO-inducer, hemin nullified the antidiabetic and renoprotective effects, whereas administering chromium-mesoporphyrin alone abrogated basal HO activity, reduced basal adiponectin and ANP levels, aggravated hyperglycemia, and further increased MCP-1, MIP-1α, aldosterone, NF-κB, TNF-α, IL-6, IL-1β, proteinuria/albuminuria, and aggravated creatinine clearance, thus exacerbating renal dysfunction, suggesting the importance of the basal HO-adiponectin-ANP axis in renoprotection and kidney function. Collectively, these data suggest that hemin ameliorates diabetic nephropathy by selectively enhancing the antiinflammatory macrophage-M2 phenotype and IL-10 while concomitantly abating the proinflammatory macrophage-M1 phenotype and suppressing extracellular matrix/profibrotic factors with reduction of renal lesions including interstitial macrophage infiltration. Because aldosterone stimulate NF-κB, which activates cytokines like TNF-α, IL-6, IL-1β that in turn stimulate chemokines such as MCP-1 and MIP-1α to promote macrophage-M1 infiltration, the hemin-dependent potentiation of the HO-adiponectin-ANP axis may account for reduced macrophage infiltration and inflammatory insults in streptozotocin-diabetic rats.

Nordquist, L., et al. (2015). "Activation of hypoxia-inducible factors prevents diabetic nephropathy." J Am Soc Nephrol 26(2): 328-338.

Control: N = 9 Model: N = 12

Hyperglycemia results in increased oxygen consumption and decreased oxygen tension in the kidney. We tested the hypothesis that activation of hypoxia-inducible factors (HIFs) protects against diabetes-induced alterations in oxygen metabolism and kidney function. Experimental groups consisted of control and streptozotocin-induced diabetic rats treated with or without chronic cobalt chloride to activate HIFs. We elucidated the involvement of oxidative stress by studying the effects of acute administration of the superoxide dismutase mimetic tempol. Compared with controls, diabetic rats displayed tissue hypoxia throughout the kidney, glomerular hyperfiltration, increased oxygen consumption, increased total mitochondrial leak respiration, and decreased tubular sodium transport efficiency. Diabetic kidneys showed proteinuria and tubulointerstitial damage. Cobalt chloride activated HIFs, prevented the diabetes-induced alterations in oxygen metabolism, mitochondrial leak respiration, and kidney function, and reduced proteinuria and tubulointerstitial damage. The beneficial effects of tempol were less pronounced after activation of HIFs, indicating improved oxidative stress status. In conclusion, activation of HIFs

prevents diabetes-induced alteration in kidney oxygen metabolism by normalizing glomerular filtration, which reduces tubular electrolyte load, preventing mitochondrial leak respiration and improving tubular transport efficiency. These improvements could be related to reduced oxidative stress and account for the reduced proteinuria and tubulointerstitial damage. Thus, pharmacologic activation of the HIF system may prevent development of diabetic nephropathy.

Nunes, S., et al. (2020). "Crescent-Like Lesions as an Early Signature of Nephropathy in a Rat Model of Prediabetes Induced by a Hypercaloric Diet." Nutrients 12(4).
Control: N = 8 Model: N = 8

Diabetic nephropathy (DN) is a major microvascular complication of diabetes. Obesity and hyperlipidemia, fueled by unhealthy food habits, are risk factors to glomerular filtration rate (GFR) decline and DN progression. Several studies recommend that diabetic patients should be screened early (in prediabetes) for kidney disease, in order to prevent advanced stages, for whom the current interventions are clearly inefficient. This ambition greatly depends on the existence of accurate early biomarkers and novel molecular targets, which only may arise with a more thorough knowledge of disease pathophysiology. We used a rat model of prediabetes induced by 23 weeks of high-sugar/high-fat (HSuHF) diet to characterize the phenotype of early renal dysfunction and injury. When compared with the control animals, HSuHF-treated rats displayed a metabolic phenotype compatible with obese prediabetes, displaying impaired glucose tolerance and insulin sensitivity, along with hypertriglyceridemia, and lipid peroxidation. Despite unchanged creatinine levels, the prediabetic animals presented glomerular crescent-like lesions, accompanied by increased kidney Oil-Red-O staining, triglycerides content and mRNA expression of IL-6 and iNOS. This model of HSuHF-induced prediabetes can be a useful tool to study early features of DN, namely crescent-like lesions, an early signature that deserves in-depth elucidation.

Pichaiwong, W., et al. (2013). "Reversibility of structural and functional damage in a model of advanced diabetic nephropathy." J Am Soc Nephrol 24(7): 1088-1102.
Control: N = 5 Model: N = 6

The reversibility of diabetic nephropathy remains controversial. Here, we tested whether replacing leptin could reverse the advanced diabetic nephropathy modeled by the leptin-deficient BTBR ob/ob mouse. Leptin replacement, but not inhibition of the renin-angiotensin-aldosterone system (RAAS), resulted in near-complete reversal of both structural (mesangial matrix expansion, mesangiolysis, basement membrane thickening, podocyte loss) and functional (proteinuria, accumulation of reactive oxygen species) measures of advanced diabetic nephropathy. Immunohistochemical labeling with the podocyte markers Wilms tumor 1 and p57 identified parietal epithelial cells as a possible source of regenerating podocytes. Thus, the leptin-deficient BTBR ob/ob mouse provides a model of advanced but reversible diabetic nephropathy for further study. These results also suggest that restoration of lost podocytes is possible but is not induced by RAAS inhibition, possibly explaining the limited efficacy of RAAS inhibitors in promoting repair of diabetic nephropathy.

Sadar, S., et al. (2016). "Protective effect of L-glutamine against diabetes-induced nephropathy in experimental animal: Role of KIM-1, NGAL, TGF-β1, and collagen-1." Ren Fail 38(9): 1483-1495.
Control: N = 5 Model: N = 5

Diabetic nephropathy is a serious microvascular complication and one of the main causes of end-stage renal disease. L-Glutamine (LG) is naturally occurring amino acids with antidiabetic and antioxidant potential. The aim of present investigation was to evaluate the potential of LG against streptozotocin (STZ)-induced diabetic nephropathy (DN) in laboratory rats. DN was induced in male Wistar rats (200-220 g) by intraperitoneal administration of STZ (55 mg/kg). Animals were treated orally with either distilled water (10 mg/kg) or LG (250, 500, and 1000 mg/kg) or Sitagliptin (5 mg/kg). Various biochemical, molecular, and histological (hematoxylin-eosin and Masson's trichrome stain) parameters were assessed. Administration of LG (500 and 1000 mg/kg) significantly inhibited ($p < .05$) STZ-induced alterations in serum and urine biochemistry (urine creatinine, uric acid, albumin, and BUN). It also significantly increased creatinine clearance rate. STZ induced increase in renal oxidonitrosative stress was significantly decreased ($p < .05$) by LG (500 and 1000 mg/kg) treatment. Upregulated renal KIM-1, NGAL, TGF-β1, and collagen-1 mRNA expression after STZ administration was significantly inhibited ($p < .05$) by LG (500 and 1000 mg/kg) treatment. Correlation analysis also revealed that antidiabetic potential of LG

attenuates STZ-induced elevated renal KIM-1, NGAL, TGF-β1, and collagen-1 mRNA expression. Histopathological alteration induced by STZ in renal tissue was ameliorated by LG treatment. In conclusion, results of present investigation suggest that treatment with LG ameliorated STZ-induced DN via the inhibition of oxidonitrosative stress as well as downregulation of KIM-1, NGAL, TGF-β1, and collagen-1 mRNA expressions.

Sathibabu Uddandrao, V. V., et al. (2019). "Restorative potentiality of S-allylcysteine against diabetic nephropathy through attenuation of oxidative stress and inflammation in streptozotocin-nicotinamide-induced diabetic rats." Eur J Nutr 58(6): 2425-2437.
Control: N = 6　　Model: N = 6

AIM: In the present study, we evaluated the therapeutic potentiality of S-allylcysteine (SAC) in streptozotocin (STZ)-nicotinamide (NAD)-induced diabetic nephropathy (DN) in experimental rats. METHODS: SAC was orally administered for 45 days to rats with STZ-NAD-induced DN; a metformin-treated group was included for comparison. Effect of SAC on body weight, organ weight, blood glucose, levels of insulin, glycated haemoglobin, and renal biochemical markers was determined. Body composition by total body electrical conductivity (TOBEC) and dual-X ray absorptiometry (DXA), kidney antioxidant analysis, real-time polymerase chain reaction, and western blot analysis of superoxide dismutase (SOD), catalase (CAT), glutathione peroxidase (GPx), nuclear factor kappa B (NF-κB), interleukin (IL)-6, and tumor necrosis factor (TNF)-α; histopathological and scanning electron microscope (SEM) analysis of the kidneys were performed in both control and experimental rats. RESULTS: SAC treatment showed significantly decreased levels of blood glucose, glycated haemoglobin, creatinine, albumin, AST, ALT, creatinine kinase, lactate dehydrogenase, and expressions of NF-κB, IL-6, and TNF-α compared with DN control rats. Furthermore, SAC administration to DN rats significantly improved body composition and antioxidant defense mechanism which was confirmed by the upregulation of mRNA and protein expressions of antioxidant genes. CONCLUSIONS: Thus, SAC showed adequate therapeutic effect against DN by downregulation of inflammatory factors and attenuation of oxidative stress. Histological and SEM observations also indicated that SAC treatment notably reverses renal damage and protects the kidneys from hyperglycemia-mediated oxidative damage.

Shiju, T. M., et al. (2013). "Renoprotective effect of aged garlic extract in streptozotocin-induced diabetic rats." Indian J Pharmacol 45(1): 18-23.
Control: N = 4　　Model: N = 4

OBJECTIVE: Aged garlic extract (AGE) has been proven to exhibit antioxidant, hypolipidemic, hypoglycemic and antidiabetic properties. However, its effect on diabetic nephropathy was unexplored. Therefore, the present study was designed to investigate the renoprotective effect of AGE in streptozotocin-induced diabetic rats. MATERIALS AND METHODS: Albino Wistar rats were induced with diabetes by a single intraperitoneal injection of 45 mg/kg b.w. of streptozotocin. Commercially available AGE was supplemented orally at a dose of 500 mg/kg body weight/day. Aminoguanidine, which has been proven to be an anti-glycation agent was used as positive control and was supplemented at a dose of 1 g/L in drinking water. The serum and urinary biochemical parameters were analyzed in all the groups and at the end of 12 weeks follow up, the renal histological examination were performed using H & E and PAS staining. RESULTS: The diabetic rats showed a significant change in the urine ($P < 0.001$) and serum ($P < 0.01$) constituents such as albumin, creatinine, urea nitrogen and glycated hemoglobin. In addition, the serum lipid profile of the diabetic rats were altered significantly ($P < 0.05$) compared to that of the control rats. However, the diabetic rats supplemented with aged garlic extract restored all these biochemical changes. The efficacy of the extract was substantiated by the histopathological changes in the kidney. CONCLUSION: From our results, we conclude that aged garlic extract has the ability to ameliorate kidney damage in diabetic rats and the renoprotective effect of AGE may be attributed to its anti-glycation and hypolipidemic activities.

Somineni, H. K., et al. (2014). "Daily exercise training protects against albuminuria and angiotensin converting enzyme 2 shedding in db/db diabetic mice." J Endocrinol 221(2): 235-251.
Control: N = 6　　Model: N = 6

Angiotensin II (Ang II) is involved in induction and progression of renal damage in diabetes. Angiotensin converting enzyme 2 (ACE2) is highly expressed in the kidney and has been shown to be renoprotective by degrading Ang II to Ang-(1-7). A disintegrin and metalloproteinase 17 (ADAM17)-mediated shedding of renal ACE2 contribute to diabetic nephropathy pathogenesis. Lifestyle modification and metformin are recommended as initial therapies for most patients with type 2 diabetes. The aim of this study was to investigate whether exercise training and/or metformin improve glucose homeostasis and albuminuria and downregulate renal ADAM17 and ACE2 shedding in db/db mice. Seven-week-old

normal and db/db mice were subjected either to a sedentary existence or exercise training with and without metformin (150 mg/kg per day) for 10 weeks. Exercise training significantly lowered blood glucose, urinary albumin and ACE2 excretion in db/db mice. ADAM17 and ACE2 proteins were co-localized in cortical tubules of the kidney, indicating a possible interaction. Metformin treatment was effective in lowering hyperglycemia only during the first 2 weeks of treatment. Increased renal ADAM17 in 17-week-old db/db mice was corrected by physical exercise but not metformin. In addition, exercise training reduced plasma triglycerides and enhanced insulin levels of db/db mice. In conclusion, exercise training alone and in combination with metformin prevented shedding of renal ACE2 by decreasing ADAM17 protein. Urinary ACE2 could serve as a prognostic tool for the progression of kidney damage and its attenuation by exercise may partially contribute to its renal protection.

Souza, C. S., et al. (2019). "Preventive effect of exercise training on diabetic kidney disease in ovariectomized rats with type 1 diabetes." Exp Biol Med (Maywood) 244(9): 758-769.
Control: N = 8 Model: N = 8

To date, no studies have been found evaluating the effects of physical exercise on renal function and structure changes in ovariectomized rats with type 1 diabetes. Therefore, this work emerges with an important tool for strengthening and expanding innovative research on exercise with potential for the prevention of renal diseases in ovariectomized diabetic rats, and future development of studies that seek to increase scientific knowledge about the beneficial effects of physical exercise on renal diseases in humans.

te Riet, L., et al. (2014). "Deterioration of kidney function by the (pro)renin receptor blocker handle region peptide in aliskiren-treated diabetic transgenic (mRen2)27 rats." Am J Physiol Renal Physiol 306(10): F1179-1189.
Control: N = 8 Model: N = 8

Dual renin-angiotensin system (RAS) blockade in diabetic nephropathy is no longer feasible because of the profit/side effect imbalance. (Pro)renin receptor [(P)RR] blockade with handle region peptide (HRP) has been reported to exert beneficial effects in various diabetic models in a RAS-independent manner. To what degree (P)RR blockade adds benefits on top of RAS blockade is still unknown. In the present study, we treated diabetic TGR(mREN2)27 rats, a well-established nephropathy model with high prorenin levels [allowing continuous (P)RR stimulation in vivo], with HRP on top of renin inhibition with aliskiren. Aliskiren alone lowered blood pressure and exerted renoprotective effects, as evidenced by reduced glomerulosclerosis, diuresis, proteinuria, albuminuria, and urinary aldosterone levels as well as diminished renal (P)RR and ANG II type 1 receptor expression. It also suppressed plasma and tissue RAS activity and suppressed cardiac atrial natriuretic peptide and brain natriuretic peptide expression. HRP, when given on top of aliskiren, did not alter the effects of renin inhibition on blood pressure, RAS activity, or aldosterone. However, it counteracted the beneficial effects of aliskiren in the kidney, induced hyperkalemia, and increased plasma plasminogen activator-inhibitor 1, renal cyclooxygenase-2, and cardiac collagen content. All these effects have been linked to (P)RR stimulation, suggesting that HRP might, in fact, act as a partial agonist. Therefore, the use of HRP on top of RAS blockade in diabetic nephropathy is not advisable.

Thibodeau, J. F., et al. (2014). "A novel mouse model of advanced diabetic kidney disease." PLoS One 9(12): e113459.
Control: N = 5 Model: N = 5

Currently available rodent models exhibit characteristics of early diabetic nephropathy (DN) such as hyperfiltration, mesangial expansion, and albuminuria yet features of late DN (hypertension, GFR decline, tubulointerstitial fibrosis) are absent or require a significant time investment for full phenotype development. Accordingly, the aim of the present study was to develop a mouse model of advanced DN with hypertension superimposed (HD mice). Mice transgenic for human renin cDNA under the control of the transthyretin promoter (TTRhRen) were employed as a model of angiotensin-dependent hypertension. Diabetes was induced in TTRhRen mice through low dose streptozotocin (HD-STZ mice) or by intercrossing with OVE26 diabetic mice (HD-OVE mice). Both HD-STZ and HD-OVE mice displayed more pronounced increases in urinary albumin levels as compared with their diabetic littermates. Additionally, HD mice displayed renal hypertrophy, advanced glomerular scarring and evidence of tubulointerstitial fibrosis. Both HD-OVE and HD-STZ mice showed evidence of GFR decline as FITC-inulin clearance was decreased compared to hyperfiltering STZ and OVE mice. Taken together our results

suggest that HD mice represent a robust model of type I DN that recapitulates key features of human disease which may be significant in studying the pathogenesis of DN and in the assessment of putative therapeutics.

Toyoda, K., et al. (2018). "High fructose diet feeding accelerates diabetic nephropathy in Spontaneously Diabetic Torii (SDT) rats." J Toxicol Sci 43(1): 45-58.
Control: N = 3 Model: N = 3

Diabetic nephropathy (DN) is one of the complications of diabetes and is now the most common cause of end-stage renal disease. Fructose is a simple carbohydrate that is present in fruits and honey and is used as a sweetener because of its sweet taste. Fructose has been reported to have the potential to progress diabetes and DN in humans even though fructose itself does not increase postprandial plasma glucose levels. In this study, we investigated the effects of high fructose intake on the kidney of the Spontaneously Diabetic Torii (SDT) rats which have renal lesions similar to those in DN patients and compared these with the effects in normal SD rats. This study revealed that a 4-week feeding of the high fructose diet increased urinary excretion of kidney injury makers for tubular injury and accelerated mainly renal tubular and interstitial lesions in the SDT rats but not in normal rats. The progression of the nephropathy in the SDT rats was considered to be related to increased internal uric acid and blood glucose levels due to the high fructose intake. In conclusion, high fructose intake exaggerated the renal lesions in the SDT rats probably due to effects on the tubules and interstitium through metabolic implications for uric acid and glucose.

Tung, C. W., et al. (2019). "MicroRNA-29a Attenuates Diabetic Glomerular Injury through Modulating Cannabinoid Receptor 1 Signaling." Molecules 24(2).
Control: N = 6 Model: N = 6

Diabetic nephropathy often leads to end-stage renal disease and life-threatening morbidities. Simple control of risk factors is insufficient to prevent the progression of diabetic nephropathy, hence the need for discovering new treatments is of paramount importance. Recently, the dysregulation of microRNAs or the cannabinoid signaling pathway has been implicated in the pathogenesis of various renal tubulointerstitial fibrotic damages and thus novel therapeutic targets for chronic kidney diseases have emerged; however, the role of microRNAs or cannabinoid receptors on diabetes-induced glomerular injuries remains to be elucidated. In high-glucose-stressed renal mesangial cells, transfection of a miR-29a precursor sufficiently suppressed the mRNA and protein expressions of cannabinoid type 1 receptor (CB1R). Our data also revealed upregulated CB1R, interleukin-1β, interleukin-6, tumor necrosis factor-α, c-Jun, and type 4 collagen in the glomeruli of streptozotocin (STZ)-induced diabetic mice, whereas the expression of peroxisome proliferator-activated receptor-γ (PPAR-γ) was decreased. Importantly, using gain-of-function transgenic mice, we demonstrated that miR-29a acts as a negative regulator of CB1R, blocks the expressions of these proinflammatory and profibrogenic mediators, and attenuates renal hypertrophy. We also showed that overexpression of miR-29a restored PPAR-γ signaling in the renal glomeruli of diabetic animals. Collectively, our findings indicate that the interaction between miR-29a, CB1R, and PPAR-γ may play an important role in protecting diabetic renal glomeruli from fibrotic injuries.

Uil, M., et al. (2018). "Combining streptozotocin and unilateral nephrectomy is an effective method for inducing experimental diabetic nephropathy in the 'resistant' C57Bl/6J mouse strain." Sci Rep 8(1): 5542.
Control: N = 10 Model: N = 7

Diabetic nephropathy (DN) is the leading cause of chronic kidney disease. Animal models are essential tools for designing new strategies to prevent DN. C57Bl/6 (B6) mice are widely used for transgenic mouse models, but are relatively resistant to DN. This study aims to identify the most effective method to induce DN in a type 1 (T1D) and a type 2 diabetes (T2D) model in B6 mice. For T1D-induced DN, mice were fed a control diet, and randomised to streptozotocin (STZ) alone, STZ+unilateral nephrectomy (UNx), or vehicle/sham. For T2D-induced DN, mice were fed a western (high fat) diet, and randomised to either STZ alone, STZ+UNx, UNx alone, or vehicle/sham. Mice subjected to a control diet with STZ +UNx developed albuminuria, glomerular lesions, thickening of the glomerular basement membrane, and tubular injury. Mice on control diet and STZ developed only mild renal lesions. Furthermore, kidneys from mice on a western diet were hardly affected by diabetes, UNx or the combination. We conclude that STZ combined with UNx is the most effective model to induce T1D-induced DN in B6 mice. In our hands, combining western diet and STZ treatment with or without UNx did not result in a T2D-induced DN model in B6 mice.

Wang, L., et al. (2014). "Augmenting podocyte injury promotes advanced diabetic kidney disease in Akita mice." Biochem Biophys Res Commun 444(4): 622-627.
Control: N = 9   Model: N = 5

To determine if augmenting podocyte injury promotes the development of advanced diabetic nephropathy (DN), we created mice that expressed the enzyme cytosine deaminase (CD) specifically in podocytes of diabetic Akita mice (Akita-CD mice). In these mice, treatment with the prodrug 5-flucytosine (5-FC) causes podocyte injury as a result of conversion to the toxic metabolite 5-fluorouracil (5-FU). We found that treatment of 4-5 week old Akita mice with 5-FC for 5 days caused robust albuminuria at 16 and 20 weeks of age compared to 5-FC treated Akita controls, which do not express CD (Akita CTLs). By 20 weeks of age, there was a significant increase in mesangial expansion in Akita-CD mice compared to Akita CTLs, which was associated with a variable increase in glomerular basement membrane (GBM) width and interstitial fibrosis. At 20 weeks of age, podocyte number was similarly reduced in both groups of Akita mice, and was inversely correlated with the albuminuria and mesangial expansion. Thus, enhancing podocyte injury early in the disease process promotes the development of prominent mesangial expansion, interstitial fibrosis, increased GBM thickness and robust albuminuria. These data suggest that podocytes play a key role in the development of advanced features of diabetic kidney disease.

Wang, X., et al. (2019). "An experimental study of exenatide effects on renal injury in diabetic rats1." Acta Cir Bras 34(1): e20190010000001.
Control: N = 10   Model: N = 10

PURPOSE: To investigate the effects of exenatide on renal injury in streptozotocin-induced diabetic rats. METHODS: Fifty SD rats were randomly divided into normal control, model, exenatide-1, exenatide-2 and exenatide-3 groups, 10 rats in each group. The diabetic nephropathy model was constructed in later 4 groups. Then, the later 3 groups were treated with 2, 4 and 8 μg/kg exenatide for 8 weeks, respectively. The serum and urine biochemical indexes and oxidative stress and inflammatory indexes in renal tissue were determined. RESULTS: Compared to the model group, in exenatide-3 group the serum fasting plasma glucose and hemoglobin A1c levels were significantly decreased, the fasting insulin level was significantly increased, the renal index and blood urea nitrogen, serum creatinine and 24 h urine protein levels were significantly decreased, the renal tissue superoxide dismutase and glutathione peroxidase levels were significantly increased, the malondialdehyde level was significantly decreased, and the renal tissue tumor necrosis factor alpha, interleukin 6, hypersensitive C-reactive protein and chemokine (C-C motif) ligand 5 levels were significantly decreased $P<0.05$). CONCLUSIONS: Exenatide can mitigate the renal injury in diabetic rats. The mechanisms may be related to its resistance of oxidative stress and inflammatory response in renal tissue.

Wang, X. X., et al. (2017). "SGLT2 Protein Expression Is Increased in Human Diabetic Nephropathy: SGLT2 PROTEIN INHIBITION DECREASES RENAL LIPID ACCUMULATION, INFLAMMATION, AND THE DEVELOPMENT OF NEPHROPATHY IN DIABETIC MICE." J Biol Chem 292(13): 5335-5348.
Control: N = 12   Model: N = 12

There is very limited human renal sodium gradient-dependent glucose transporter protein (SGLT2) mRNA and protein expression data reported in the literature. The first aim of this study was to determine SGLT2 mRNA and protein levels in human and animal models of diabetic nephropathy. We have found that the expression of SGLT2 mRNA and protein is increased in renal biopsies from human subjects with diabetic nephropathy. This is in contrast to db-db mice that had no changes in renal SGLT2 protein expression. Furthermore, the effect of SGLT2 inhibition on renal lipid content and inflammation is not known. The second aim of this study was to determine the potential mechanisms of beneficial effects of SGLT2 inhibition in the progression of diabetic renal disease. We treated db/db mice with a selective SGLT2 inhibitor JNJ 39933673. We found that SGLT2 inhibition caused marked decreases in systolic blood pressure, kidney weight/body weight ratio, urinary albumin, and urinary thiobarbituric acid-reacting substances. SGLT2 inhibition prevented renal lipid accumulation via inhibition of carbohydrate-responsive element-binding protein-β, pyruvate kinase L, SCD-1, and DGAT1, key transcriptional factors and enzymes that mediate fatty acid and triglyceride synthesis. SGLT2 inhibition also prevented inflammation via inhibition of CD68 macrophage accumulation and expression of p65, TLR4, MCP-1, and osteopontin. These effects were associated with reduced mesangial expansion, accumulation of the extracellular matrix proteins fibronectin and type IV collagen, and loss of podocyte markers WT1 and synaptopodin, as determined by immunofluorescence microscopy. In summary, our study showed that SGLT2 inhibition modulates renal lipid metabolism and inflammation and prevents the development of nephropathy in

db/db mice.

Xin, R., et al. (2018). "Apocynin inhibited NLRP3/XIAP signalling to alleviate renal fibrotic injury in rat diabetic nephropathy." Biomed Pharmacother 106: 1325-1331.
Control: N = 9 Model: N = 9

AIMS: In this animal study, we tried to test the hypothesis that apocynin could play an anti-inflammation role by inhibiting NLRP3/X-linked inhibitor of apoptosis protein (XIAP) signalling and have an effect on antifibrosis in rats with diabetic nephropathy. MAIN METHODS: Diabetic nephropathy rats were induced by tail-vein injection of streptozotocin at 60 mg/kg body weight in sodium citrate buffer (0.01 M, pH 4.5) with unrestricted access to food/water for 12 weeks, and rats with blood glucose levels above 18.0 mM were considered diabetic; the damage index for glomerular mesangial cells damage index was calculated by morphological examinations; protein and mRNA changes were analysed by western blotting immunohistochemistry and real-time quantitative polymerase chain reaction; interstitial fibrosis was assessed and scored using Masson's staining. KEY FINDINGS: In rats with diabetic nephropathy, apocynin (1) reduced renal injury and improved renal function; (2) downregulated the expression of NLRP3 in renal cortex; (3) downregulated the expression of XIAP in renal cortex; and (4) attenuated renal fibrosis. SIGNIFICANCE: As an inhibitor of reactive oxygen species (ROS), apocynin could downregulate the expression of NLRP3 and XIAP, and alleviate renal fibrosis, which meant not only that ROS was one type of ligands of NLRP3, but also that ROS mechanism and NLRP3 activation might be therapeutic targets in the treatment of diabetic nephropathy in the future.

Xu, J., et al. (2014). "Effect of bilirubin on triglyceride synthesis in streptozotocin-induced diabetic nephropathy." J Korean Med Sci 29 Suppl 2(Suppl 2): S155-163.
Control: N = 5 Model: N = 8

We aimed to elucidate the effect of bilirubin on dyslipidemia and nephropathy in a diabetes mellitus (DM) type I animal model. Sprague-Dawley rats were separated into control, DM, and bilirubin-treated DM (Bil) groups. The Bil group was injected intraperitoneally with 60 mg/kg bilirubin 3 times per week and hepatoma cells were cultured with bilirubin at a concentration of 0.3 mg/dL. The Bil group showed lower serum creatinine levels 5 weeks after diabetes onset. Bilirubin treatment also decreased the amount of mesangial matrix, lowered the expression of renal collagen IV and transforming growth factor (TGF)-β1, and reduced the level of apoptosis in the kidney, compared to the DM group. These changes were accompanied by decreased tissue levels of hydrogen superoxide and NADPH oxidase subunit proteins. Bilirubin decreased serum total cholesterol, high-density lipoprotein cholesterol (HDL-C), free fatty acids, and triglycerides (TGs), as well as the TG content in the liver tissues. Bilirubin suppressed protein expression of LXRα, SREBP-1, SCD-1, and FAS, factors involved in TG synthesis that were elevated in the livers of DM rats and hepatoma cells under high-glucose conditions. In conclusion, bilirubin attenuates renal dysfunction and dyslipidemia in diabetes by suppressing LXRα and SREBP-1 expression and oxidative stress.

Yang, S., et al. (2016). "Amelioration of Diabetic Mouse Nephropathy by Catalpol Correlates with Down-Regulation of Grb10 Expression and Activation of Insulin-Like Growth Factor 1 / Insulin-Like Growth Factor 1 Receptor Signaling." PLoS One 11(3): e0151857.
Control: N = 6 Model: N = 6

Growth factor receptor-bound protein 10 (Grb10) is an adaptor protein that can negatively regulate the insulin-like growth factor 1 receptor (IGF-1R). The IGF1-1R pathway is critical for cell growth and apoptosis and has been implicated in kidney diseases; however, it is still unknown whether Grb10 expression is up-regulated and plays a role in diabetic nephropathy. Catalpol, a major active ingredient of a traditional Chinese medicine, Rehmannia, has been reported to possess anti-inflammatory and anti-aging activities and then used to treat diabetes. Herein, we aimed to assess the therapeutic effect of catalpol on a mouse model diabetic nephropathy and the potential role of Grb10 in the pathogenesis of this diabetes-associated complication. Our results showed that catalpol treatment improved diabetes-associated impaired renal functions and ameliorated pathological changes in kidneys of diabetic mice. We also found that Grb10 expression was significantly elevated in kidneys of diabetic mice as compared with that in non-diabetic mice, while treatment with catalpol significantly abrogated the elevated Grb10 expression in diabetic kidneys. On the contrary, IGF-1 mRNA levels and IGF-1R phosphorylation were significantly higher in kidneys of catalpol-treated diabetic mice than those in non-treated diabetic mice. Our results suggest that elevated Grb10 expression may play an important role in the pathogenesis of

diabetic nephropathy through suppressing IGF-1/IGF-1R signaling pathway, which might be a potential molecular target of catalpol for the treatment of this diabetic complication.

Ying, C., et al. (2021). "Ras-Related C3 Botulinum Toxin Substrate 1 Combining With the Mixed Lineage Kinase 3- Mitogen-Activated Protein Kinase 7- c-Jun N-Terminal Kinase Signaling Module Accelerates Diabetic Nephropathy." Front Physiol 12: 679166.
Control: N = 8 Model: N = 8

Ras-related C3 botulinum toxin substrate 1 (RAC1) activation plays a vital role in diabetic nephropathy (DN), but the exact mechanism remains unclear. In this study, we attempted to elucidate the precise mechanism of how RAC1 aggravates DN through cellular and animal experiments. In this study, DN was induced in mice by intraperitoneal injection of streptozotocin (STZ, 150mg/kg), and the RAC1 inhibitor NSC23766 was administered by tail vein injection. Biochemical indicators, cell proliferation and apoptosis, and morphological changes in the kidney were detected. The expression of phosphorylated c-Jun N-terminal kinase (p-JNK), nuclear factor-κB (NF-κB), and cleaved caspase-3 and the interaction between RAC1 and the mixed lineage kinase 3 (MLK3)-mitogen-activated protein kinase 7 (MKK7)-JNK signaling module were determined. Furthermore, the colocalization and direct co-interaction of RAC1 and MLK3 were confirmed. Our results showed that RAC1 accelerates renal damage and increases the expression of p-JNK, NF-κB, and cleaved caspase-3. However, inhibition of RAC1 ameliorated DN by downregulating p-JNK, NF-κB, and cleaved caspase-3. Also, RAC1 promoted the assembly of MLK3-MKK7-JNK, and NSC23766 blocked the interaction between RAC1 and MLK3-MKK7-JNK and inhibited the assembly of the MLK3-MKK7-JNK signaling module. Furthermore, RAC1 was combined with MLK3 directly, but the RAC1 Y40C mutant inhibited the interaction between RAC1 and MLK3. We demonstrated that RAC1 combining with MLK3 activates the MLK3-MKK7-JNK signaling module, accelerating DN occurrence and development, and RAC1 Y40 is an important site for binding of RAC1 to MLK3. This study illustrates the cellular and molecular mechanisms of how RAC1 accelerates DN and provides evidence of DN-targeted therapy.

Zakaria, E. M., et al. (2017). "PARP inhibition ameliorates nephropathy in an animal model of type 2 diabetes: focus on oxidative stress, inflammation, and fibrosis." Naunyn Schmiedebergs Arch Pharmacol 390(6): 621-631.
Control: N = 8 Model: N = 8

Poly(ADP-ribose) polymerase (PARP) enzyme contributes to nephropathy, a serious diabetic complication which may lead to end-stage renal disease. The study aims to investigate the effect of PARP over-activation on kidney functions in a type 2 diabetic rat model. The study also tests the therapeutic use of PARP inhibitors in diabetic nephropathy. Type 2 diabetes was induced in adult male rats by high-fructose/high-fat diet and low streptozotocin dose. Then, the PARP inhibitor 4-aminobenzamide (4-AB) was administered daily for 10 weeks. At the end, urine samples were collected to measure urine creatinine, albumin, and total proteins. PARP activity, superoxide dismutase (SOD) activity, and nitrite content were measured in kidney tissue homogenate. Glucose, fructosamine, insulin, and tumor necrosis factor-alpha (TNF-α) were measured in serum. Furthermore, histological studies, collagen deposition, and immunofluorescence of nuclear factor kappa B (NFκB) and transforming growth factor beta1 (TGF-β1) were carried out. PARP enzyme activity was significantly higher in the diabetic group and was significantly reduced by 4-AB administration. Diabetic animals had clear nephropathy indicated by proteinuria and increased albumin excretion rate (AER) which were significantly decreased by PARP inhibition. In addition, PARP inhibition increased creatinine clearance in diabetic animals and reduced renal TGF-β1 and glomerular fibrosis. Moreover, PARP inhibition alleviated the elevated serum TNF-α level, renal NFκB, nitrite, and the decrease in SOD activity in diabetic animals. However, PARP inhibition did not significantly affect neither hyperglycemia nor insulin sensitivity. PARP enzyme inhibition alleviates diabetic nephropathy through decreasing inflammation, oxidative stress, and renal fibrosis.

Zhang, H., et al. (2017). "Podocyte-specific JAK2 overexpression worsens diabetic kidney disease in mice." Kidney Int 92(4): 909-921.
Control: N = 9 Model: N = 8

Activation of JAK-STAT signaling has been implicated in the pathogenesis of diabetic kidney disease. An increased expression of JAK-STAT genes was found in kidney glomerular cells, including podocytes, in patients with early diabetic kidney disease. However, it is not known whether increased expression of JAK or STAT isoforms in glomerular cells can lead to worsening nephropathy in the setting of diabetes. Therefore, we overexpressed JAK2 mRNA specifically in glomerular podocytes of 129S6 mice to determine whether this change alone could worsen diabetic kidney disease. A 2-3 fold increase in glomerular JAK2

expression, an increase similar to that found in humans with early diabetic kidney disease, led to substantial and statistically significant increases in albuminuria, mesangial expansion, glomerulosclerosis, glomerular fibronectin accumulation, and glomerular basement membrane thickening, and a significant reduction in podocyte density in diabetic mice. Treatment with a specific JAK1/2 inhibitor for 2 weeks partly reversed the major phenotypic changes of diabetic kidney disease and specifically normalized expression of a number of downstream STAT3-dependent genes implicated in diabetic kidney disease progression. Thus, moderate increases in podocyte JAK2 expression at levels similar to those in patients with early diabetic kidney disease can lead directly to phenotypic and other alterations of progressive diabetic glomerulopathy. Hence, inhibition of these changes by treatment with a JAK1/2 inhibitor suggests that such treatment may help retard progression of early diabetic kidney disease in patients.

Zhang, X., et al. (2020). "Effects of ZnT8 on epithelial-to-mesenchymal transition and tubulointerstitial fibrosis in diabetic kidney disease." Cell Death Dis 11(7): 544.
Control: N = 8 Model: N = 8

Zinc transporter 8 (ZnT8) transports zinc ions for crystallization and storage of insulin in pancreatic beta-cells and ZnT8 dysfunction is involved in pathogenesis of diabetes. The current study aimed to investigate whether ZnT8 has effects in pathophysiology of diabetic kidney disease (DKD) by using animal models for diabetes, including STZ-induced diabetic, db/db, ZnT8-KO, ZnT8-KO-STZ and ZnT8-KO-db/db mice. Results demonstrated that urine albumin to creatinine ratio and epithelial-to-mesenchymal transition (EMT) were increased in kidneys of ZnT8-KO-STZ and ZnT8-KO-db/db mice compared with C57BL/6 J and ZnT8-KO mice, while serum TGF-β1, IL-6, and TNF-α levels were elevated in parallel. In kidneys of mice intercrossed between ZnT8-KO and STZ-induced diabetic or db/db mice, these three inflammatory factors, ACR and EMT were also found to be increased compared with C57BL/6J, db/db and ZnT8-KO mice. Furthermore, ZnT8 up-regulation by hZnT8-EGFP reduced the levels of high glucose (HG)-induced EMT and inflammatory factors in normal rat kidney tubular epithelial cell (NRK-52E cells). Expression of phosphorylated Smad2/Smad3 was up-regulated after HG stimulation and further enhanced by ZnT8 siRNA but down-regulated after hZnT8-EGFP gene transfection. The current study thus provides the first evidence that ZnT8 protects against EMT-tubulointerstitial fibrosis though the restrain of TGF-β1/Smads signaling activation in DKD.

Zhang, Y., et al. (2018). "Reversing CXCL10 Deficiency Ameliorates Kidney Disease in Diabetic Mice." Am J Pathol 188(12): 2763-2773.
Control: N = 8 Model: N = 8

The excessive accumulation of extracellular matrix material in the kidney is a histopathologic hallmark of diabetic kidney disease that correlates closely with declining function. Although considerable research has focused on the role of profibrotic factors, comparatively little attention has been paid to the possibility that a diminution in endogenous antifibrotic factors may also contribute. Among the latter, the ELR(-) CXC chemokines, CXCL9, CXCL10, and CXCL11, have been shown to provide a stop signal to prevent excessive fibrosis. Although the plasma concentrations of CXCL9 and CXCL11 were similar, those of CXCL10 were markedly lower in diabetic db/db mice compared with control db/m mice. In cell culture, CXCL10 inhibited kidney fibroblast collagen production in response to high glucose and the prosclerotic growth factor, transforming growth factor-β. In vivo, recombinant murine CXCL10 reduced mesangial and peritubular matrix expansion, albuminuria, and glomerular hypertrophy in db/db mice. In bone marrow, a major source of circulating chemokines, the concentration of CXCL10 was lower in cells derived from diabetic mice than from their nondiabetic counterparts. Silencing of CXCR3, the cognate receptor for CXCL10, abrogated the antifibrotic effects of bone marrow-derived secretions. In conclusion, experimental diabetes is a state of CXCL10 deficiency and that restoration of CXCL10 abundance prevented fibrosis and the development of diabetic kidney disease in mice.
